# A Personalized Therapeutics Approach Using an *In Silico Drosophila Patient Model* Reveals Optimal Chemo- and Targeted Therapy Combinations for Colorectal Cancer

**DOI:** 10.3389/fonc.2021.692592

**Published:** 2021-07-16

**Authors:** Mahnoor Naseer Gondal, Rida Nasir Butt, Osama Shiraz Shah, Muhammad Umer Sultan, Ghulam Mustafa, Zainab Nasir, Risham Hussain, Huma Khawar, Romena Qazi, Muhammad Tariq, Amir Faisal, Safee Ullah Chaudhary

**Affiliations:** ^1^ Biomedical Informatics Research Laboratory, Department of Biology, Lahore University of Management Sciences, Lahore, Pakistan; ^2^ Department of Pathology, Shaukat Khanum Memorial Cancer Hospital and Research Centre, Lahore, Pakistan; ^3^ Epigenetics Laboratory, Department of Biology, Lahore University of Management Sciences, Lahore, Pakistan; ^4^ Cancer Therapeutics Laboratory, Department of Biology, Lahore University of Management Sciences, Lahore, Pakistan

**Keywords:** personalized *in silico* cancer models, Boolean network models, cancer systems biology, preclinical *in silico* drug screening, combinatorial therapeutics

## Abstract

*In silico* models of biomolecular regulation in cancer, annotated with patient-specific gene expression data, can aid in the development of novel personalized cancer therapeutic strategies. *Drosophila melanogaster* is a well-established animal model that is increasingly being employed to evaluate such preclinical personalized cancer therapies. Here, we report five Boolean network models of biomolecular regulation in cells lining the *Drosophila* midgut epithelium and annotate them with colorectal cancer patient-specific mutation data to develop an *in silico Drosophila Patient Model* (DPM). We employed cell-type-specific RNA-seq gene expression data from the FlyGut-*seq* database to annotate and then validate these networks. Next, we developed three literature-based colorectal cancer case studies to evaluate cell fate outcomes from the model. Results obtained from analyses of the proposed DPM help: (i) elucidate cell fate evolution in colorectal tumorigenesis, (ii) validate cytotoxicity of nine FDA-approved CRC drugs, and (iii) devise optimal personalized treatment combinations. The personalized network models helped identify synergistic combinations of paclitaxel-regorafenib, paclitaxel-bortezomib, docetaxel-bortezomib, and paclitaxel-imatinib for treating different colorectal cancer patients. Follow-on therapeutic screening of six colorectal cancer patients from cBioPortal using this drug combination demonstrated a 100% increase in apoptosis and a 100% decrease in proliferation. In conclusion, this work outlines a novel roadmap for decoding colorectal tumorigenesis along with the development of personalized combinatorial therapeutics for preclinical translational studies.

## Introduction

Cancer development is a multistep process that is driven by a heterogeneous combination of somatic mutations at the genetic and epigenetic levels (1, 2). Specific mutations in oncogenes (3) and tumor suppressor genes (4), that result in their activation and inactivation, respectively, manifest themselves at the tissue level in the form of polyps, multi-layering, and metastasis (1, 5, 6). These system-level properties resulting from heterogeneous biomolecular aberrations and dysregulated cellular processes are abstracted as “*hallmarks of cancer”* (1, 6). The heterogeneity exhibited by cancer cells stems from factors such as genomic instability, clonal evolution, and variations in the microenvironment (7, 8). This fosters plasticity in cancer cells which lead to drug resistance – a leading impediment in the treatment of the disease (7–9). As a result, despite major research initiatives and resultant advancements in decoding the molecular basis of cancer, a comprehensive treatment for the disease still alludes researchers. The limited therapeutic regimens approved by the Food and Drug Administration (FDA) (10–12) exhibit variable efficacies across patients besides a multitude of toxic side effects and, multi-drug resistance (13). Towards designing efficacious personalized cancer therapeutics, recent advances in high-throughput omics-based approaches complemented by patient-specific gene expression data can provide significant assistance (14, 15). Several online databases and portals including cBioPortal (16), The Cancer Genome Atlas (TCGA) (17), and International Cancer Genome Consortium (ICGC) (18) amongst others (19, 20) provide such freely available datasets. However, effective and seamless utilization of such patient-specific genomic data to design personalized cancer therapies is still a fledgling area.

Researchers are increasingly employing whole-animal models (21–24) such a mouse, zebrafish, and fruit fly for preclinical *in vivo* validation of therapeutic hypotheses generated from personalized preclinical studies. Amongst the animal models, *Drosophila melanogaster* has become a popular platform for gene manipulation, investigating site-specific changes in the genome, and high-throughput whole-animal screening (14, 25). Importantly, a comparative study of the human and fly genome showed around 75% of disease-causing genes in humans are conserved in *Drosophila* (24, 26). Additionally, ease of handling and significantly lower genetic redundancy imparts further advantage to the employment of fly models (27). As a result, over 50 different data repositories, and tools are now available for hosting data on the fly genome, RNAi screens, and expression data including FlyGut*-seq* (28), and *FlyAtlas* (29) databases. Specifically in the case of cancer, several *in vivo* studies have been designed to elicit novel therapeutic targets using the *Drosophila* model system (30–33). One salient example is the validation of indomethacin, which is reported to enhance human Adenomatous Polyposis Coli (*hAPC*) induced phenotype in *Drosophila* eye (34) and therefore, employed for treating colorectal cancer (CRC). Vandetanib, another approved targeted therapy that was also validated by using the *Drosophila* system, suppressed Ret activity, and was later approved for medullary thyroid carcinoma (MTC) (30). However, a major shortcoming of using such mono-therapeutic agents for cancer treatment stems from the tumor heterogeneity which results in the selection of resistant cells (35, 36) besides acting specifically on singular pathways. To overcome these issues, multiple therapeutic agents acting on multiple pathways in synergy can significantly increase drug efficacy, besides lowering the therapeutic dosage (36). To evaluate high-efficacy synergistic drug combinations, researchers have employed the *Drosophila* model in preclinical studies to elicit optimal drug combinations (32, 33). The *Drosophila* Lung Cancer Model by Levine et al. (32) helped identify trametinib and fluvastatin as combinatorial drug therapy for lung cancer. Further, an EGFR induced lung tumor model was also designed in *Drosophila* which assisted in providing an alternative combination of drugs for lung cancer treatment through screening an FDA-approved compound library (33). However, combinatorial therapies pose unique challenges such as multidrug resistance in chemotherapy (13) and cross drug resistance (37, 38) besides the continuing need for higher therapeutic efficacies (39). Towards tackling these issues, researchers are now ‘personalizing’ live animal platforms for employment in preclinical studies to design efficacious therapeutic regimens. For instance, a comprehensive state-of-the-art *in vivo Drosophila Patient Model* (DPM) using a personalized therapeutics approach was described in flies (40). This particular study involved genetic manipulation of the fly genome to induce mutations specific to KRAS-mutant metastatic colorectal cancer. Combinatorial therapies were then given to the transgenic flies, harboring mutations that were identified in the patient, to discover high-efficacy synergistic drug combinations.

Here, we propose a novel computational framework in the form of an *in silico Drosophila Patient Model* (DPM), for developing personalized drug combinations for CRC patients. This framework is designed such that it can facilitate the modeling and analysis of patient-specified CRC network models along with evaluation of combinatorial therapeutic strategies (41, 42). We have constructed five biomolecular network models of cells regulating the maintenance of adult *Drosophila* midgut epithelium lining. These include multipotent intestinal stem cells (ISCs) (43–47), enteroblasts (EBs) (48), enterocytes (ECs), enteroendocrine cells (EEs) (49–53), and visceral muscle (VM) cells (54). Next, we evaluated each network’s ability to program cell fates under *normal* conditions as well as under minor perturbations. The ISCs are under the regulation of two sub-regions at the time of division; Apical and Basal (52). In our study, we have incorporated this information and analyzed ISC network under Apical and Basal regulation by changing inputs to the network. The networks including ISC’s under Apical and Basal regulation, EB, and EC, were then subjected to three types of inputs including physiological inputs (referred to as “*normal*”), aberrant inputs such that the fly homeostatic midgut regulation is perturbed (referred to as “*stress*”), and oncogenic inputs (referred to as “*cancer”*). The cell fate outcomes under *normal* and *cancer* conditions were validated against published literature. The individual output node propensities for the *normal* case were also validated against RNA-seq gene expression values taken from the FlyGut*-seq* (28) database. Additionally, three literature-based case studies were constructed to further validate the proposed *in silico* DPM. The first case study replicates colorectal tumorigenesis under progressive mutations using Martorell et al.’s CRC model (55). In the second case study, we employed Markstein et al.’s (56) model to perform therapeutic interventions to validate the cytotoxicity of nine FDA-approved drugs. Finally, in the third case study, we reproduced Bangi et al.’s KRAS-mutant CRC model (40) for evaluating optimal personalized drug treatment combinations by incorporating key patient-specific mutations into our model followed by combinatorial therapeutic screening. Building on these case studies, we devised a novel synergistic combination of a chemotherapeutic agent and a targeted therapy i.e., paclitaxel-regorafenib, paclitaxel-bortezomib, docetaxel-bortezomib, and paclitaxel-imatinib for treating six CRC patients taken from cBioPortal (16), while four patients were treated with only targeted therapy. The results obtained from combinatorial chemo- and targeted therapies show up to a 100% increase in anti-cancerous cell fates such as apoptosis and a 100% reduction in tumorigenesis promoting cell fates such as hyper-proliferation.

Taken together, we propose a computational framework in the form of an *in silico* DPM to provide personalized CRC therapeutics. This approach can help reduce the overall cancer treatment cost by facilitating the development of higher efficacy combinatorial therapies for colorectal cancer.

## Results

### Network Construction and Robustness Analysis of Regulatory Homeostasis in *Drosophila melanogaster* Midgut

**Figure 1:**
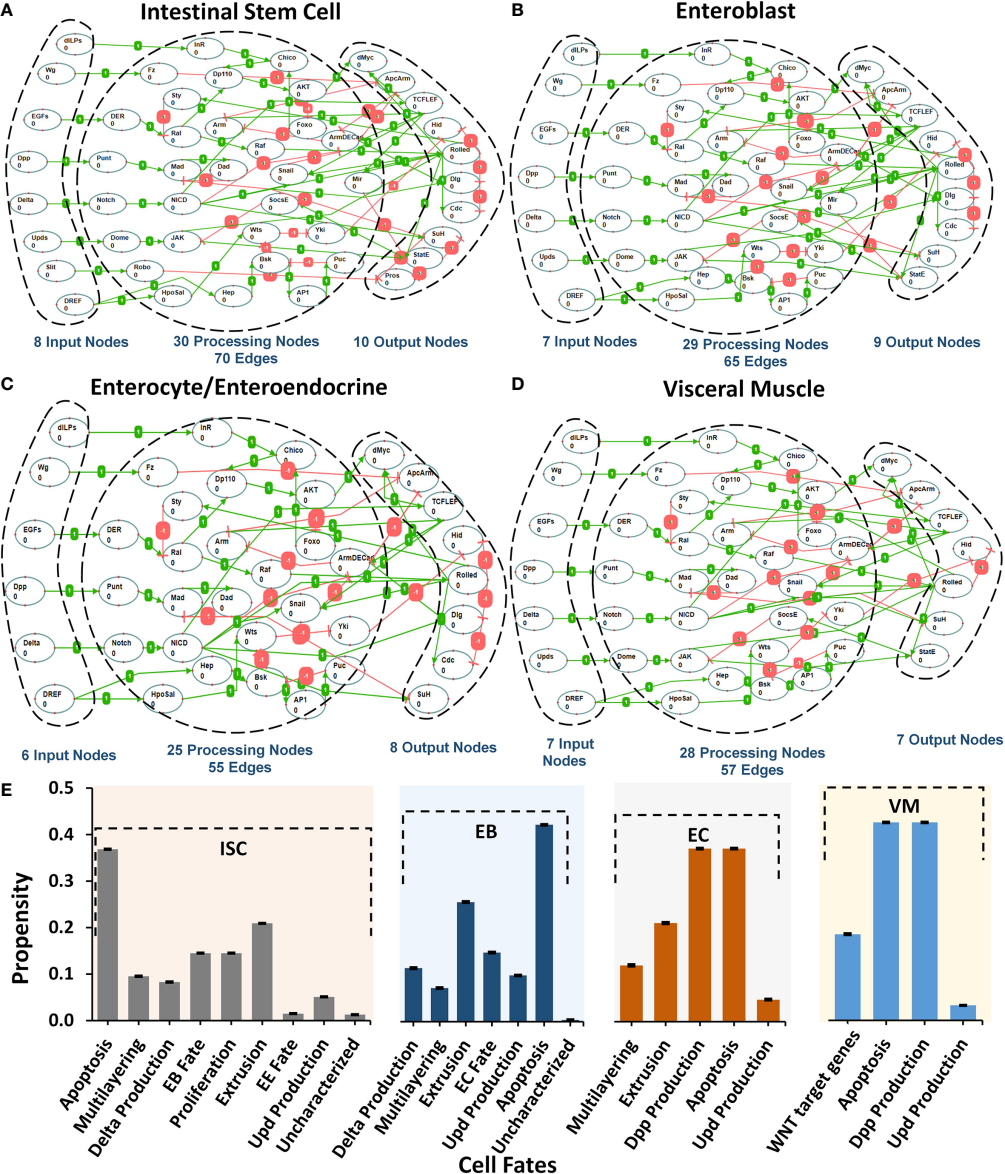
Regulatory schema of networks for the five cell types present in Drosophila melanogaster midgut. **(A–D)** The mapping between inputs, processing, and output nodes present in the biomolecular network models of five cell types i.e. ISC, EB, EC/EE, and VM. **(E)** Cellular fate propensities for ISC, EBs, ECs, and VM, along with their respective SEMs.

To investigate the biomolecular signaling regulating the homeostasis in *Drosophila melanogaster* midgut (**Supplementary Figure 1** **)**, we undertook an extensive literature survey and constructed five cell-type-specific rules-based network models (*details in* **Supplementary Tables 1–5**). Each model corresponds to one of the five cellular phenotypes lining the *Drosophila* midgut including intestinal stem cells (ISCs) (43–47), enteroblasts (EBs) (48), enterocytes (ECs), enteroendocrine cells (EEs) (49–53), and visceral muscle (VM) (54). The schematic of pathway integration in each network model is provided in **Supplementary Figures 2–6**. ISC network contains 48 nodes and 70 edges, EB consists of 45 nodes and 65 edges, EC and EE comprise 39 nodes and 55 edges, and VM contains 42 nodes and 57 edges (**Figures 1A–D**).

Next, to evaluate the biological plausibility of each network, we assessed the network response under *normal* input node values taken from the FlyGut*-seq* database (28) (*see Materials and Methods*). Our results show that the biomolecular network of ISC cells programmed apoptosis (with a propensity of 0.332), extrusion (0.188), proliferation (0.131), and differentiation/EB fate (0.131). EB network exhibited apoptosis (0.379), and extrusion (0.230). In the case of the EC network, apoptosis and dpp production were both programmed with propensities of 0.331, while for VM network apoptosis and dpp production cell fate program with 0.398 propensity (**Figure 1E** **)**.

To determine the robustness of cell fate programming by each type of cell, we induced a 10% perturbation in the input stimuli and observed the network response. The highest variation in cell fates was exhibited in apoptosis (SEM 0.0006), delta production (SEM 0.0012), multilayering (0.0014), and WNT target genes (0.0009) for ISC, EB, EC, and VM, respectively (**Supplementary Figure 7**). The robust cell fate programming results indicate that all five networks are biologically plausible as they exhibited robustness against random perturbations and are hence feasible for employment in onward analyses (57, 58) (**Supplementary Table 6**).

### Evaluation and Validation of Biomolecular Network Models Under *Normal, Stress* and *Colon Cancer* Conditions

**Figure 2:**
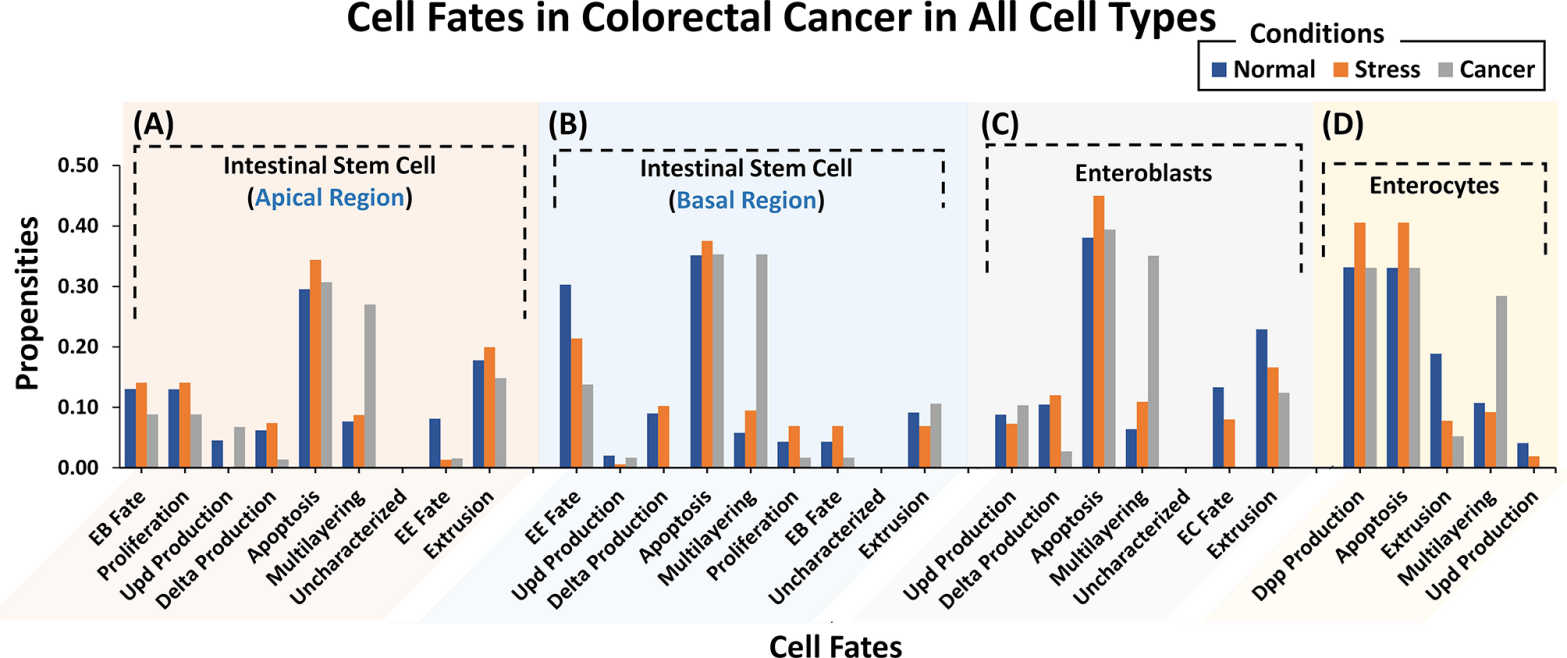
Cell fate propensities for intestinal stem cells (ISCs) under Apical and Basal regulation, enteroblasts (EBs), and enterocytes (ECs) in *normal, stress*, and *cancer* conditions. **(A)** ISC**’**s under Apical regulation adopt eight different cell fates in three ambient conditions. In *normal* conditions, the highest propensity was observed for apoptosis followed by extrusion, proliferation, and EB fate, in order. In the case of *stress*, the highest propensity is that of apoptosis, followed by extrusion, EB fate, and proliferation. In cancer, the highest propensity is that of apologies followed by multi-layering and extrusion. **(B)** ISCs under Basal regulation program eight different cell fates with the highest propensity being for apoptosis fate in *normal*, *stress*, and *cancer* conditions. **(C)** Six cellular fates in EB, with the highest propensity for apoptosis in *normal*, *stress*, and *cancer* conditions. **(D)** Five cellular fates in EC, with the highest propensity for dpp production and apoptosis in *normal, stress*, and *cancer* conditions. Uncharacterized cell fate has a 0.000 propensity in all conditions and every network.

To investigate and evaluate the proposed normal networks under *normal, stress*, and *cancerous* conditions (construed as a combination of inputs), Deterministic Analysis (DA) was performed (59) (**Supplementary Table 7**). Results from our analyses (**Figure 2**) revealed that under *normal* conditions, ISC’s Apical regulation programmed apoptosis, extrusion, proliferation, and differentiation (or EB fate) with propensities of 0.295, 0.178, 0.130, and 0.130, respectively (**Supplementary Table 8**) (see *Materials and Methods*). Under *stress* conditions, the propensity for proliferation, delta production, apoptosis, and differentiation increased to 0.141, 0.074 (from 0.062 in *normal* conditions), 0.344, and 0.141, respectively. Lastly, in *cancerous* conditions, propensities for multi-layering increased to 0.207, while proliferation, delta production decreased to 0.089 and 0.014, respectively. The results were again validated from the literature which supports that normal ISCs in *stress* conditions are known to undergo higher proliferation (60–62) and since delta is a marker for proliferation, its value increases as well (63–65). However, in the case of *cancer* conditions such as nutrient deprivation, etc., normal cells exhibit lowered proliferation (66, 67). Literature reports also that ISCs upon encountering extreme *stress*, exhibit epithelium multi-layering, augmented by overgrowth (68, 69) (**Supplementary Figures 8–10**).

For the ISC network under Basal regulation and in *normal* conditions (**Supplementary Table 7**), the cell fate outcomes included apoptosis, differentiation (or EE fate), and extrusion, with propensities of 0.353, 0.303, and 0.094, respectively (**Supplementary Table 8**). Under *stress*, apoptosis, proliferation, and delta production increased to 0.375, 0.069 (from 0.045 in *normal* conditions), and 0.102 (from 0.089 in *normal* conditions), respectively. For *cancer* conditions, the propensity of apoptosis, proliferation, and delta production decreased to 0.353, 0.017, and 0.000, respectively, whereas multi-layering increased to 0.353. Stressful cellular environments are known to increase the apoptosis rate (70–72). In absence of mutations, *normal* cells residing in toxic and oncogenic environments reduce their proliferation rate and delta production (63–67). Cell division rate, moreover, needs to be balanced with cell turnover and apoptosis so when proliferation slows down so does cell death (70, 71) (**Supplementary Figures 11–13**).

Next, we evaluated cell fate programming of the EB network under *normal* conditions (**Supplementary Table 7**). The results showed apoptosis, extrusion, and differentiation (or EC fate) cell fates with propensities of 0.381, 0.229, and 0.133, respectively (**Supplementary Table 8**). However, under *stress* conditions, the propensity for apoptosis and multi-layering increased to 0.450 and 0.109, respectively, whereas, extrusion and differentiation (or EC fate) decreased to 0.166, and 0.080, respectively. Under *cancerous* conditions, the salient cell fates programmed included multi-layering, apoptosis, and extrusion with propensities of 0.351, 0.394, and 0.124, respectively. Also, differentiation was suppressed to 0.000 due to toxic cellular environments. The trend in cell fate propensities under *cancerous* conditions also exhibited multi-layering (68, 69) along with low delta production and extrusion (**Supplementary Figures 14–16**). This corroborates with published literature stating that delta is a known marker for ISC and in the case of ISC proliferation, is reduced along with delta production (63–67) in *cancer* conditions.

Moreover, the EC network was also analyzed for response under *normal* conditions (**Supplementary Table 7**). The emergent cell fates included dpp production, apoptosis, and extrusion with propensities of 0.331, 0.331, and 0.189, respectively (**Supplementary Table 8**). Under *stress*, the extrusion rate decreased to 0.078, while dpp production and apoptosis both increased to 0.406, respectively. Dpp signaling is also known to increase under *stress* conditions to promote cell division (73). Under *cancer* conditions, however, an increase in propensities of multi-layering (0.284) was observed which is in agreement with published studies (68, 69) (**Supplementary Figures 17–19**).

**Figure 3:**
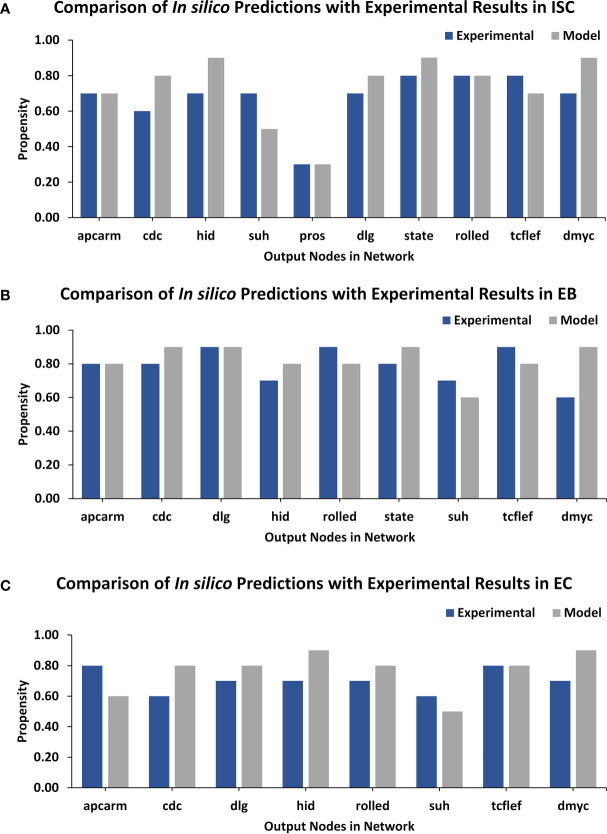
TISON output nodes propensities (*in silico* results) validation from FlyGut-seq database (*in vivo* results). **(A)** Comparison of ten output nodes propensities in ISC network: adenomatous polyposis coli (Apc2), cdc42 (Cdc42), head involution defective (hid), suppressor of hairless (Su(H)), prospero (pros), discs large 1 (dlg1), signal-transducer and activator of transcription protein at 92E (Stat92E), rolled (rl), pangolin (pan), and dMyc (myc). **(B)** Comparison of nine output nodes propensities in EB network: adenomatous polyposis coli (Apc2), cdc42 (Cdc42), discs large 1 (dlg1), head involution defective (hid), rolled (rl), signal-transducer, and activator of transcription protein at 92E (Stat92E), suppressor of hairless (Su(H)), pangolin (pan), and dMyc (myc). **(C)** Comparison of eight output nodes propensities in EC network: adenomatous polyposis coli (Apc2), cdc42 (Cdc42), discs large 1 (dlg1), head involution defective (hid), rolled (rl), suppressor of hairless (Su(H)), pangolin (pan) and dMyc (myc) (**Supplementary Table 10**).

Lastly, a comparison of output node values for ISC, EB, and EC networks under *normal* conditions was performed against experimental RNA-seq data from the FlyGut*-seq* database (28). Note that due to the paucity of regulatory dynamics in the literature on EE and VM, we could not evaluate their networks further. The output node propensities for ISC, EB, and EC were found to be comparable with values from the FlyGut*-seq* database (28) (**Figure 3** and **Supplementary Table 9**). The full names of nodes in the network are mentioned in **Supplementary Table 10**.

### Case Study 1 – Investigating Colorectal Tumorigenesis Under Progressive Mutations in *Drosophila* Midgut

To decode the emergent cell fates during initiation and progression of colorectal cancer (CRC) in the adult *Drosophila* midgut, two salient driver mutations (55) in adenomatous polyposis coli (Apc, in WNT pathway) (74) and Ras (in the EGFR pathway) (75) were incorporated into the ISC network. These mutations were initially incorporated to act individually and later simultaneously (**Supplementary Figure 20**). The emergent cell fates in the control case (without mutations) included apoptosis, proliferation, and differentiation, along with loss of polarity, multi-layering, and extrusion with propensities of 0.296, 0.130, 0.130, 0.00, 0.077, and 0.179, respectively. Upon incorporation of Apc mutation into the ISC network, a slight decrease in apoptosis and proliferation was observed as their propensities decreased to 0.256 and 0.112, respectively. Differentiation and extrusion also got reduced to 0.112 and 0.151, respectively, while multi-layering increased to 0.256, and loss of polarity remained unaffected. Next, upon introducing Ras mutation, a decrease in apoptosis (0.210) and an increase in proliferation (0.148) was observed, which indicated cellular overgrowth. Furthermore, in line with Martorell et al. (55), loss of polarity and extrusion increased to 0.080 and 0.210, respectively.

**Figure 4:**
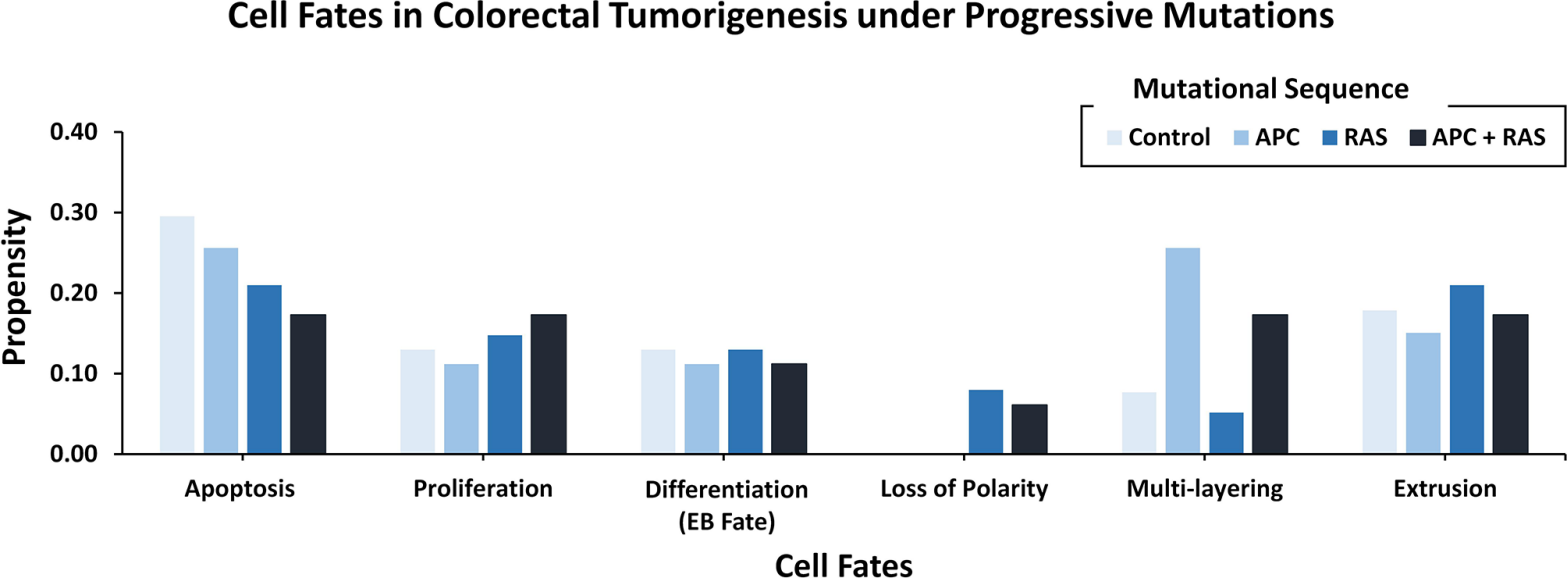
Cell fate outcomes after the introduction of progressive CRC mutations and their validation against Martorell et al.‘s Drosophila CRC model. A high rate of extrusion and loss of polarity was observed in Apc-Ras as well as Ras clones. Alongside, an increased proliferation rate with a decreased apoptosis and differentiation is also highlighted by Martorell et al. in their in *vivo* model.

On the other hand, the concurrent incorporation of Apc and Ras mutations resulted in hyper-proliferation and overgrowth as apoptosis decreased to 0.173 and proliferation increased to 0.173. The differentiation rate was observed to be 0.112 and loss of polarity, multi-layering and extrusion increased to 0.061, 0.173, and 0.173, respectively. Hence, with concurrent mutations in Apc and Ras, the emergent cell fates started exhibiting the hallmarks of cancer including abnormal proliferation and loss of differentiation, etc. (76). These results were also coherent with both the experimental findings reported by Martorell et al. (55) (**Figure 4** and **Supplementary Table 11**) and differential gene expression data (**Supplementary Table 12**).

### Case Study 2 – Therapeutic Evaluation of Raf-Mutation in *Drosophila* Midgut Using Targets From the Literature

Introduction of gain-of-function Raf-specific driver mutations in our ISC network enabled the replication of Markstein et al.’s (56) therapeutic screen towards a comparative cancer recurrence evaluation of nine FDA-approved drugs. In their gain-of-function Raf tumor model, Markstein and colleagues had classified FDA-approved drugs into class I and II drugs. According to the study class, I drugs induced cancer reversal in mutated cells without affecting the wild-type cells, in contrast, class II drugs induced cancerous phenotype in wild-type cells (**Supplementary Table 13**). The result of our network analysis of the control case exhibited proliferation and apoptosis with propensities of 0.157 and 0.286, respectively. However, after the induction of Raf mutations, the proliferation (0.162) rate increased along with a decrease in apoptosis (0.175). Treatment of a Raf-mutated network using class I drugs led to a decrease in proliferation (0.089) and an increase in apoptosis (0.263). For the wild type in comparison with the control, almost no effect was observed on apoptosis, which remained steady at 0.283 whereas a slight decrease was observed in proliferation (0.130). This confirmed the action of class I drugs which act to substantially reduce cancerous fates in cancer without having a major impact on wild-type cells.

**Figure 5:**
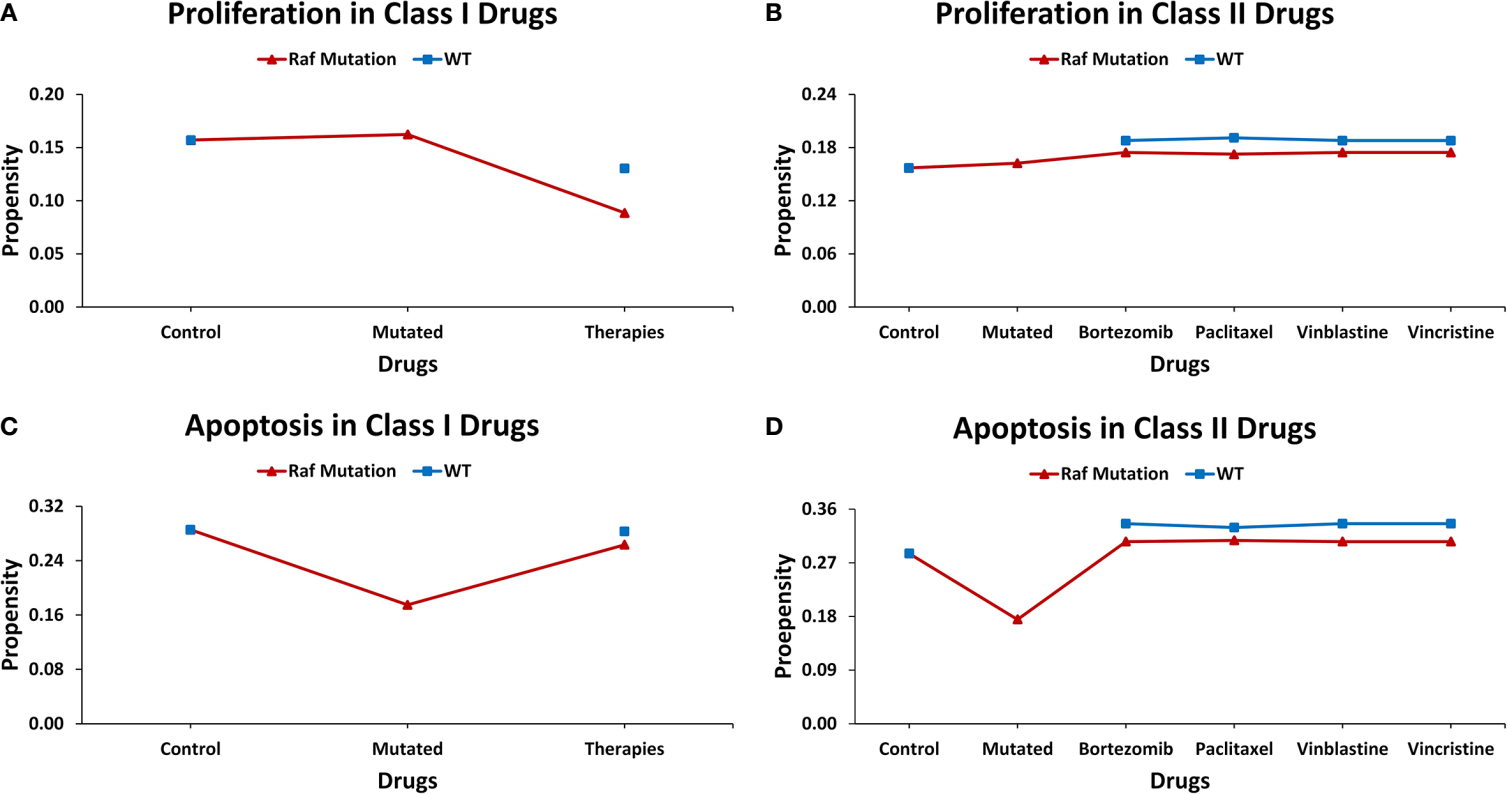
Evaluating cell fates under therapeutic screens taken from Markstein et al.‘s *Drosophila* model. **(A)** The effect of class I drugs on cell proliferation in wild type and mutated networks, **(B)** The effect of class II drugs on cell proliferation in wild type and mutated networks, **(C)** The effect of class I drugs on apoptosis in wild type and mutated networks, **(D)** The effect of class II drugs on apoptosis in wild type and mutated network.

Alternatively, in the case of class II drugs, the wild type also exhibited hyper-proliferation after therapy with its propensity reaching up to 0.191, and apoptosis increased to 0.336. Importantly, for the mutated network, drug action continued its activities with the propensity of proliferation reaching 0.175 and apoptosis at 0.306. These results suggest that class II drugs are indeed associated with drug cytotoxicity as they induced malignancy in normal cells under therapy. This confirms Markstein et al.’s study which hypothesized that the extracellular environment in animal models is crucial in drug delivery and cytotoxicity (**Figure 5** and **Supplementary Table 14**).

### Case Study 3 – Employing the *In Silico Drosophila Patient Model* (DPM) for Personalized Therapeutics

**Figure 6:**
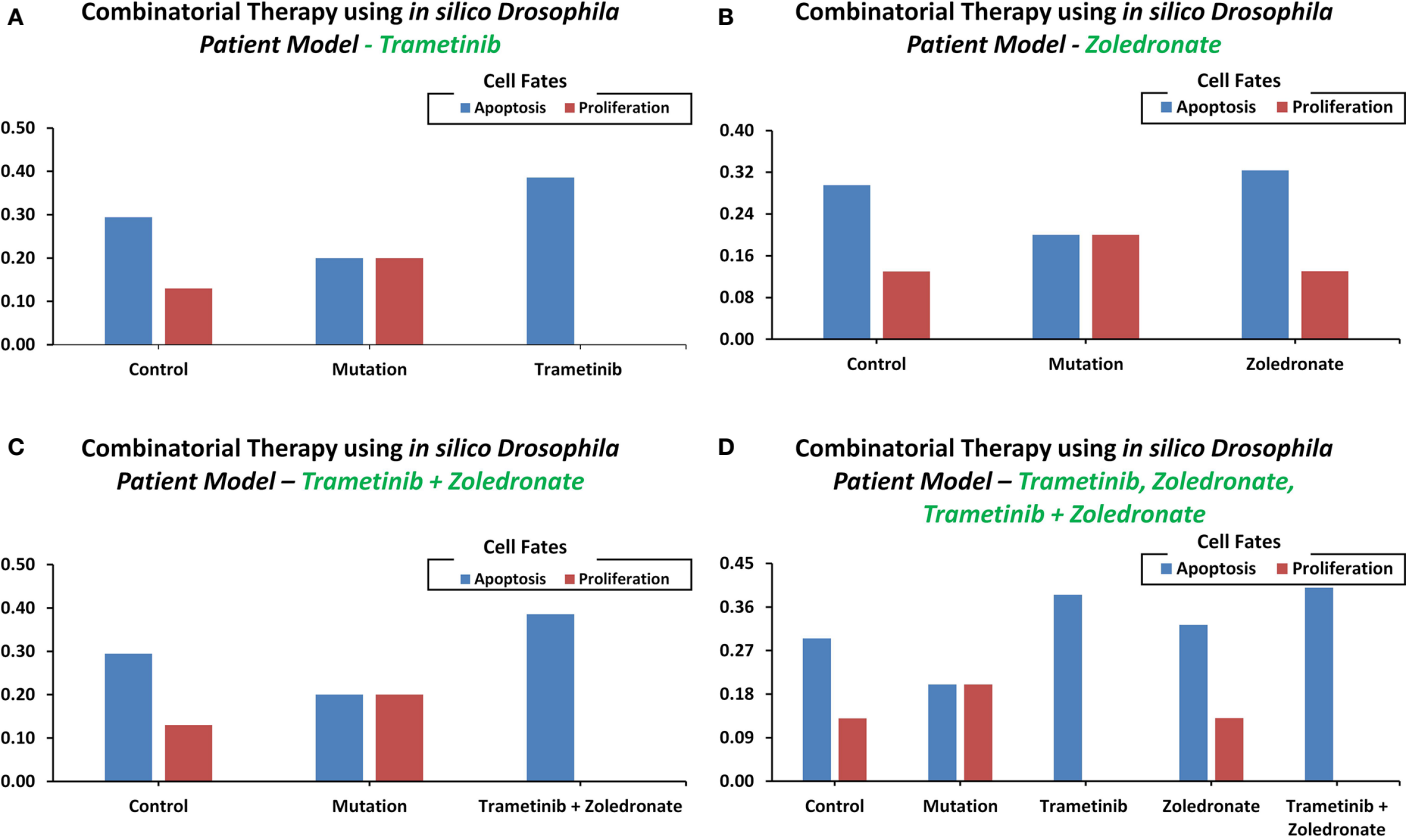
Cell fate propensities were obtained from the *in vivo Drosophila Patient Model* using Bangi et al.'s study. Cell fate propensities under **(A)** control, mutated, and therapy (Trametinib), **(B)** control, mutated, and therapy (Zoledronate), **(C)** control, mutated, and therapy (Trametinib + Zoledronate), **(D)** control, mutated, and therapy (Trametinib, Zoledronate, and Trametinib + Zoledronate).

Towards developing a *Drosophila-based* platform for employment in orchestrating patient-centric cancer therapeutics, we adopted Bangi et al.’s (40) *in vivo Drosophila Patient Model* (DPM). The *in vivo* model was first translated into an *in silico* DPM which incorporated patient-specific mutations from Bangi et al.’s study. These mutations included eight tumor suppressors: Apc, Tp53, Fbxw7, Tgfbr2, Smarca4, Fat4, Mapk14, and Cdh1, along with one oncogenic mutation in Kras (**Supplementary Table 15**). After inducing these patient-specific mutations into the ISC network (through direct and indirect target identification), we administered trametinib and zoledronate in different combinations to observe the most efficacious therapeutic effect. Our results showed that in control (i.e. healthy cells), the cell fate propensities for proliferation and apoptosis came out to be 0.130 and 0.294, respectively. Upon induction of mutations, proliferation increased to 0.200 and apoptosis decreased to 0.200, respectively. Next, with the administration of trametinib, an inhibitor of MEK kinase (mitogen-activated protein kinase kinase), used to treat patients with Kras mutation, the propensities for proliferation decreased to 0.000, whereas apoptosis increased to 0.386 (**Figure 6A**). With the administration of zoledronate, the cell fate propensities came out to be 0.130 for proliferation and 0.324 in the case of apoptosis (**Figure 6B**). Next, with the induction of zoledronate in combination with trametinib, a decrease in proliferation to 0.000 and an increase in apoptosis to 0.386 was observed (**Figure 6C**). Interestingly, augmentation of therapy with in tandem administration of trametinib, zoledronate, and trametinib with zoledronate showed proliferation to decrease to 0.000 and apoptosis to increase to 0.400 propensities (**Figure 6D**). These results exhibited cancer reversion on the administration of the drug combination and corroborate with Bangi et al.’s findings.

### Identification and Evaluation of Personalized Therapeutics for CRC Patients Using *In Silico* DPM

Towards developing personalized combinatorial therapies for treating colorectal cancer patients, we coupled our *in silico* DPM with patient-specific gene expression data from cBioPortal (16) (**Supplementary Table 16**). Patient-specific potential druggable targets were identified (from the 48 nodes in the ISC network) and their oncogenic cell fate (“apoptosis” and “proliferation” rates) propensities were obtained using the DA pipeline (**Supplementary Table 17**). Next, we employed PanDrugs (77) - an online database that prioritizes direct and indirect targeting of genomic mutations, to search for “*druggable genes*” in our networks. Each node was then queried in the database to find out the drugs that targeted them directly or indirectly (**Supplementary Tables 18, 19**). The results from this exercise elicited chemotherapy (paclitaxel/docetaxel) and targeted therapies (regorafenib, bortezomib, imatinib) depending on patient-specific mutations (**Supplementary Table 20**). Follow up literature review showed that these drugs and their combinations are currently being used in several studies and clinical trials (78–86). Specifically, the combination of the paclitaxel-regorafenib was evaluated for treating advanced esophagogastric cancer (78), and the paclitaxel-bortezomib combination was used in metastatic solid tumors (87). While the docetaxel-bortezomib combination was evaluated for metastatic breast cancer (79), Non-Small Cell Lung Cancer (NSCLC) (80, 81), and prostate cancer (82). Paclitaxel-imatinib combination was tested in metastatic solid tumors (83), NSCLC (84), and ovarian cancer (85).

**Figure 7:**
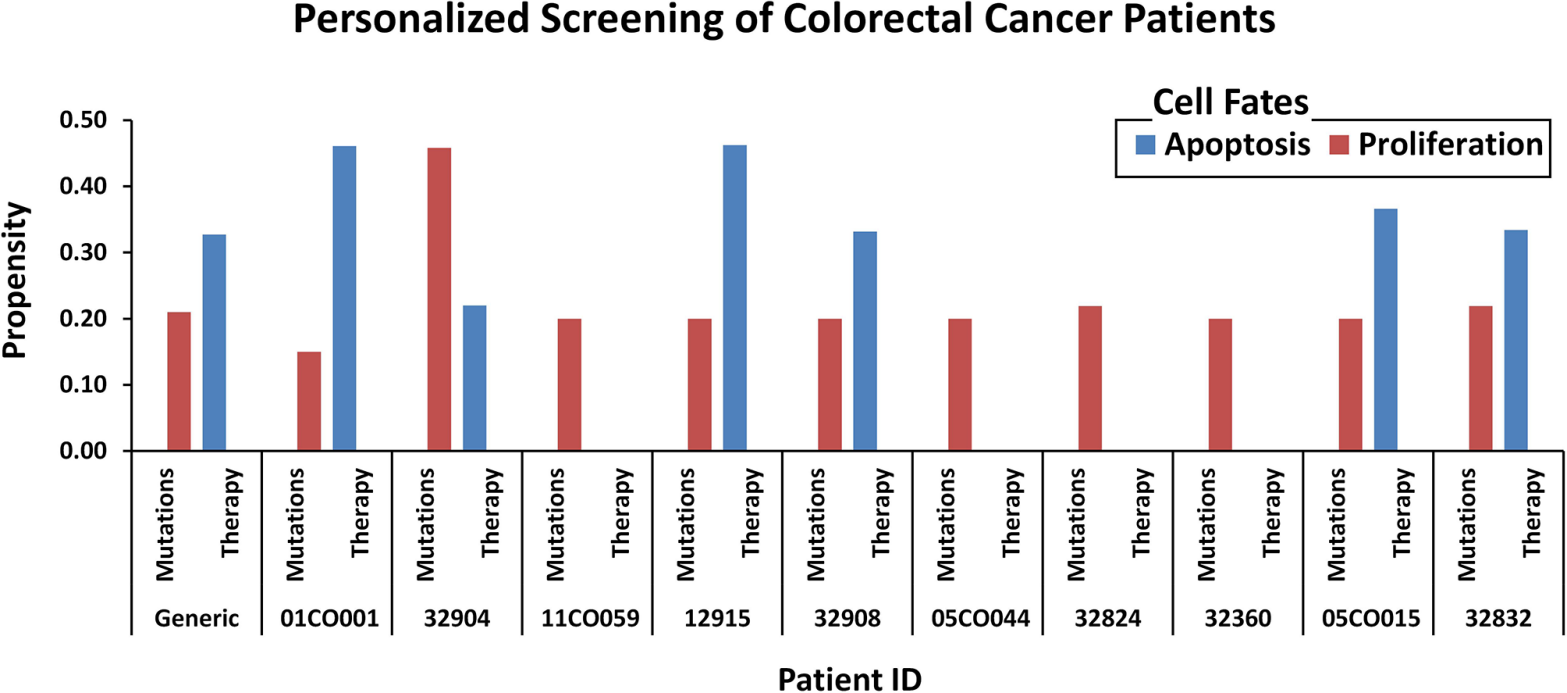
Comparison of oncogenic cell fate propensities obtained from personalized screening. Personalized screening of ten colorectal cancer patients. Patient ID and mutation data were extracted from cBioPortal and cell fates for apoptosis and proliferation were plotted to observe before and after therapy results.

To test the efficacy of these drug combinations in CRC patients, we administered these therapies using the proposed *in silico* DPMs to ten patients with colorectal adenocarcinoma obtained from cBioPortal (16). To implement the simultaneous action of chemotherapy wherein the drug introduces widespread inhibition of mitosis by stabilizing polymerized microtubules and not allowing them to function during cell division for that, we surveyed the existing literature on microtubule targeting (**Supplementary Table 21**, **Supplementary Figure 21**) and embedded it into ISC network (**Supplementary Table 22**) to study the behavior of microtubule stabilization-induced cell fates in chemotherapy. The resultant network consists of 54 nodes and 83 edges (**Supplementary Figure 22**). Our results from combinatorial chemo- and targeted therapy using an extended network showed up to a 100% increase in apoptosis cell fate and a 100% decrease in proliferation rate (**Figure 7** and **Supplementary Table 23**).

## Materials and Methods

The following sub-sections provide details of the methodology employed at each step of the study. The overall workflow of the study is outlined in **Supplementary Figure 23**.

### Data Collection and Boolean Modeling of Five Cell-Type-Specific Networks in *Drosophila* Midgut

To construct the biomolecular network models involved in the cellular regulation of *Drosophila* midgut, a comprehensive review of the existing literature and databases was undertaken. The databases employed included the Kyoto Encyclopedia of Genes and Genomes (KEGG) (88), Drosophila Interactions Database (DroID) (89), and data repositories such as FlyGut*-seq* (28). Alongside, network models of *Drosophila* by Giot et al. (90), Formstecher et al. (91), and Toku et al. were used to construct five rule-based Boolean biomolecular networks of the conserved signaling pathways in intestinal stem cells (ISCs) (43–47), enteroblasts (EBs) (48), enterocytes (ECs), enteroendocrine cells (EEs) (49–53), and visceral muscle (VM) cells (54). Nine major pathways involved in maintaining the overall homeostatic nature of the fly midgut were selected from the available literature. These included Notch (92), BMP (92), EGFR (93), WNT (94), JAK-STAT (94, 95), JNK (96), HIPPO (97), Insulin (63), and/or Robo (98) pathways for each cell type lining the midgut. The network steady states were used to program cell fate outcomes such as cellular differentiation, proliferation, apoptosis, EC fate determination, etc. Boolean equations (59) were used to model the regulation of each node in the biomolecular network. TISON (99), an in-house theatre for *in silico* systems oncology was used to translate Boolean rules into network models (see **Supplementary Data**, **Supplementary Table 24** for video tutorial).

### Robustness Analysis

To validate the biological plausibility of the proposed networks, a robustness analysis was performed (see **Supplementary Table 24** for video tutorial). Physiological conditions were maintained during this process and the input node values were taken from the FlyGut*-seq* database (28). The *normal* node states for ISC, EB, EC, and VM were perturbed by ±10%. Bootstrapping was employed on 10,000 network states. The means and standard deviations of the emergent cell fates were then calculated and the standard error of means (SEM) was plotted for each cell fate to determine the biological plausibility of the scale-free networks (100) (see **Supplementary Data 1**).

### Deterministic Analysis

The Boolean networks have been analyzed using the Deterministic Analysis (DA) (59) pipeline reported in ATLANTIS (101) and TISON (99) (see **Supplementary Table 24** for video tutorial). The results from DA were used to program “cell fate attractors” which are biological states that a cell can take, along with computation of their propensities (probability of their occurrence). Three different input files are used in this process which includes (i) network file, (ii) fixed node states file, and (iii) cell fate classification file. The network file contains the Boolean rules defining the biomolecular networks. The fixed node states file contained fixed values for generating environmental conditions such as normal, stress, or cancer conditions. The cell fate classification file is used to map network states onto the biological cell fates in the light of particular cell fate markers (101) (**Supplementary Table 25**). For network analysis, the DA pipeline starts with a set of initial network states. To achieve a steady state, logical rules, and state transition functions are employed. Upon reaching a steady-state a cell fate attractor is formed. This attractor can represent a specific cell fate with a cell fate propensity or basin size ratio. Bootstrapping was employed on 10,000 network states. TISON’s *Therapeutics Editor* (TE) was used to undertake therapeutic evaluation on the network using the DA pipeline, with mutation and drug data integrated (see **Supplementary Table 24** for video tutorial). Fixed node states for *normal* conditions were obtained from the FlyGut-*seq* database while for cancer conditions, literature was surveyed to find out if the pathway is up or downregulated. For *stress*, abnormal values were abstracted by perturbing the stimuli from *normal* conditions (see **Supplementary Data 2**).

### Network Annotation Using Flygut-seq Database

Towards annotating networks with experimental values, the FlyGut*-seq* database was employed. For that, an RNA-seq dataset consisting of rpkm values was exported from the database. Data were extracted for the relevant genes present in our networks (ISC, EB, and EC) using their biological names (**Supplementary Table 10**). Expression data across the five regions of the midgut (i.e. R1, R2, R3, R4, and R5) (102) was normalized for each gene in specific cells. The normalized values were taken as *normal* input fixed node states for onward analyses. The normalized values were also used to compare the output node propensities from DA that was performed under *normal* input conditions (**Supplementary Table 9**, for details, see **Supplementary Data 2**).

### Cell Fate Data Collection for Case Studies and Their Validation

To validate and exemplify our network models, we used three literature-based case studies on colorectal tumorigenesis in *Drosophila* melanogaster. For case study 1, data including cell fates under Apc and Ras single and simultaneous mutations were obtained from Martorell et al.’s model (55). The differential gene expression screens and data were also obtained from Martorell et al. (see **Supplementary Data 3**). TISON’s TE was used to implement the mutations in our network using TE’s horizontal therapy pipeline. For case study 2, therapeutic screens including the existing list of FDA-approved drugs for targeting ISC in *Drosophila* were adapted from Markstein et al.’s (56) study. Existing databases on drugs and drug-gene interactions such as PharmacoDB (103), PanDrugs (77), OncoKB (104), and DGIdb (105), etc (106, 107) were then used to identify target nodes in our ISC network, which were also mentioned in Markstein et al.’s study. TE was employed to deliver drug data into the CRC mutated network using TE’s vertical therapy pipeline (see **Supplementary Data 4**). For case study 3, patient-specific mutations, along with combinatorial therapy drug candidates were taken from Bangi et al.’s (40) study. Drug databases were used to identify target nodes in the ISC network mentioned in Bangi et al.’s study. Drugs that did not have direct targets in the network were implemented indirectly using literature-based mechanisms (see **Supplementary Data 5**).

### Development of an *In Silico Drosophila Patient Model* (DPM) and Its Validation

Towards devising a novel drug combination for the treatment of colorectal tumorigenesis, we performed an exhaustive evaluation of each node in our ISC network using TISON’s TE. For that, we started with the sensitivity analysis of both tumor suppressor genes and oncogenes involved in CRC using data from existing databases and literature (55, 103, 106, 107) against patient-specific mutations taken from cBioPortal (16). The therapeutic screening was performed by upregulating the tumor suppressors and downregulating the oncogenes (**Supplementary Table 26**), to evaluate potential drug combination targets using the PanDrugs (77) database, a platform that prioritizes direct and indirect targeting of genomic mutations (see **Supplementary Data 6**).

### Combination of Chemotherapy and Targeted Therapy to Treat CRC Patients

To induce the effect of chemotherapy we carried an extensive survey of the existing literature and constructed a microtubule network. The microtubule network was incorporated by integrating 6 nodes and 13 interactions to the ISC network. The resultant network contained 54 nodes and 83 interactions. This integrated network was then utilized for chemotherapeutic screening. The combinatorial personalized therapy was used to treat the CRC patients, in a vertical therapy scheme through targeting specific nodes in our ISC network in light of patient-specific mutations. DA pipeline was used to carry out the therapeutic evaluation (see **Supplementary Data 6**).

## Discussion

Combinatorial therapies have created avenues for enhanced treatment of colorectal cancer (CRC) through drug synergy (108). Translational studies using omics-based data can help develop efficacious drug combinations for individualized CRC treatment. In particular, *in silico* Boolean models that utilize omics datasets can facilitate the process of developing and evaluating different drug combination therapies for the treatment of CRC (109–111). In this work, we propose a novel *in silico Drosophila Patient Model* (DPM), a computational framework for devising personalized therapeutic combinations for CRC patients. For that, we have constructed Boolean network models of five cell types present in *Drosophila* midgut: (i) intestinal stem cell, (ii) enteroblast, (iii) enterocyte, (iv) enteroendocrine, and (iv) visceral muscles (**Figures 1A–D**). We have used these networks to systematically induct tumorigenesis in *Drosophila* midgut tissue followed by therapeutic interventions for tumor reversion and restoration of physiological homeostasis (**Figure 2**). We then employed the ISC networks to create an *in silico* DPM for identifying optimal combinatorial therapeutics to treat CRC in humans. Our modeling pipeline provides a novel roadmap to annotate Boolean network models with patient data towards developing personalized medicine for CRC patients.

Several network models of biomolecular regulation in *Drosophila* have been reported for investigating the regulatory dynamics in cancer (90, 91, 112–114). Specifically, such applications of adult *Drosophila* midgut models are particularly useful in investigating CRC due to cellular and organizational similarities between *Drosophila* midgut and the human colon. More so, the biomolecular signaling pathways involved in maintaining homeostasis and differentiation are also conserved in both. This has given impetus to the development and utilization of *Drosophila* midgut models for investigating human colorectal cancer (115, 116). As a result, fly-based midgut models have been employed to investigate tissue homeostasis (117), multi-step tumorigenesis (55), epithelium renewal and regeneration upon bacterial infection or tissue damage (118), and its effect on mature and undifferentiated epithelial cells during intestinal cancer initiation (119). However, the employment of *Drosophila* midgut networks has hitherto remained unannotated with patient-specific mutation to study tumorigenesis in CRC thus limiting their translational potential. In this study, we have employed three literature-based case studies on *in vivo Drosophila* model to investigate CRC, employing *in silico* approaches. In our first case study, we used a fly-based network model to help investigate colorectal tumorigenesis under progressive mutations; the results from our analysis were validated against Martorell’s CRC model (55) (**Figure 4**). The results from our second case study helped elucidate cytotoxicity in nine FDA-approved drugs (**Figure 5**) and conformed with Markstein et al.’s (56) hypothesis that the extracellular environment plays a crucial role in animal models for evaluating drug delivery and cytotoxicity. Next, for the third case study, we used Bangi et al.’s *in vivo* DPM to perform personalized therapy for KRAS-mutant metastatic colorectal cancer patient (40) (**Figure 6**), which re-confirmed the potential of combinatorial treatment; trametinib, zoledronate followed by trametinib in combination with zoledronate.

Onwards, we have performed personalized therapeutics by incorporating patient-specific mutation data into our model towards devising novel combinatorial treatments. For that, we took patient-specific data on ten patients with colorectal adenocarcinoma obtained from cBioPortal (16) to annotate our network model (**Supplementary Table 16**). We then undertook an exhaustive screening towards identifying efficacious target nodes for each patient which was based on the node’s pro-apoptotic and anti-proliferation cell fate propensities after therapy (**Supplementary Table 17**). We used the PanDrugs database (77) to identify these target nodes in existing drugs. In light of our personalized screening step, we discovered that four patients can respond well to targeted therapy (imatinib, regorafenib, and everolimus), whereas for the rest a synergistic combination of chemotherapy (paclitaxel/docetaxel) and targeted therapy (imatinib, regorafenib, and bortezomib) was a more efficacious treatment (**Supplementary Table 20**). Literature also supports our finding that CRC treatment using a combination of chemo- and targeted therapy can provide efficacious results compared to conventional chemotherapy alone (119, 120). Specifically, the combination of the paclitaxel-regorafenib was evaluated for treating advanced esophagogastric cancer (78), and the paclitaxel-bortezomib combination was used in metastatic solid tumors (87). While the docetaxel-bortezomib combination was evaluated for metastatic breast cancer (79), Non-Small Cell Lung Cancer (NSCLC) (80, 81), and prostate cancer (82). Paclitaxel-imatinib combination was tested in metastatic solid tumor (83), NSCLC (84), and ovarian cancer (85, 86). However, further validation of these prognostic drug combinations in large-scale clinical cohorts will be required to test these drug combinations suggested by our study. In unison, our findings suggest that the proposed translational approach is effective in optimizing existing therapies.

Limitations of this study include utilizing abstracted *in silico* Boolean models (59) which are only qualitative. Moreover, analysis of EE and VM networks remained limited due to a lack of substantive literature. In this work, to overcome exponential computational complexity due to network size, we pruned each network to a minimum while maintaining biological cell fate outcomes. Additionally, ISC-EB-EC interplay is pivotal in determining cell fates, especially for intestinal stem cells in *Drosophila* midgut, however, due to network-level analysis strategy employed in the study, we are currently unable to investigate cellular interplays as well as continuous lineage tracking for various cell types. Since our networks are independent of each other we can only elucidate individual cell fates programmed by each network at a time.

Several assumptions have been made for constructing this model. Firstly, since *Drosophila* midgut comprises of several regions with differential niches and context specific cellular processes (42), for the sake of computational scalability, we have not incorporated *Drosophila* midgut compartmentalization in our model. In view of the exponential relation between computational complexity and network size, we have kept the network size to a minimum by reducing path lengths between critical nodes through removing intermediary nodes. Integrated multi-omics information e.g., from genomics, transcriptomics, and proteomics level was assumed to act on the same time-scale, towards undertaking network analysis.

With regards to drugs, we search the nodes (genes) in our network in PanDrugs database for selecting and prioritizing potential drugs that can efficaciously target the selected nodes. The assumptions made by PanDrugs for declaring a gene-drug relationship, include: for targeted therapies, the genes-drug relationship that PanDrugs provides is a direct relationship, and that the targeted drugs acting directly on the nodes in the network are without any off target pleotropic effects. PanDrugs’s drug prioritization scheme can improve if it also takes into account protein interaction networks, pathway activity, multi-omics information, however, its search is limited to genome-level information only. Moreover, each drug is able to act on all the possible transcriptomic isoforms of a gene, where necessary.

Additionally, during the personalized screening of patients, non-druggable nodes could not be evaluated further due to unsubstantive literature on their employment as drugs. Moreover, some of the genes present in the human genome do not have exact homologs in *Drosophila’s* genes list, which can limit the study’s translational capabilities.

Onwards, the proposed *in silico* DPM can be extended to perform probabilistic analysis by converting rules to the weights-based network which can also cater to external perturbations and noise into the system. Further investigations need to be carried out to predict novel druggable genes (direct targets, biomarkers, and pathway members not available in PanDrugs database) for employment in developing new drug combinations. The network models developed can also be extended to multi-scale models towards incorporating spatiotemporal regulations of colorectal cancer. Further verifications with a greater patient sample size can help achieve a better understanding of the relationship between patient-specific data in connection to therapeutic combinations. Moreover, result verification can be enhanced with wet lab validation of the proposed synergistic drug combinations outlined by our computational framework.

Taken together, our preclinical *in silico* DPM not only captures the regulatory homeostasis of fly midgut but also presents a novel framework to personalize Boolean network models towards their employment in personalized cancer therapeutic interventions.

## Data Availability Statement

The original contributions presented in the study are included in the article/**Supplementary Material**. Further inquiries can be directed to the corresponding author.

## Author Contributions

SC designed and supervised the study. MG carried out the literature review, construction of the model, and undertook the analyses. SC, MG, and RN designed the personalized treatment pipeline, SC, MG, RN, ZN, and HK drafted the manuscript. OS helped construct Boolean networks. RH critically reviewed the model development and performed validations, RQ, MT, and AF assisted in the study design and manuscript development. All authors contributed to the article and approved the submitted version.

## Funding

This work was supported by the National ICT-R&D Fund (SRG-209), RF-NCBC-015, NGIRI-2020-4771, HEC (21-30SRGP/R&D/HEC/2014, 20-2269/NRPU/R&D/HEC/12/4792 and 20-3629/NRPU/R&D/HEC/14/585), TWAS (RG 14-319 RG/ITC/AS_C) and LUMS (STG-BIO-1008, FIF-BIO-2052, FIF-BIO-0255, SRP-185-BIO, SRP-058-BIO and FIF-477-1819-BIO) grants.

## Conflict of Interest

The authors declare that the research was conducted in the absence of any commercial or financial relationships that could be construed as a potential conflict of interest.
