# Supplementary material for: A Personalized Therapeutics Approach Using an In Silico Drosophila Patient Model Reveals Optimal Chemo- and Targeted Therapy Combinations for Colorectal Cancer
Source: Front Oncol. 2021 Jul 16;11:692592. doi: 10.3389/fonc.2021.692592 (PMC8323493; doi:10.3389/fonc.2021.692592)
Supplement: Supplementary file 1 [file DataSheet_1.zip › Supplementary_Data.docx]

The supplementary data of manuscript titled “*A personalized therapeutics approach using an in silico Drosophila Patient Model reveals optimal chemo- and targeted therapy combinations for colorectal cancer*” including input files, output files and analysis results are available at GitHub on this URL: <https://github.com/BIRL/DrosophilaPatientModel>
