# Supplementary material for: A Personalized Therapeutics Approach Using an In Silico Drosophila Patient Model Reveals Optimal Chemo- and Targeted Therapy Combinations for Colorectal Cancer
Source: Front Oncol. 2021 Jul 16;11:692592. doi: 10.3389/fonc.2021.692592 (PMC8323493; doi:10.3389/fonc.2021.692592)
Supplement: Supplementary file 2 [file DataSheet_2.pdf]

Supplementary Material

A personalized therapeutics approach using an *in silico Drosophila Patient Model* reveals optimal chemo- and targeted therapy combinations for colorectal cancer

Mahnoor Naseer Gondal[1], Rida Nasir Butt[1], Osama Shiraz Shah[1], Muhammad Umer Sultan[1], Ghulam Mustafa[1], Zainab Nasir[1], Risham Hussain[1], Huma Khawar[1], Romena Qazi[2], Muhammad Tariq[3], Amir Faisal[4], Safee Ullah Chaudhary[1,*]

[1] Biomedical Informatics Research Laboratory, Department of Biology, Lahore University of Management Sciences, Lahore 54792, Pakistan

[2] Department of Pathology, Shaukat Khanum Memorial Cancer Hospital and Research Centre, Lahore, Pakistan

[3] Epigenetics Laboratory, Department of Biology, Lahore University of Management Sciences, Lahore 54792, Pakistan

[4] Cancer Therapeutics Laboratory, Department of Biology, Lahore University of Management Sciences, Lahore 54792, Pakistan

*** Correspondence:**
Safee Ullah Chaudhary
safee.ullah.chaudhary@gmail.com

Keywords: Personalized in silico cancer models; Boolean network models; Cancer systems biology; Preclinical in silico drug screening; Combinatorial therapeutic

Table of Contents

Table of Contents 2

List of Supplementary Tables 3

List of Supplementary Figures 5

1.1. Supplementary Figures 7

1.2. Supplementary Tables 28

1.3. References 112

List of Supplementary Tables

Supplementary Table 1: Detailed node interaction rules and experimental evidences supporting different interactions and logical functions for Intestinal Stem Cells (ISC) model. 28

Supplementary Table 2: Detailed node interaction rules and experimental evidences supporting different interactions and logical functions for Enteroblast (EB) model. 35

Supplementary Table 3: Detailed node interaction rules and experimental evidences supporting different interactions and logical functions for Enterocyte (EC) model. 41

Supplementary Table 4: Detailed node interaction rules and experimental evidences supporting different interactions and logical functions for Enteroendocrine (EE) model. 46

Supplementary Table 5: Detailed node interaction rules and experimental evidences supporting different interactions and logical functions for Visceral Muscle (VM) cells model. 51

Supplementary Table 6: Robustness analysis cell fates and corresponding SEMs for ISC, EB, EC and VM. 56

Supplementary Table 7: Input node states in normal, stress and cancer for ISC's Apical region, ISC's Basal region, EB and EC network models and their literature validation. 57

Supplementary Table 8: Cell fate propensities of Intestinal Stem Cells (ISC) in apical and basal compartments; Enteroblast (EB) and Enterocytes (EC) in normal, stress and cancer conditions along with literature validations.. 58

Supplementary Table 9: A comparison of model and experimental output node propensities. ... 59

Supplementary Table 10: Tabulation of network nodes, gene IDs, annotation symbols, gene symbols, and FlyBase genes. 60

Supplementary Table 11: Martorell et al.'s predictions: experiment versus model. 64

Supplementary Table 12: Differential gene expression comparison between prediction and the model.. 65

Supplementary Table 13: Results of class I and class II drugs from Markstein *et al*.'s therapeutics screens. 68

Supplementary Table 14: Cell fate propensities for proliferation and apoptosis in class I and class II drugs. 70

Supplementary Table 15: Details of the Bangi et al.'s case study: mutations, therapy, and induction of therapy in the in silico DPM. 71

Supplementary Table 16: Patient mutations from cBioPortal for genes existing in our network 72

Supplementary Table 17: Oncogenic cell fate propensities of potential target nodes, highlighted in blue (pro-apoptotic) and green (anti-proliferation)... 74
Supplementary Table 18: Personalized therapeutic combinations for individual patients against genes in PanDrugs database.. 78
Supplementary Table 19: Potential efficacious nodes queried in PanDrugs database. 89
Supplementary Table 20: Personalized therapeutic combinations for individual patients.......... 91
Supplementary Table 21: Detailed node interaction rules and experimental evidences supporting different interactions and logical functions Microtubule model regulations............................... 92
Supplementary Table 22: Detailed node interaction rules and experimental evidences supporting different interactions and logical functions for Integrated (ISC+MT) model.............................. 97
Supplementary Table 23: Results from targeted therapy of colorectal cancer patients............. 106
Supplementary Table 24: Tutorial for performing analysis for the study. 107
Supplementary Table 25: Mapping of cell fate classification logic 108
Supplementary Table 26: Details of tumor suppressors and oncogenes in ISC network.......... 109

List of Supplementary Figures

Supplementary Figure 1: Anatomical outlay and regulatory mechanisms for inducing tumorigenesis in the digestive tract of Drosophila melanogaster. 7
Supplementary Figure 2: Schematic representation of regulation in intestinal stem cells. 8
Supplementary Figure 3: Schematic representation of regulation in enteroblast. 8
Supplementary Figure 4: Schematic representation of regulation in enterocyte. 9
Supplementary Figure 5: Schematic representation of regulation in enteroendocrine. 10
Supplementary Figure 6: Schematic representation of regulation in visceral muscle. 10
Supplementary Figure 7: Summary Standard Error of Means (SEM) for ISC, EB, EC and VM. 11
Supplementary Figure 8: Intestinal stem cell in apical region, cell fate outcomes in normal condition 12
Supplementary Figure 9: Intestinal stem cell in apical region, cell fate outcomes in stress condition 13
Supplementary Figure 10: Intestinal stem cell in apical region, cell fate outcomes in cancer condition 14
Supplementary Figure 11: Intestinal stem cell in basal region, cell fate outcomes in normal condition 15
Supplementary Figure 12: Intestinal stem cell in basal region, cell fate outcomes in stress condition 16
Supplementary Figure 13: Intestinal stem cell in basal region, cell fate outcomes in cancer condition 17
Supplementary Figure 14: Enteroblast, cell fate outcomes in normal condition.......................... 18
Supplementary Figure 15: Enteroblast, cell fate outcomes in stress condition 19
Supplementary Figure 16: Enteroblast, cell fate outcomes in cancer condition........................... 20
Supplementary Figure 17: Enterocyte, cell fate outcomes in normal condition........................... 21
Supplementary Figure 18: Enterocyte, cell fate outcomes in stress condition 22
Supplementary Figure 19: Enterocyte, cell fate outcomes in cancer condition............................ 23
Supplementary Figure 20: Schematic of homeostasis, differentiation and tumorigenesis in normal and diseased midgut. 24
Supplementary Figure 21: Schematic representation of regulation in microtubule. 25

Supplementary Figure 22: Schematic representation of regulation in intestinal stem cells and microtubule network. 26
Supplementary Figure 23: Workflow outlines our study steps.. 27

1.1. Supplementary Figures

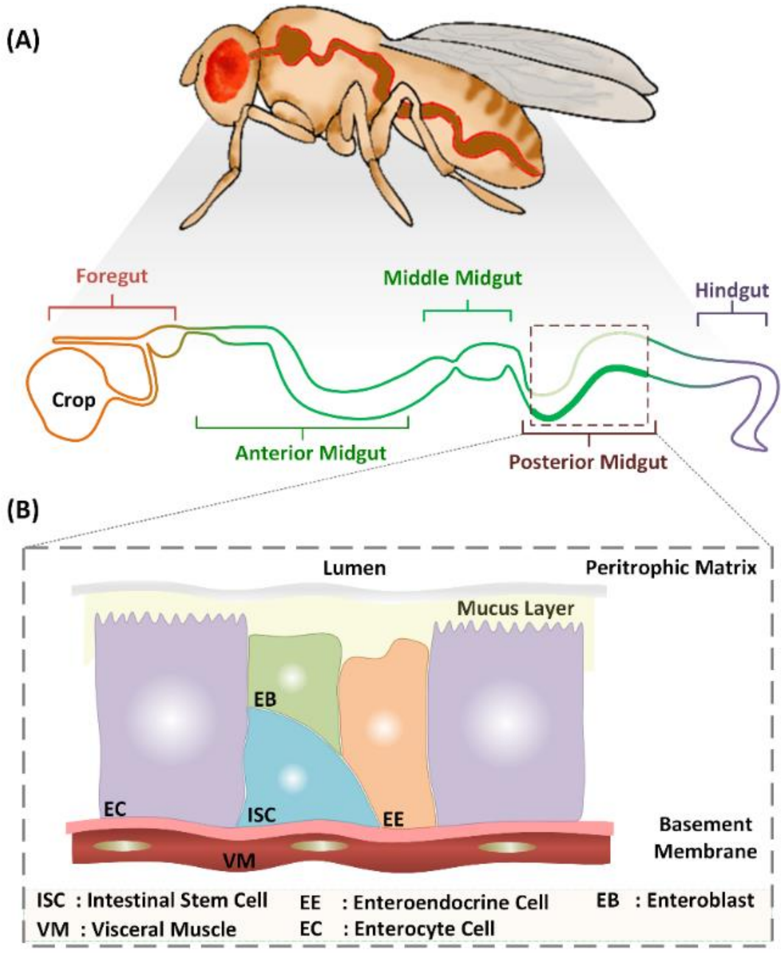

Supplementary Figure 1: Anatomical outlay and regulatory mechanisms for inducing tumorigenesis in the digestive tract of *Drosophila* melanogaster.
(A) Schematic organization of *Drosophila* gut and its subdivision into crop, foregut, midgut and hindgut **(B)** Epithelial layer of cells in midgut supported by an underlying basal membrane. This includes the gut lumen, mucus layer, peritrophic matrix, basement membrane and five major types of midgut cells. The lumen holds the food bolus, peritrophic matrix provides protection from mechanical and chemical damage by acting as a barrier between the cellular structures within epithelial membrane and the lumen environment, and basement membrane provides support to epithelial cellular structures. Note that the midgut epithelium is a niche to various kinds of cells including Intestinal Stem cells (ISCs), Enteroblast (EB), Enterocytes (EC), and Enteroendocrine (EE) amongst other progenitor cells, which enable its digestive and absorptive function. Most abundant of these cells are the ECs (1,2).

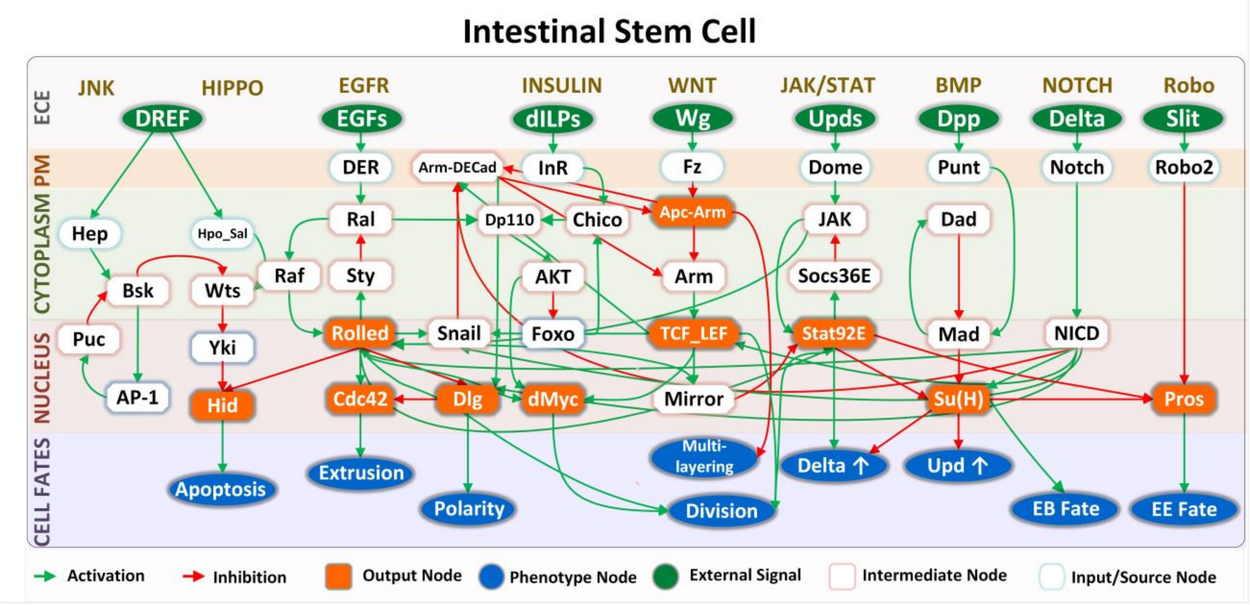

Supplementary Figure 2: Schematic representation of regulation in intestinal stem cells. Intestinal Stem Cell (ISC) in both apical and basal regions employ nine major signaling pathways including EGFR, WNT, JAK/STAT, BMP, NOTCH, JNK, HIPPO, Insulin, and Robo to maintain homeostasis and regeneration in the midgut. The inputs (green boxes) to these pathways are EGFs, Wg, Upds, Dpp, Delta, DREF, dILPs, and Slit, respectively. Each input is mapped to the output through an intermediate layer of nodes. The outputs (orange boxes) include Rolled, Cdc42, Hid, Dlg, Apc-Arm, TCF-LEF, STAT92E, Su(H), dMyc, and Pros. The output layer is used to program cell fates which includes Extrusion, Apoptosis, Polarity, Division, Multilayering, Delta and Upd Production, EB Fate and EE Fates.

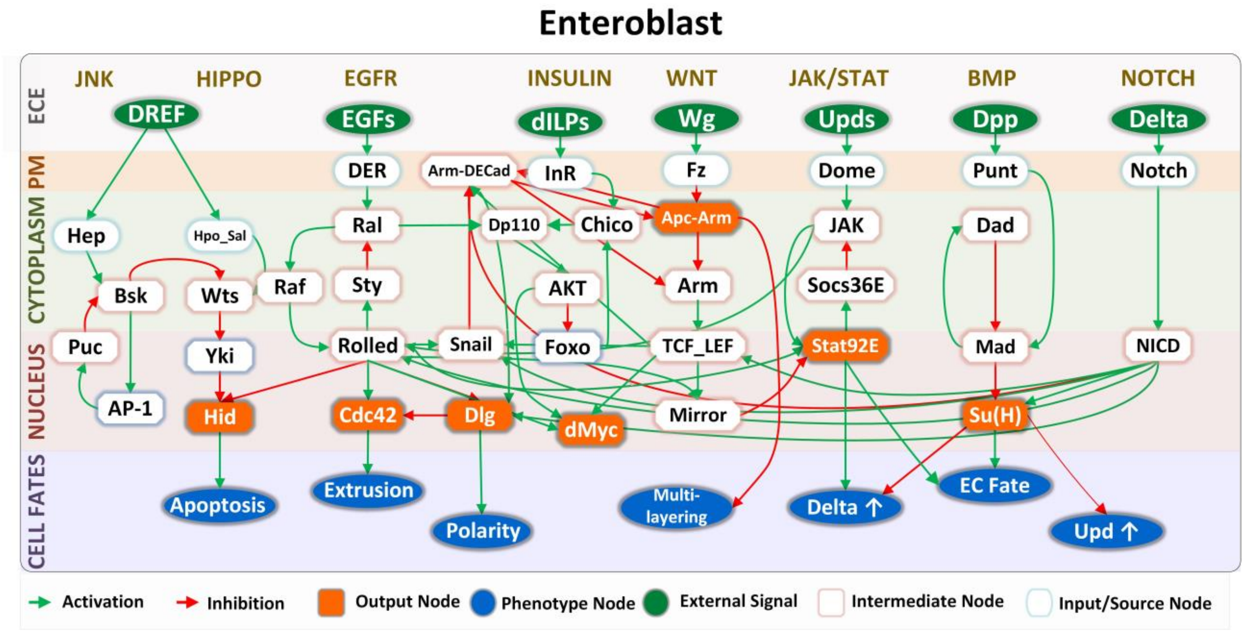

Supplementary Figure 3: Schematic representation of regulation in enteroblast.

Enteroblasts employ five major signaling pathways including EGFR, WNT, JAK/STAT, BMP, JNK, HIPPO, Insulin, and NOTCH. The inputs (green boxes) to these pathways are EGFs, Wg, Upds, Dpp, DREF, dILPs, and Delta, respectively. Each input is mapped on to the output through an intermediate layer of nodes. The outputs (orange boxes) include Cdc42, Hid, Dlg, Apc-Arm, STAT92E, dMyc, and Su(H). The output layer is used to program cell fates which include Extrusion, Apoptosis, Polarity, Multilayering, Delta and Upd Production and EC Fate.

Enterocyte

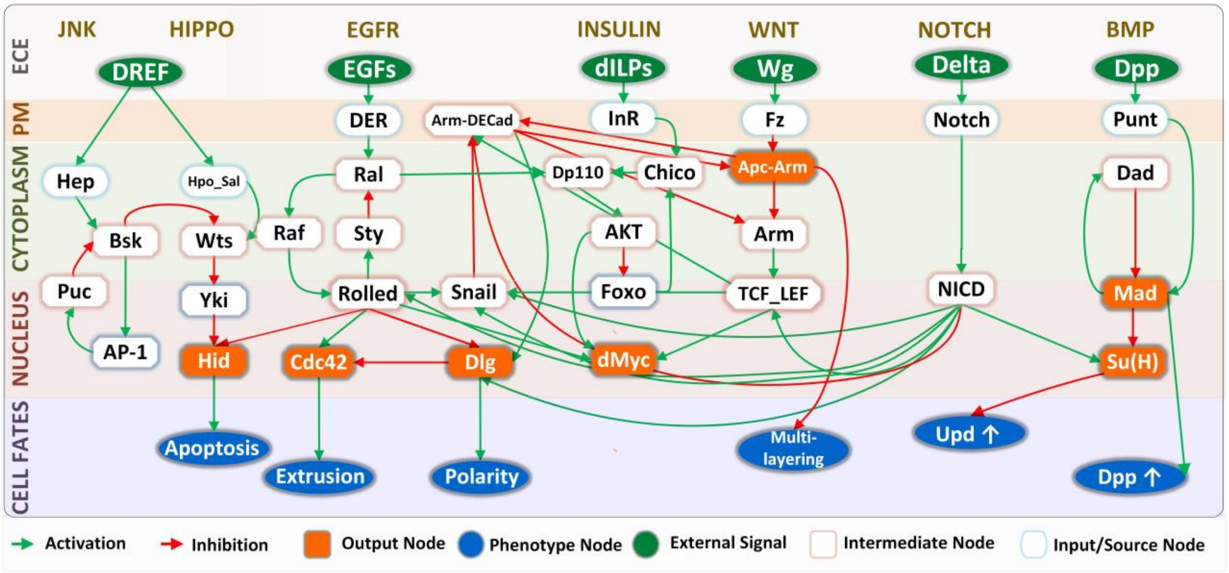

Supplementary Figure 4: Schematic representation of regulation in enterocyte. Enterocytes employ four major signaling pathways including EGFR, WNT, BMP, JNK, HIPPO, Insulin, and NOTCH. The inputs (green boxes) to these pathways are EGFs, Wg, Dpp, DREF, dILPs and Delta, respectively. Each input is mapped to the output through an intermediate layer of nodes. The outputs (orange boxes) include Cdc42, Hid, Dlg, Apc-Arm, dMyc, and Su(H). The output layer is used to program cell fates which include Extrusion, Apoptosis, Polarity, Multilayering, Dpp and Upd Production.

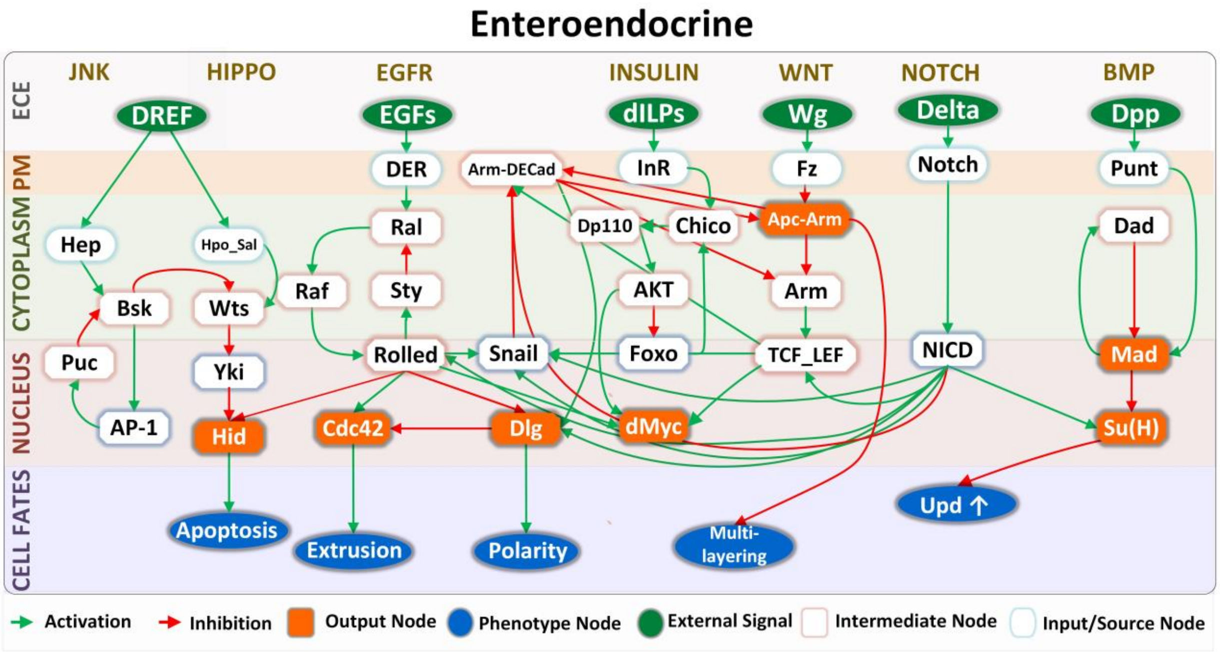

Supplementary Figure 5: Schematic representation of regulation in enteroendocrine. Enteroendocrine cells employ four major signaling pathways including EGFR, WNT, BMP, JNK, HIPPO, Insulin and NOTCH. The input (green boxes) to these pathways are EGFs, Wg, Dpp, DREF, dILPs, and Delta, respectively. Each input is mapped to the output through an intermediate layer of nodes. The outputs (orange boxes) include Cdc42, Hid, Dlg, Apc-Arm, dMyc, and Su(H). The output layer is used to program cell fates which include Extrusion, Apoptosis, Polarity, Multilayering, and Upd Production.

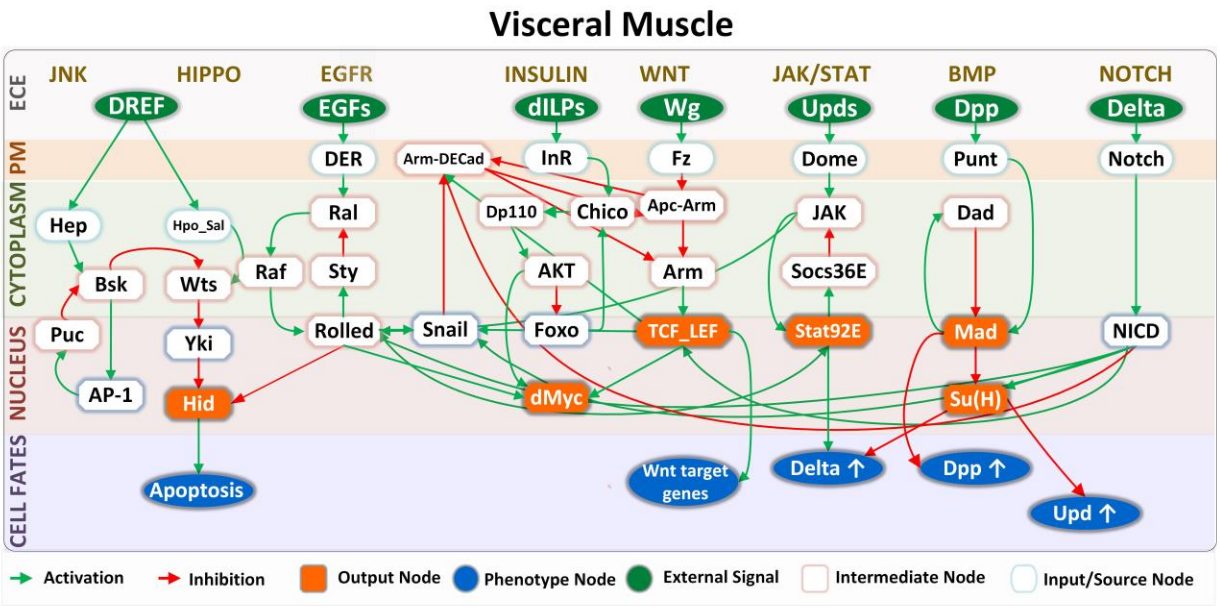

Supplementary Figure 6: Schematic representation of regulation in visceral muscle. Visceral Muscle cells employ five major signaling pathways including EGFR, WNT, JAK/STAT, BMP, JNK, HIPPO, Insulin, and NOTCH. The inputs (green boxes) to these

pathways are EGFs, Wg, Upds, Dpp, DREF, dILPs, and Delta, respectively. Each input is mapped on to outputs through an intermediate layer of nodes. The outputs (orange boxes) include Cdc42, Hid, Dlg, Apc-Arm, STAT92E, dMyc, and Su(H). The output layer is used to program cell fates which include Apoptosis, Wnt target genes, Delta, Upd and Dpp Production.

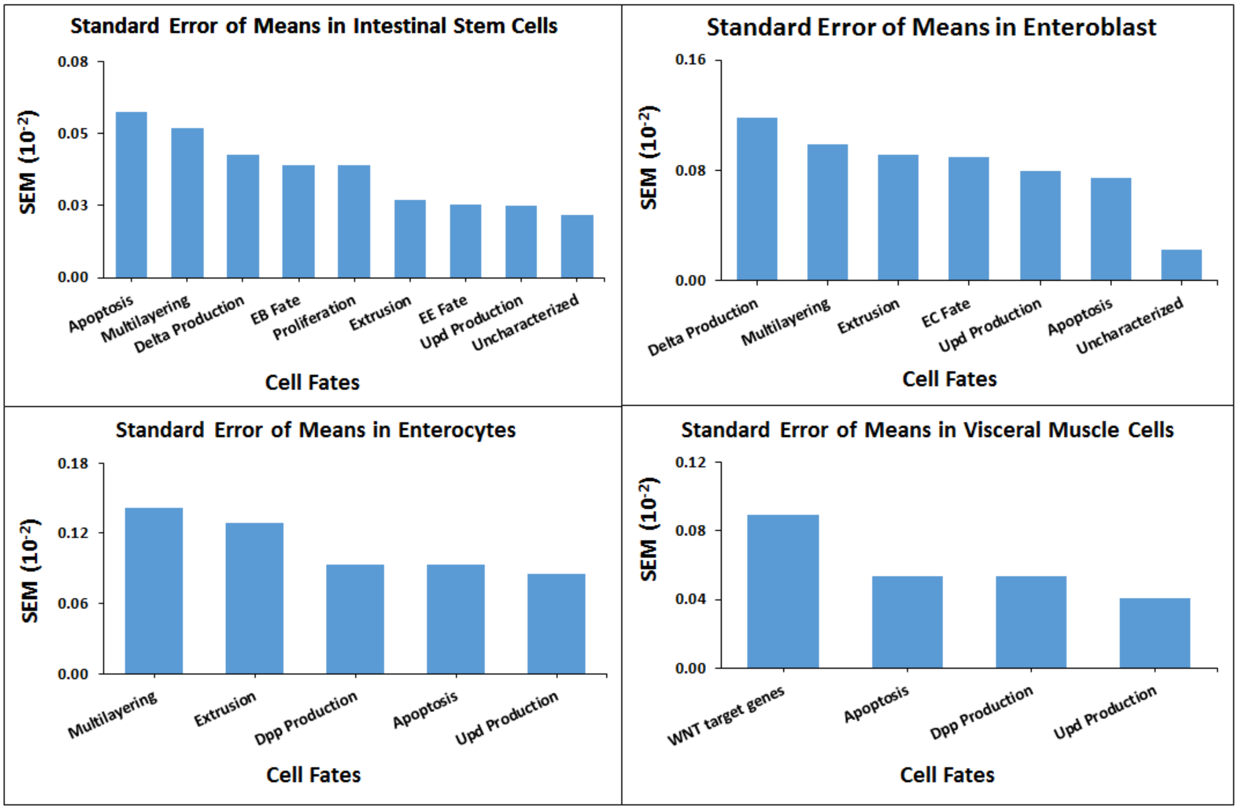

Supplementary Figure 7: Summary Standard Error of Means (SEM) for ISC, EB, EC and VM.

The SEM with highest for Apoptosis in ISC (0.0006), Delta production for EB (0.0012), Multi-layering for EC (0.0014) and WNT target gene fate for VM (0.0009).

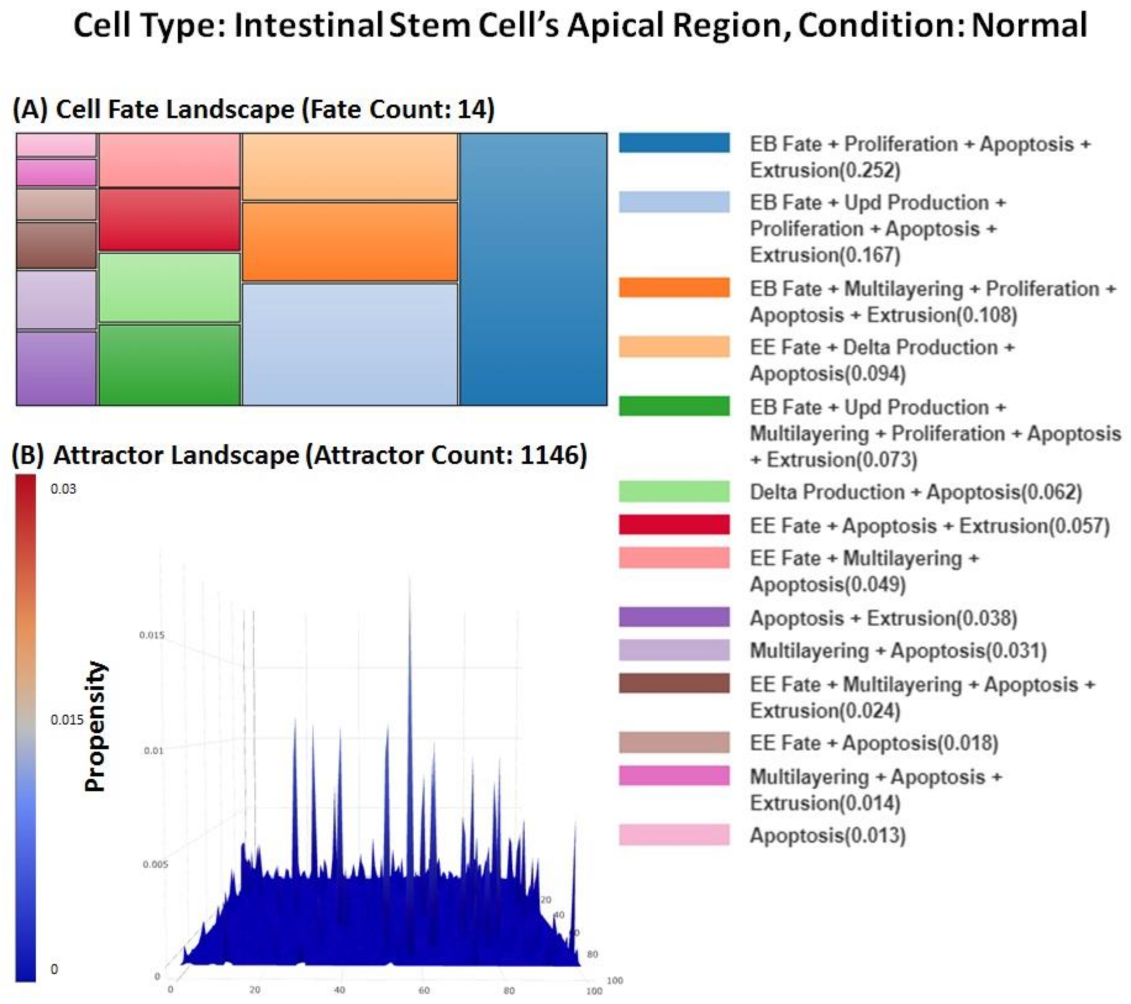

Supplementary Figure 8: Intestinal stem cell in apical region, cell fate outcomes in normal condition

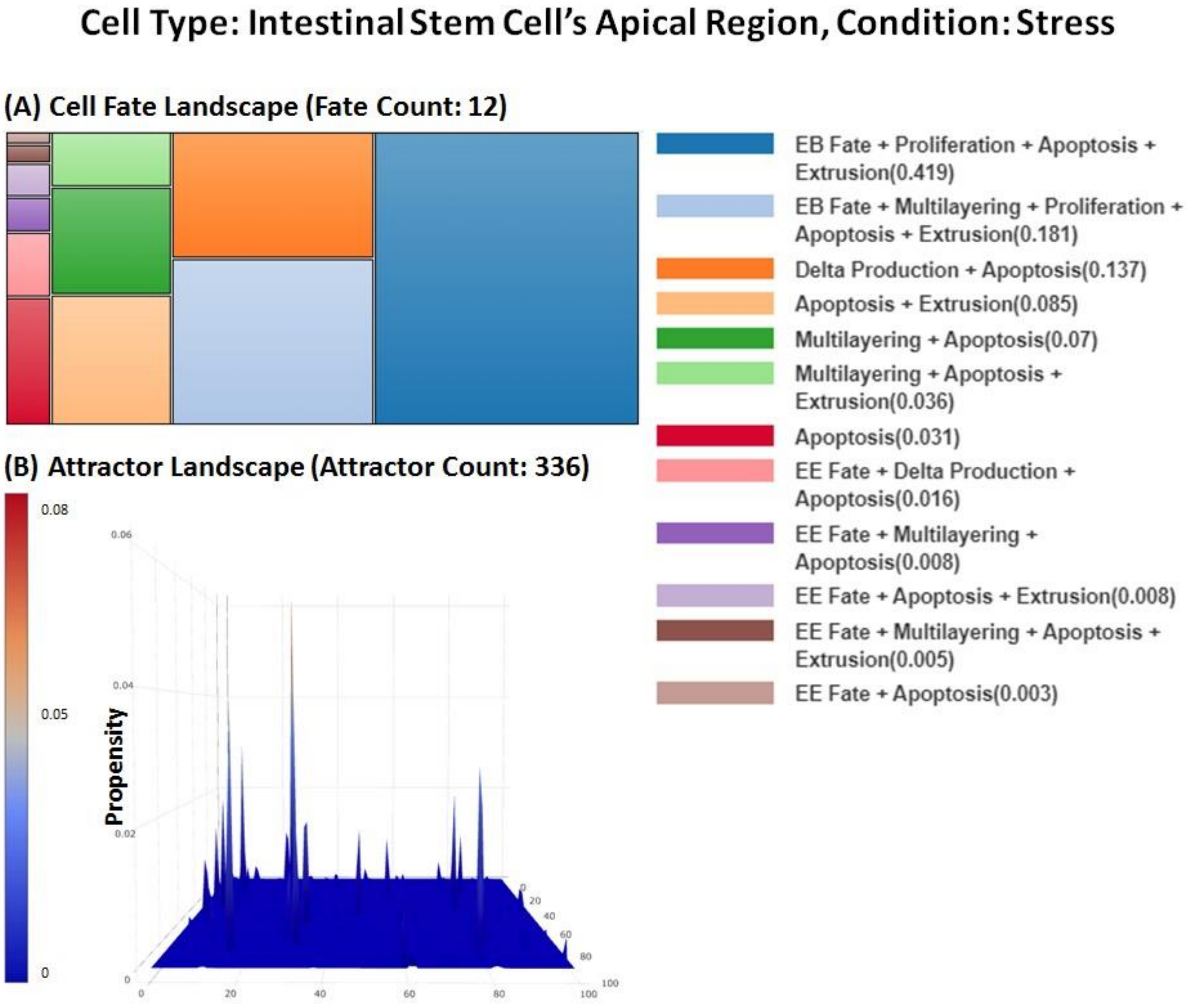

Supplementary Figure 9: Intestinal stem cell in apical region, cell fate outcomes in stress condition

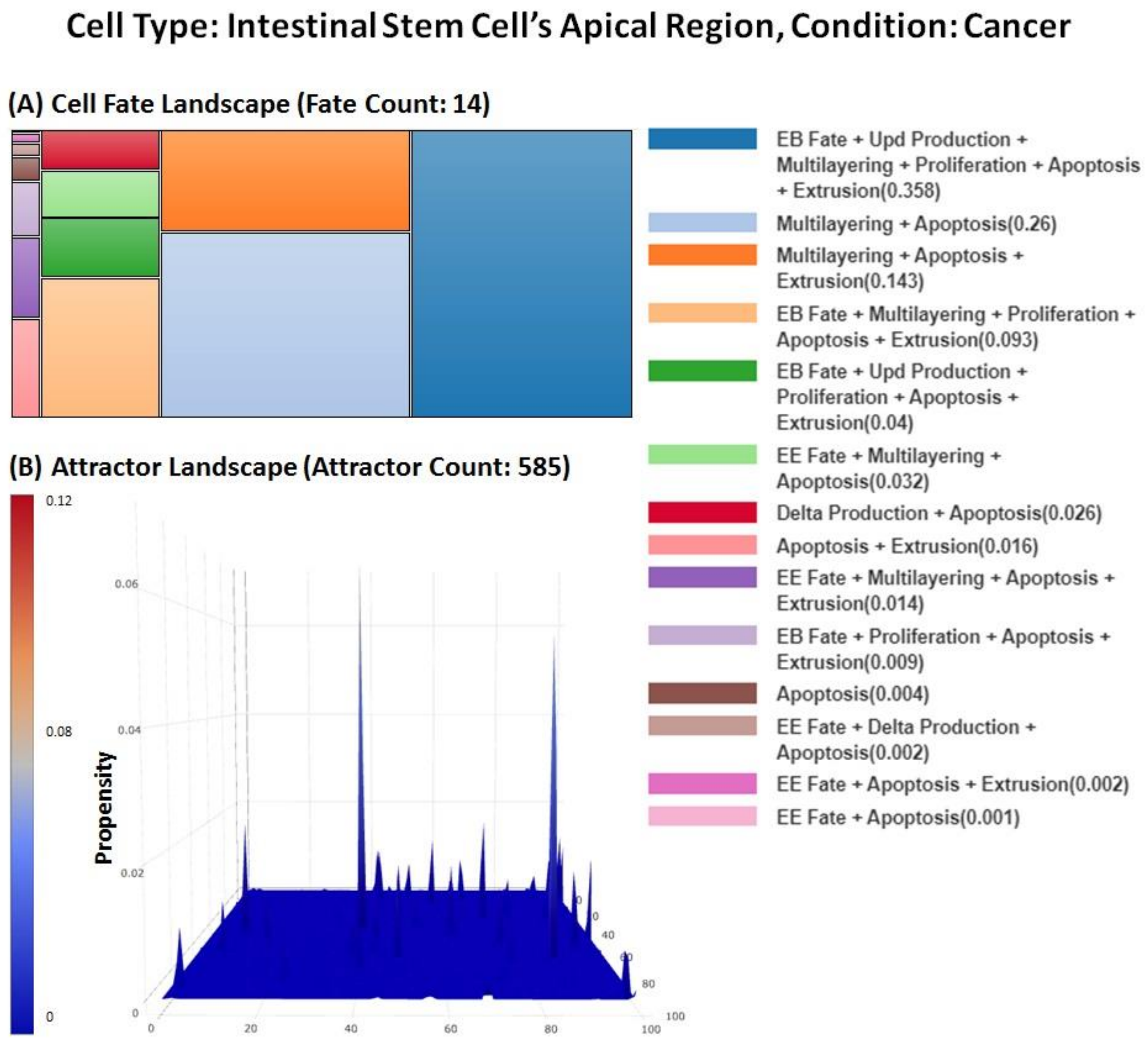

Supplementary Figure 10: Intestinal stem cell in apical region, cell fate outcomes in cancer condition

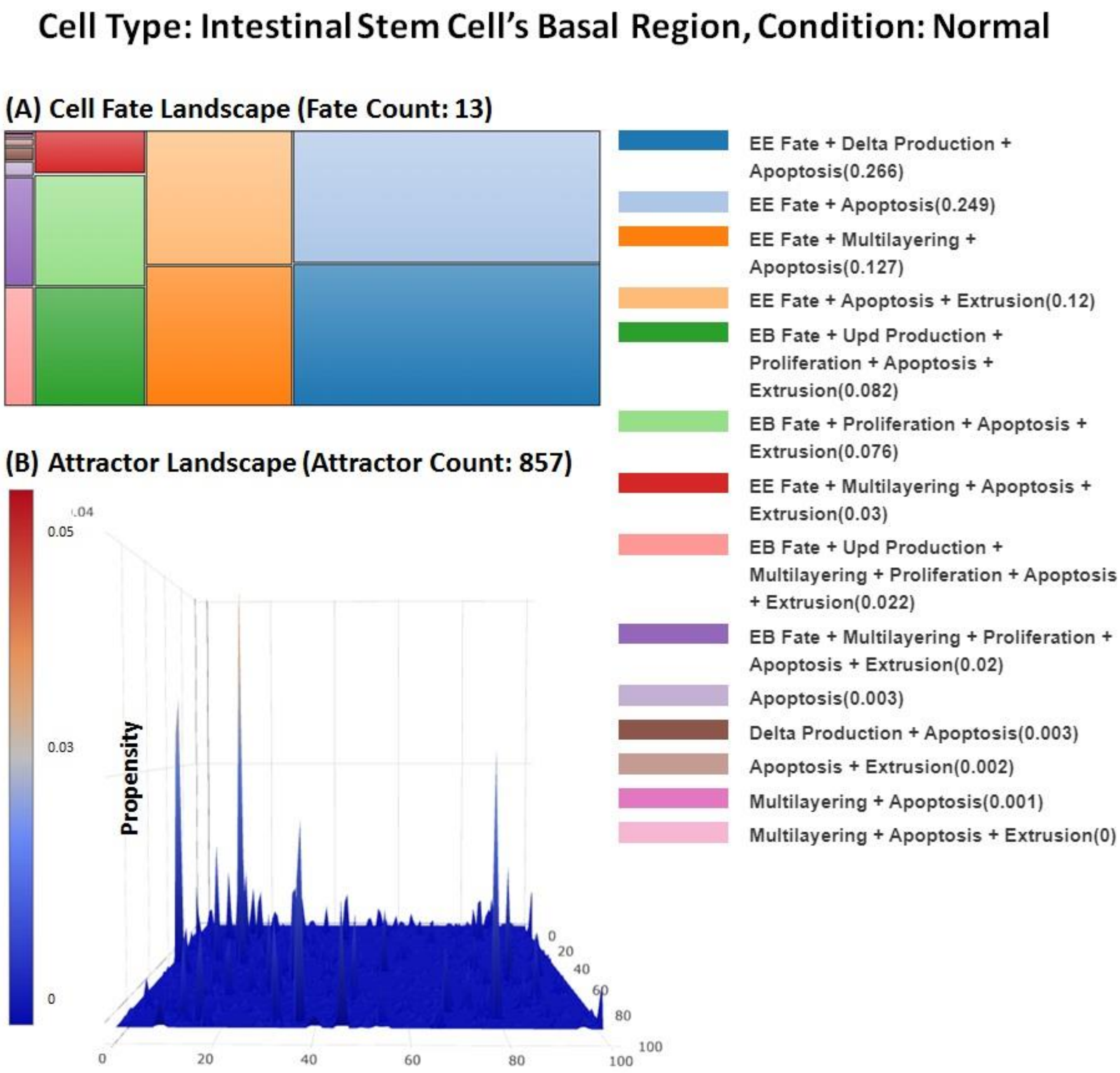

Supplementary Figure 11: Intestinal stem cell in basal region, cell fate outcomes in normal condition

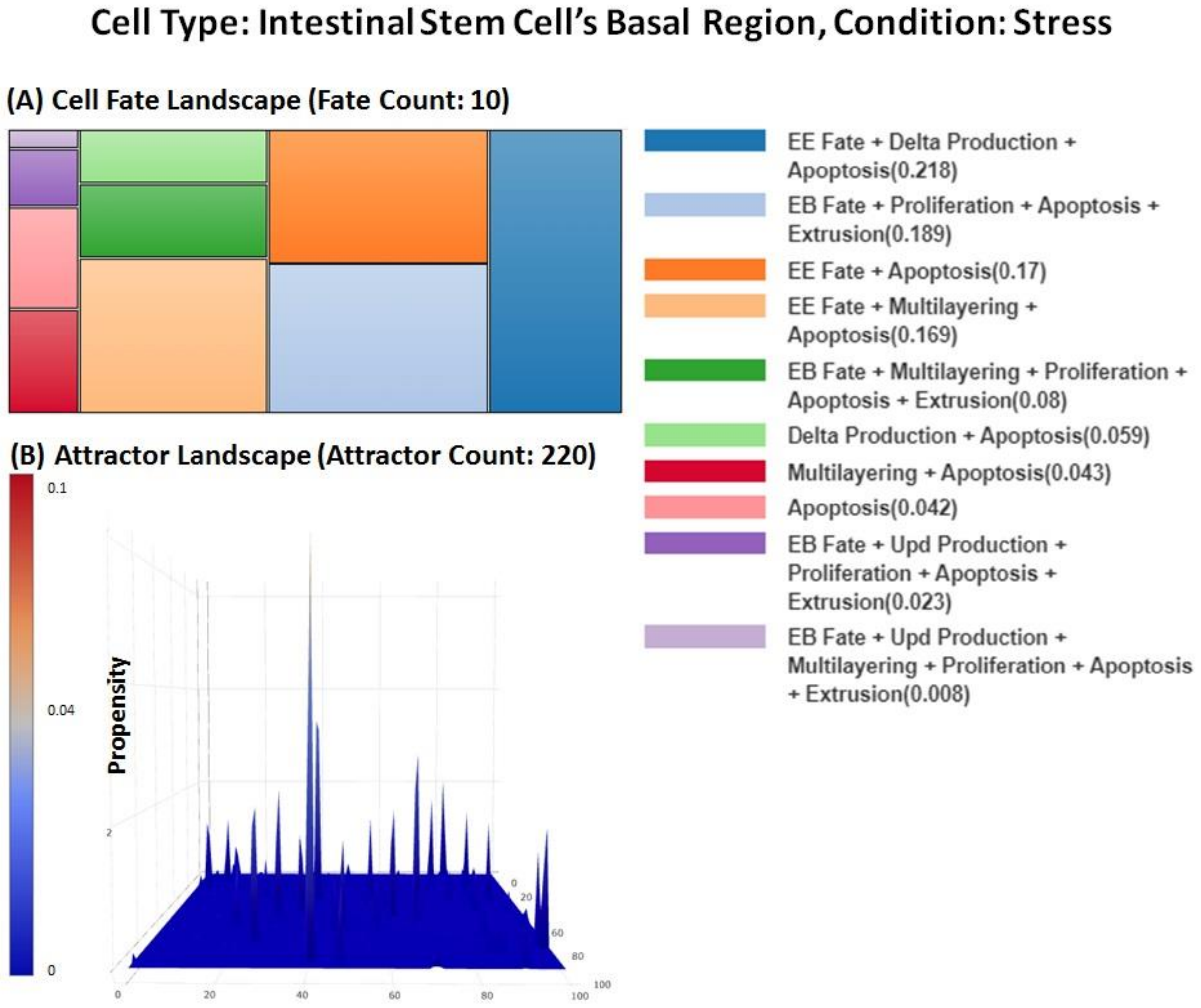

Supplementary Figure 12: Intestinal stem cell in basal region, cell fate outcomes in stress condition

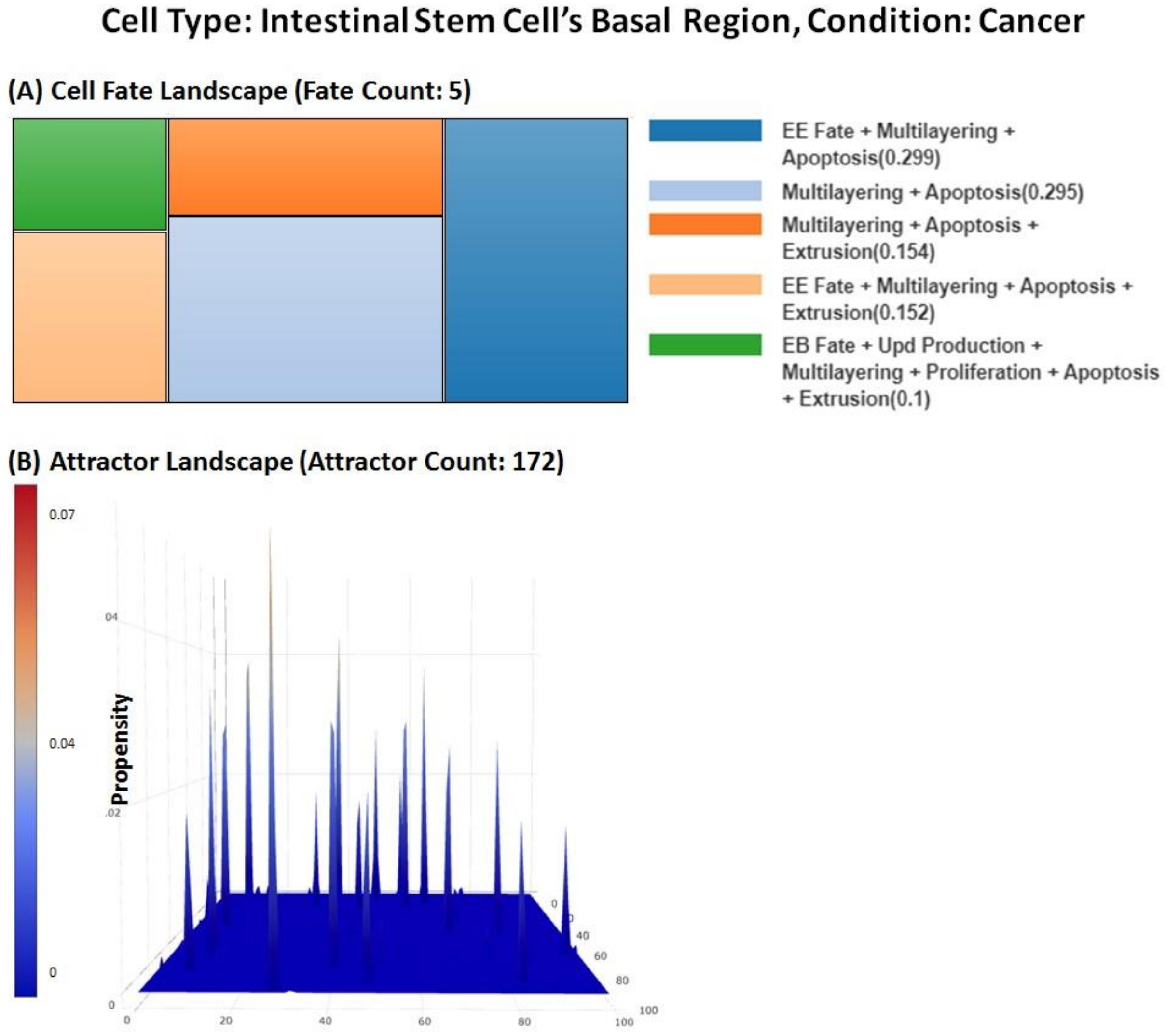

Supplementary Figure 13: Intestinal stem cell in basal region, cell fate outcomes in cancer condition

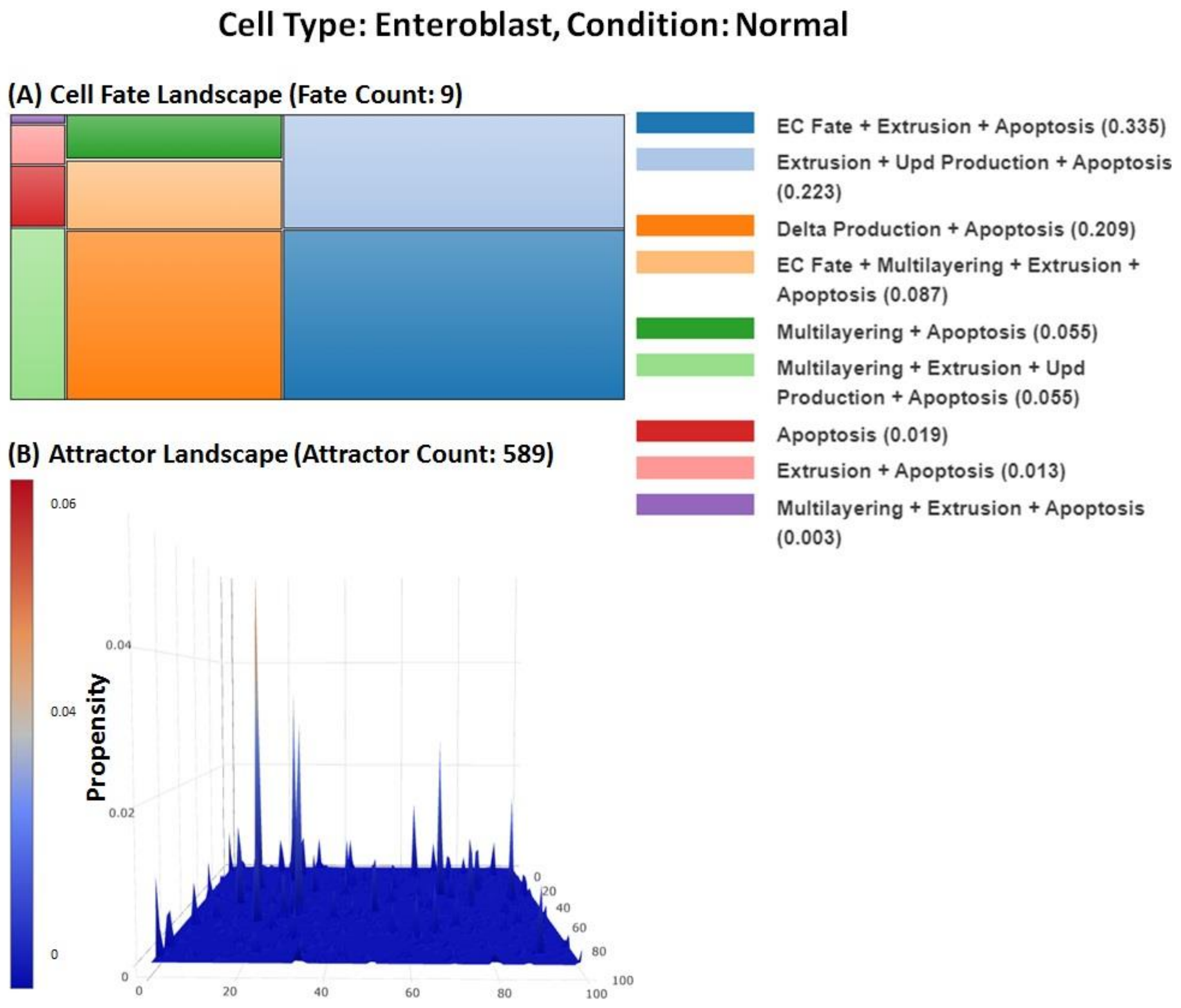

Supplementary Figure 14: Enteroblast, cell fate outcomes in normal condition

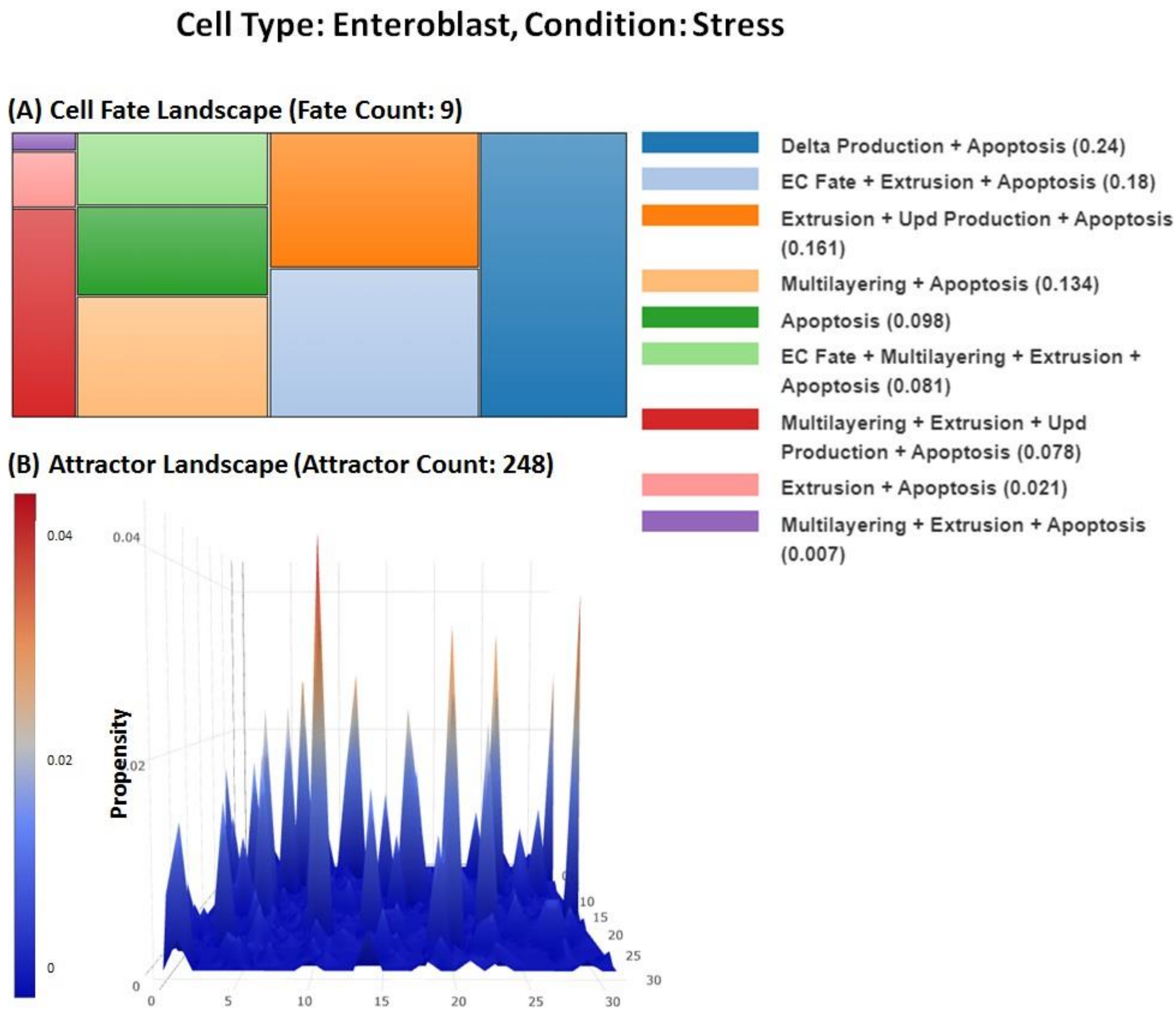

Supplementary Figure 15: Enteroblast, cell fate outcomes in stress condition

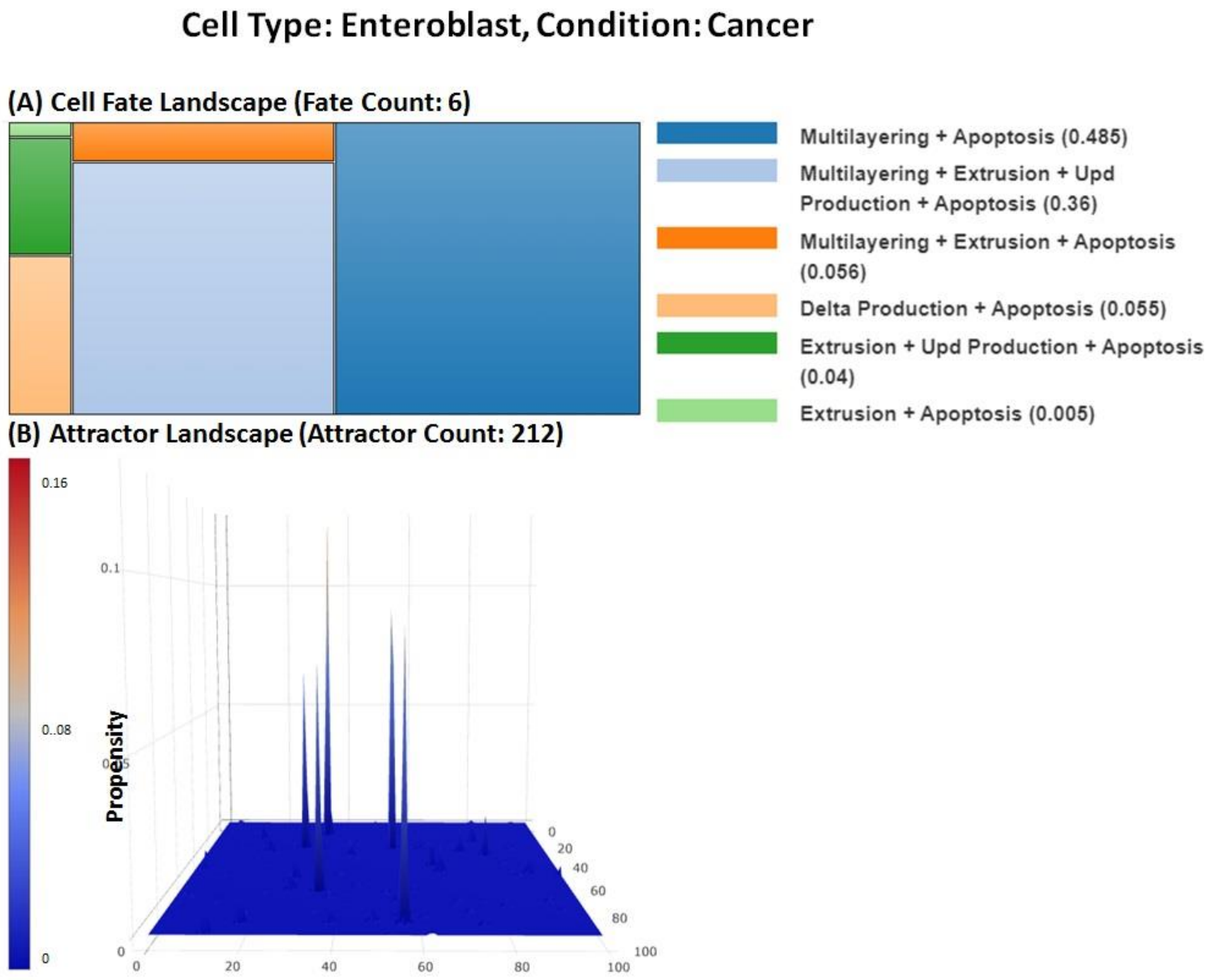

Supplementary Figure 16: Enteroblast, cell fate outcomes in cancer condition

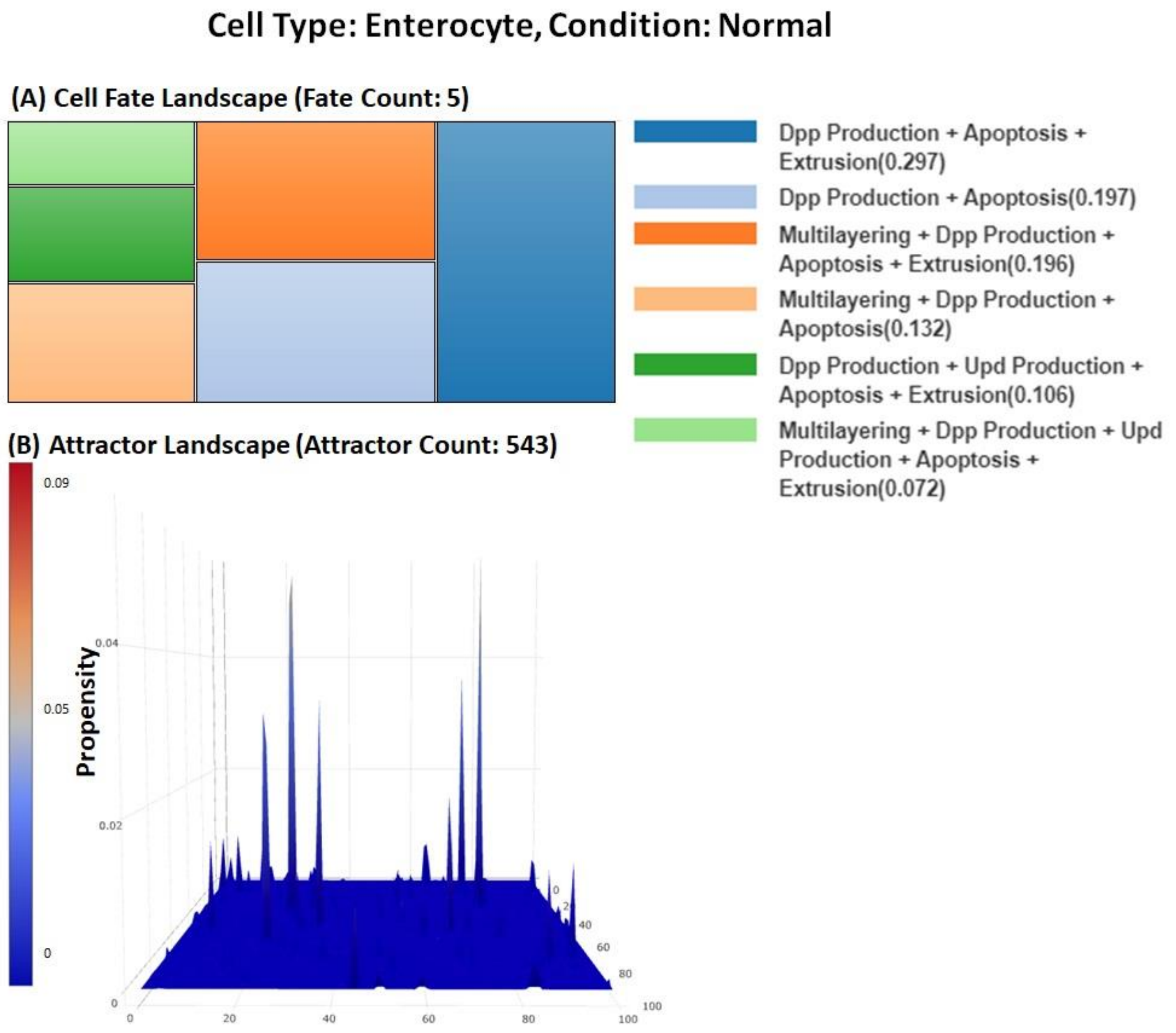

Supplementary Figure 17: Enterocyte, cell fate outcomes in normal condition

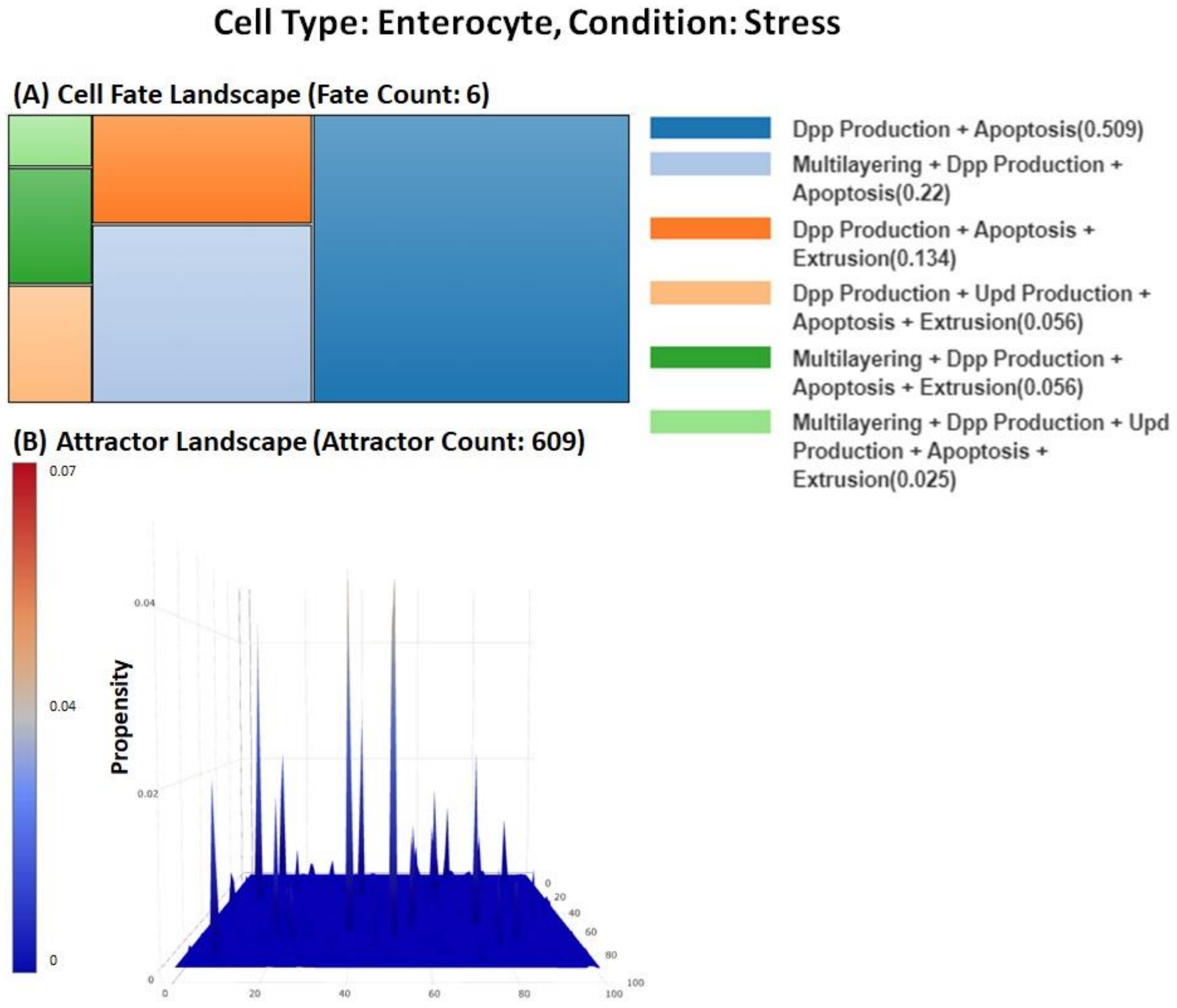

Supplementary Figure 18: Enterocyte, cell fate outcomes in stress condition

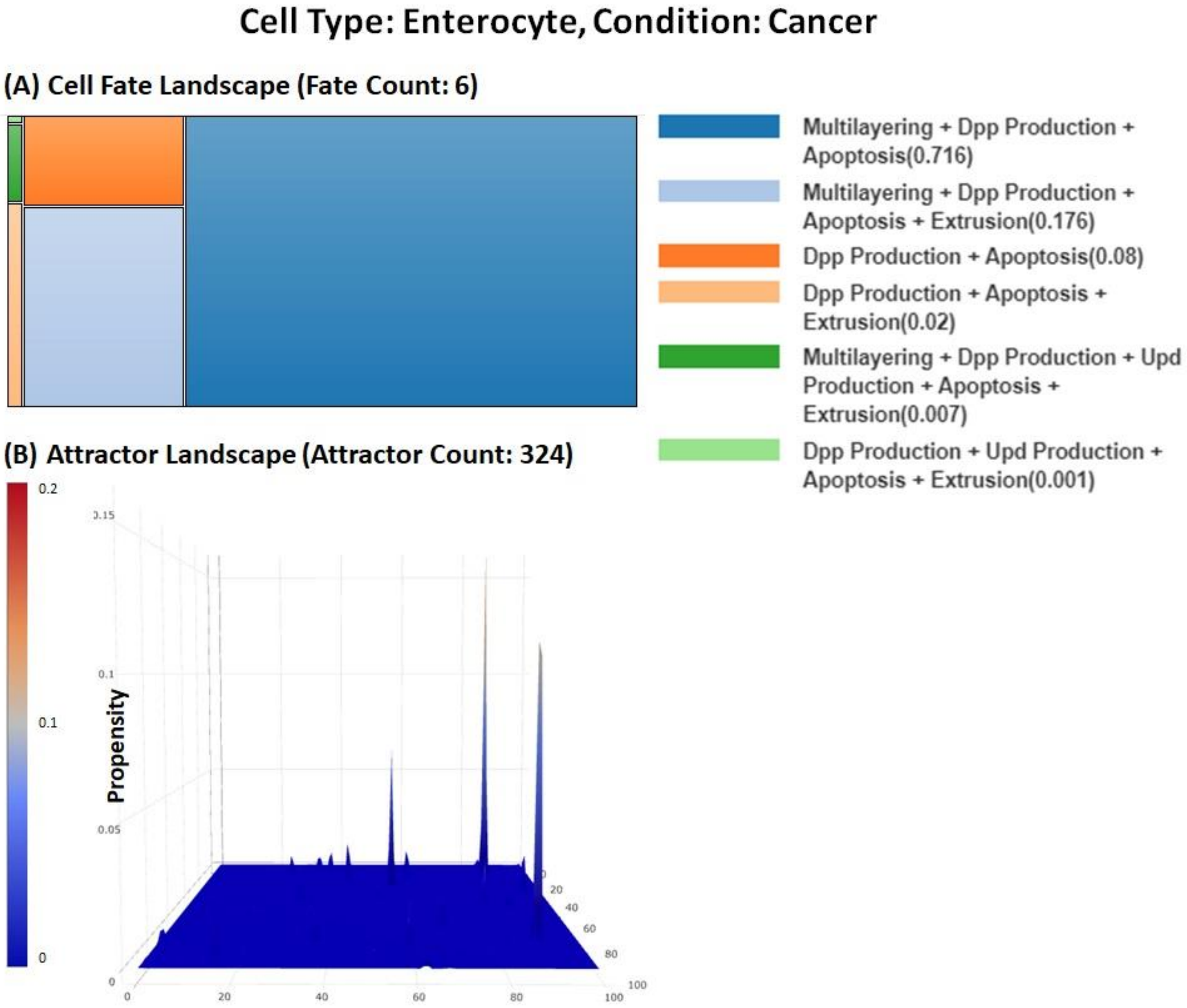

Supplementary Figure 19: Enterocyte, cell fate outcomes in cancer condition

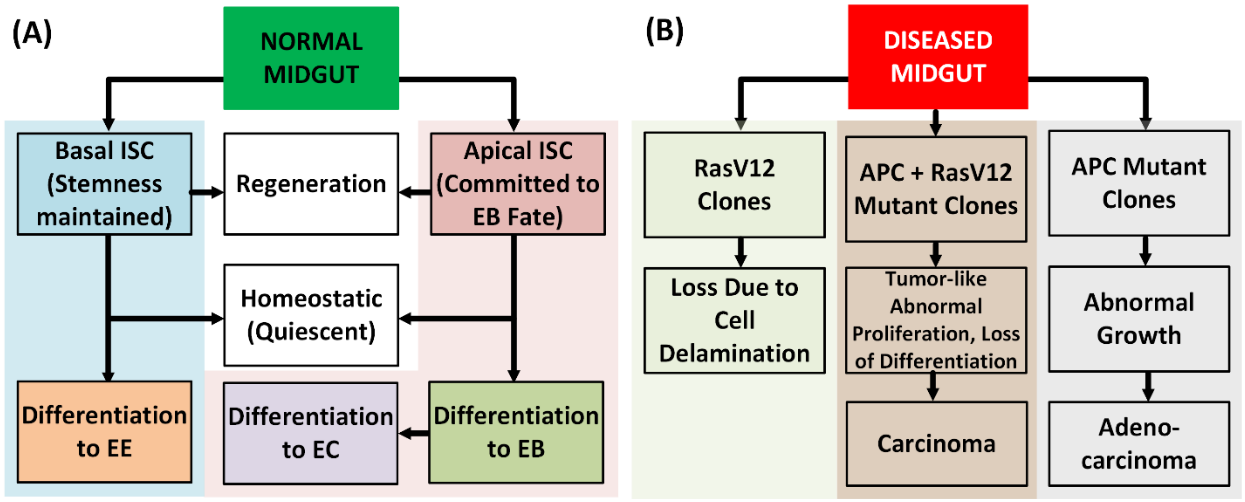

Supplementary Figure 20: Schematic of homeostasis, differentiation and tumorigenesis in normal and diseased midgut.

(A) In normal midgut, basal ISCs maintain stemness or differentiate into EE while apical ISCs get converted into EB. EBs can then differentiate into ECs under certain conditions; however, they mostly remain dormant in homeostatic conditions. **(B)** In diseased midgut, depending on the mutation type, the gut can either form adenocarcinoma or carcinoma. APC mutation can lead to development of an adenocarcinoma in the gut with a further Ras mutation can result in carcinoma.

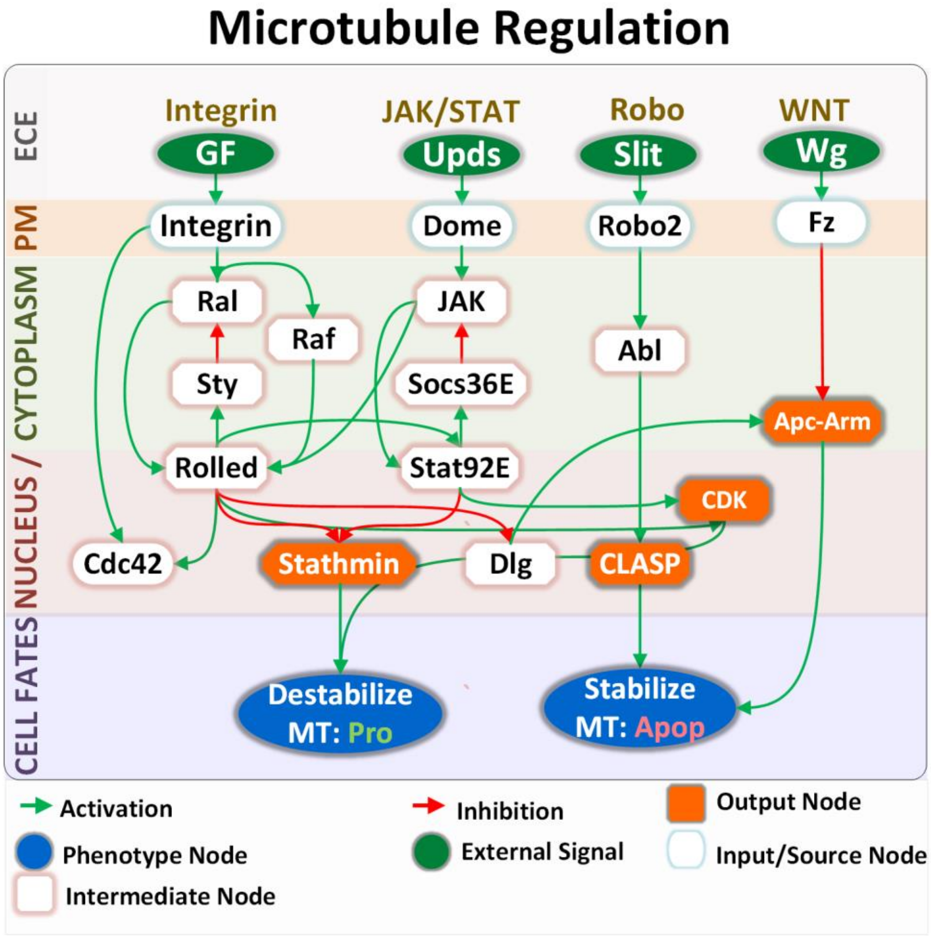

Supplementary Figure 21: Schematic representation of regulation in microtubule.
The inputs (green boxes) to these pathways are GF, Upds, Slit and Wg, respectively. Each input is mapped to the output through an intermediate layer of nodes. The outputs (orange boxes) include Stathmin, CLASP, CDK, and Apc-Arm. The output layer is used to program cell fates which includes Destabilize and Stabilize microtubule, corresponding to Proliferation and Apoptosis cell fates, respectively.

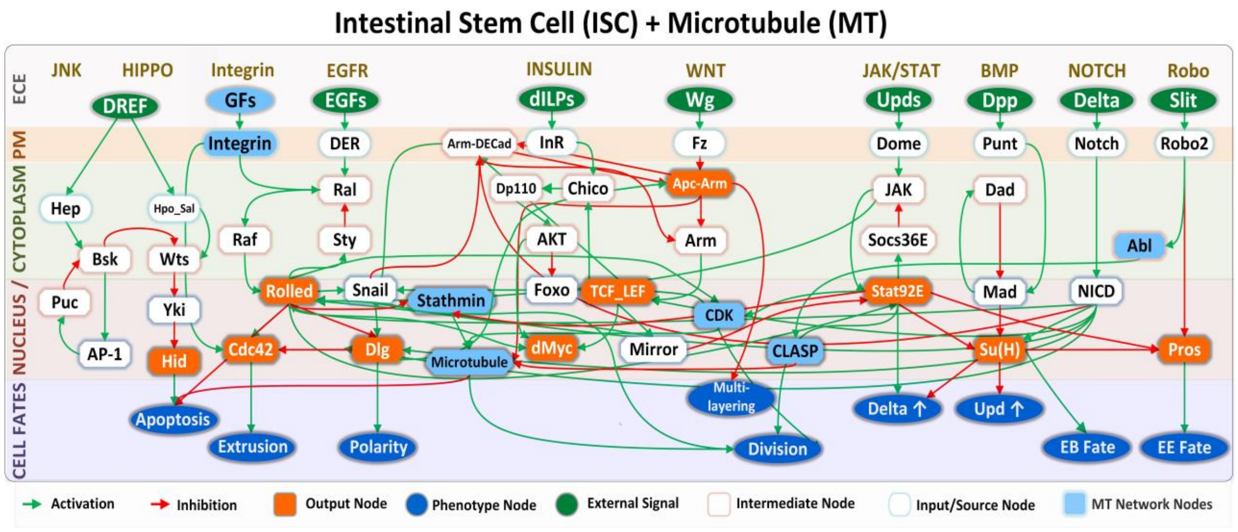

Supplementary Figure 22: Schematic representation of regulation in intestinal stem cells and microtubule network.

The inputs (green boxes) to these pathways are GF, EGFs, Wg, Upds, Dpp, Delta, DREF, dILPs, and Slit, respectively. Each input is mapped to the output through an intermediate layer of nodes. The outputs (orange boxes) include Rolled, Cdc42, Hid, Dlg, Apc-Arm, TCF-LEF, STAT92E, Su(H), Stathmin, CLASP, CDK, dMyc and Pros. The output layer is used to program cell fates which includes Extrusion, Apoptosis, Polarity, Division, Multilayering, Delta and Upd Production, EB Fate and EE Fates.

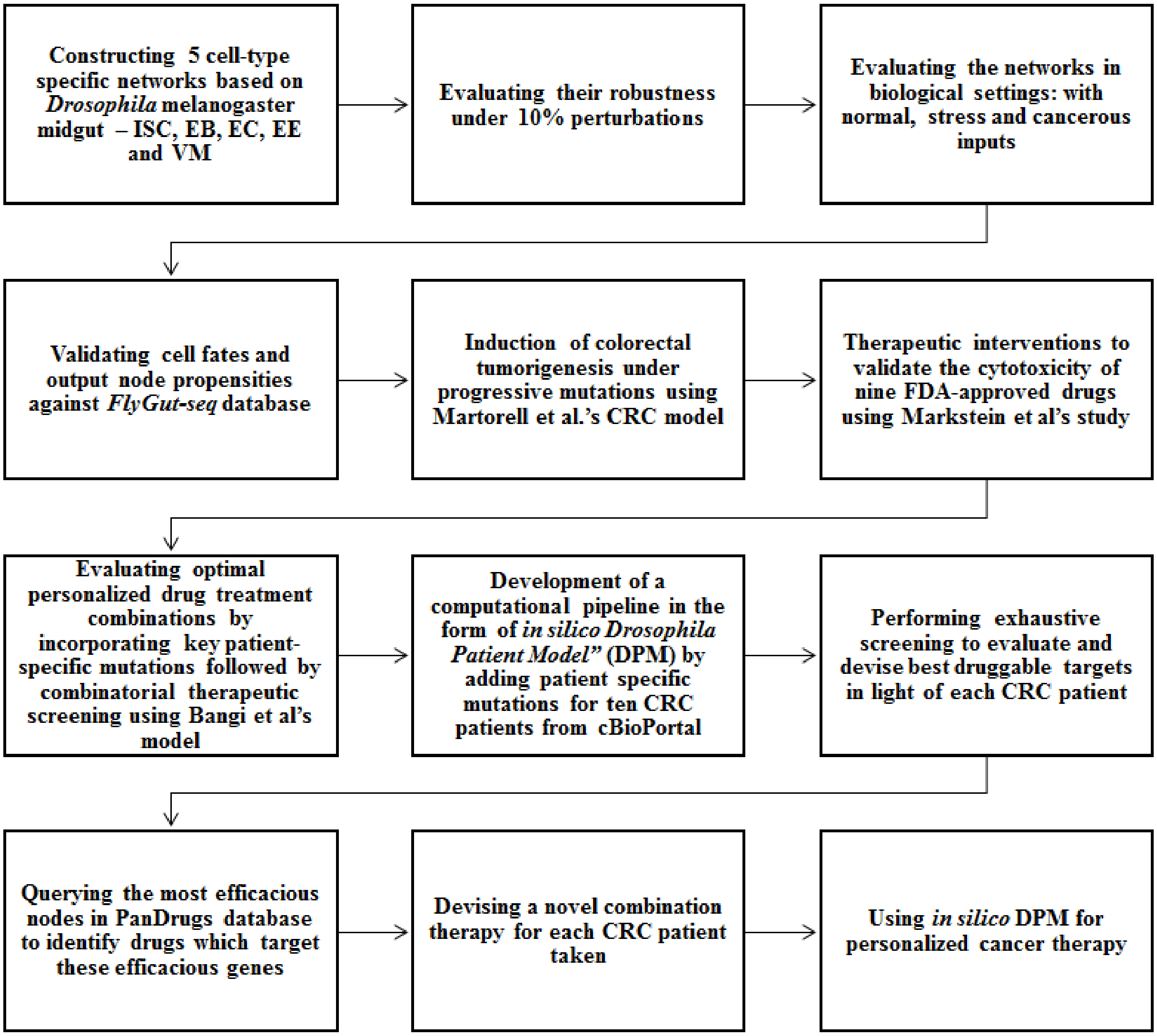

Supplementary Figure 23: The overall workflow of the study.

1.2. Supplementary Tables

Supplementary Table 1: Detailed node interaction rules and experimental evidences supporting different interactions and logical functions for Intestinal Stem Cells (ISC) model.

Intestinal Stem Cell						
**Source Nodes**	**Node Update Logic (TISON)**	**Target Nodes**	**Biological Names**	**Node Update Logic Equation (TISON)**	**Description**	**Reference**
Fz Arm-DECad	!ArmDECad && !Fz	ApcArm	Armadillo B-catenin	ApcArm = !ArmDECad && !Fz	Frizzled is receptor of WNT which activates wingless pathway by inhibting the formation of Apc-Arm. Thereby, Apc cannot degrade Arm. DE-Cad and Apc compete for binding with Arm (B-catenin) [7833051]	16443747, 7833051
Apc-Arm Arm-DECad	!ApcArm && !ArmDECad	Arm	Armadillo B-catenin	Arm = !ApcArm && !ArmDECad	DE-Cad and Apc compete for binding with Arm (B-catenin) [7833051]. Apc inhibits Arm [15169756]. DE-cad inhibit Arm [22174153]	15169756, 7833051, 22174153
Apc-Arm TCF_LEF Snail NICD	!Snail && (!ApcArm || !NICD) && TCFLEF	ArmDECad	Arm *Drosophila* E-Cadherin	ArmDECad = !Snail && (!ApcArm || !NICD) && TCFLEF	Snail which is a known repressor of DE-Cad [15983400]. Arm either binds to cadherin or Apc therefore they inhibit one another. Arm/TCF_LEF complex forms a transcription factor for expression of Arm-DECad (Wingless genes) [18617885, 22174153]. NICD increase so JNK increase [27574001] and JNK increase so its downstream Ecad decrease [25226030, 22174153]	17587826, 16918415, 16720643, 22174153, 15983400, 27574001, 25226030, 22174153

Rolled Dlg	Rolled || !Dlg	Cdc	Cell division control protein 42 homolog	Cdc = Rolled || !Dlg	PKC activates downstream Src which activates Rolled [19602257], therefore Rolled activates Cdc42, as Src activates Cdc42 [16449321]. Par6 is destabilized by Dlg, Par6 activates Cdc42, therefore, Dlg inhibits Cdc42 [24648766]. Rasv12 cells activation of Cdc42 [19287376]	16449321, 19602257, 24648766, 19287376
Mad	Mad	Dad	Daughters against dpp	Dad = Mad	Mad induces expression of Dad, a Dpp target gene.	10902180, 9335506
EGFs	EGFs	DER	Torpedo also known as EGFR receptor	DER = EGFs	Activated DER receptor activates Ral (fly hmolog of Ras) by phosphorylation. DER is a tyrosine kinase receptor and part of EGF receptor subfamily [1425358]	1425358
Rolled ArmDECad NICD	!Rolled || ArmDECad || NICD	Dlg	Discs Large	Dlg = !Rolled || ArmDECad || NICD	PKC inbitbits Dlg [19029932, 24648766], and PKC is activated by Ras[15037605], which activates Rolled[Kegg EGFR Signaling], therefore, rolled inhibits Dlg. Further, the loss of Dlg is activated by activated Ras expression [19029932]. NICD activates JNK [27574001] and JNK activates Dlg [16532034], so NICD activates Dlg. ArmDECad activates Dlg [19029932]	19029932, 24648766, 15037605, Kegg EGFR Signaling, 19029932, 27574001, 16532034, 19029932
Upds	Upds	Dome	Domeless	Dome = Upds	Dome is activated when its ligand Upd binds	24058767

Wg	Wg	Fz	Frizzled	Fz = Wg	Frizzled is receptor of WNT which activates wingless pathway	7833051
Rolled Yki	!Rolled || !Yki	Hid	Head involution defective Wrinkled	Hid = !Rolled || !Yki	Post-translational modifications of Hid prevent Hid-induced apoptosis [9814704]. For e.g. Rolled inhibts Hid by phosphorylating it [17183370]. Yorkie has anti-apoptotic function which inhibits apoptosis inhibiting gene hid [32485126] Yorkie inhibits bantam and bantem activates hid therefore, yorkie inhibits hid [31331981].	19554451, 17183370, 32485126, 9814704, 28102471, 31331981
Dome Socs36E	!SocsE && Dome	JAK	Hopscotch	JAK = !SocsE && Dome	When Upds bind to Dome, JAK get activated via phosphorylation. On the other hand, Socs36E inactivates JAK.	26807580, 19563763
Punt Dad	!Dad && Punt	Mad	Mothers against dpp	Mad = !Dad && Punt	Activated Punt phosphorylate Mad to activate it [24813173]. On the other hand, Dad antagonizes Mad activity [10902180]	24813173, 10902180, 9335506
TCF_LEF Rolled	TCFLEF || Rolled	Mir	Mirror	Mir = TCFLEF || Rolled	GRK activates EGFR which activates Rolled which inhibits CIC which then inhibts mirror, therefore, Rolled indirectly activates mirror[24127599, 27593379] also it is activated in EGFR signaling [10742112] . TCF activates Cyclin D1 [26721396], Cyclin D activates VEGF[16899588]. VEGF activates Rolled [20406854] which activates Mirror. Hence TCF_LEF activates Mirror.	24127599, 27593379, 10742112, 26721396, 16899588, Kegg VEGF Signaling, 20406854

Notch	Notch	NICD	N^{intra} Cleaved Intracellular Domain of Notch	NICD = Notch	Activated Notch undergoes cleavages to form NICD. Wnt inhibits Notch signaling [18806781].	17362357, 20816404, 18806781
Delta	Delta	Notch	Notch	Notch = Delta	Notch is receptor of Delta	7813766
Su(H) Stat92E Robo2	(!StatE || !SuH) && !Robo	Pros	Prospero	Pros = (!StatE || !SuH) && !Robo	Robo2 inhibits prospero activity [24931602]. Su(H) induces expression of E(spl) bHLH genes that produce bHLH transcriptional inhibitors that repress activity of achaete-scute factors [20147375]. Scute_Asense factors induce expression of Prospero [24931602]. Escargot represses Scute mediated transcription of Prospero [s41598-017-01138-z, 7600969]. However, Stat92E has been implicated in delayed repression of Escargot potentially at the level of transcription [25298397].	24931602, 7600969, 7958894, 24931602, s41598-017-01138-z, 20147375, 25298397
Dpp	Dpp	Punt	Punt	Punt = Dpp	Punt is a receptor of Dpp	21546910
DER Sty	DER && !Sty	Ral	Ras-related protein, inferred as Ras in the modal	Ral = DER && !Sty	Activated DER receptor activates Ral (fly hmolog of Ras) by phosphorylation. Sprouty inhibits the activity of Ras [24530508]	Kegg MAPK - Fly, 24530508
Slit	Slit	Robo	Robo2	Robo = Slit	Robo2 is a receptor of Slit	11239147

Raf JAK NICD	Raf ||JAK || NICD	Rolled	Erk MAP kinase	Rolled = Raf ||JAK || NICD	Ral (Ras-related protein in fly) activates Phl (Raf in humans) which goes on to activate Rolled by phosphorylating it [23484853]. Both JAK and NICD activate EGFR which activates Rolled. So Rolled gets activated by JAK [8793290, 28472194] and NICD as well [21622856].	Kegg MAPK - Fly, 8793290, 28472194, 21622856
TCF_LEF Rolled NICD	TCFLEF || Rolled || NICD	Snail	Snail	Snail = TCFLEF || Rolled || NICD	Rolled activates Snail [28415812]. TCF is needed for Snail activation [23029025]. NICD also activates Snail [29705809, 15197341]	28415812, 23029025, 29705809, 15197341
StatE	StatE	SocsE	Suppressor of cytokine signaling at 36E	SocsE = StatE	Stat92E induces expression of Jak/Stat repressor, Socs36E	19563763
JAK Mirror Rolled	JAK || !Mir || Rolled	StatE	Marelle D-STAT	StatE = JAK || !Mir || Rolled	JAK promotes activation and dimerization Stat92E. Mirror inhibits Stat92E [28045022]. Rolled (fly homolog of Erk) also activates Stat [27742579]	26807580, 19563763, 28045022, 18337767, 27742579
Rolled	Rolled	Sty	Sprouty	Sty = Rolled	Rolled activates Pointed which then induces expression of Sprouty	24530508
NICD Mad Stat92E	(!StatE || !Mad) && NICD	SuH	Suppressor of Hairless	SuH = (!StatE || !Mad) && NICD	NICD promotes activation of Su(H) mediated transcription of Notch genes by removing repression by Hairless [17362357]. Mad inhibits Su(H) activity [23861806, (28945500, inferred)]. Stat92E promotes expression of Hairless [26758761, 24077308]	28945500, 17362357, 20147375, 26758761, 24077308, 23861806

					which acts a repressor of Su(H) [20147375].	
Ral	Ral	Raf	Rapidly Accelerated Fibrosarcoma	Raf = Ral	Ras activates Raf [15035987]	15035987
Arm NICD	Arm || NICD	TCFLEF	T-cell factor/ lymphocyte enhancer factor-1/ pangolin	TCFLEF = Arm || NICD	Arm binds to and activates TCF/LEF transcription factors. NICD activates TCFLEF [28245235, 26592459]	Kegg WNT Signaling Fly, 26592459, 28245235
DREF	DREF	Hep	hemipterous	Hep = DREF	DREF activates Hep which in turn activates bsk	24752236
DREF	DREF	HpoSal	Salvador	HpoSal = DREF	DREF activates Hippo pathway such that it inhibits yki	25424907
Hep Puc	Hep ||!Puc	Bsk	basket	Bsk = Hep ||!Puc	Bsk is activates by Hep and inhibited by Puc	10.3934/genet.2014.1.20, 11402332
AP1	AP	Puc	puckered	Puc = AP	AP1 activates puckered	9472024, 11402332
Bsk	Bsk	Ap	Jun-related antigen	Ap = Bsk	Bsk compete to phosphorylate jun (ap1)	8946915, 11402332
Wts	!Wts	Yki	Yorkie	Yki = !Wts	Wts phosphorylates Yki and inhibits its transcriptional activity	17889654, 21808241
Ral Bsk HpoSal	!Bsk || HpoSal	Wts	warts	Wts = !Bsk || HpoSal	Hippo directly interacts with sav to activate wts [12941273, 21808241]. Wts is inhibited by Bsk [28174264]	12941273, 21808241, 28174264
DILPs	DILPs	InR	Insulin-like receptor	InR = DILPs	ILP1 activates InR1	22252538
InR	InR || Foxo	Chico	chico	Chico = InR || Foxo	Foxo activates Chico and InR activates Chico	30055320

Chico	Chico || Ral	Dp	Pi3K92E Dp110	Dp = Chico || Ral	Chico and Ral activates PI3K92E (Dp110) [30055320, 26119340]	30055320
Dp110	Dp	AKT	AKT1	AKT = Dp	PI3K (Dp110) activates its downstream target AKT [26508828]	26508828
AKT	!AKT	Foxo	forkhead box	Foxo = !AKT	Akt inhibits foxo through direct phosphorylation	21440577
AKT Rolled	AKT || Rolled || tcflef	dMyc	Myc	dMyc = AKT || Rolled || tcflef	AKT and TCFLEF promotes activation of myc [15241468, 31623618]. Rolled (erk) enables myc stabilization [22461507]	15241468, 22461507, 31623618

Supplementary Table 2: Detailed node interaction rules and experimental evidences supporting different interactions and logical functions for Enteroblast (EB) model.

Enteroblast Cell						
**Source Nodes**	**Node Update Logic (TISON)**	**Target Nodes**	**Biological Names**	**Node Update Logic Equation (TISON)**	**Description**	**Reference**
Fz ArmDECad	!ArmDECad && !fz	ApcArm	Armadillo B-catenin	ApcArm = !ArmDECad && !fz	Frizzled is receptor of WNT which activates wingless pathway by inhibting the formation of Apc-Arm. Thereby, Apc cannot degrade Arm. DE-Cad and Apc compete for binding with Arm (B-catenin)[7833051]	16443747, 7833051
Apc-Arm Arm-DECad	!ApcArm && !ArmDECad	Arm	Armadillo B-catenin	Arm = !ApcArm && !ArmDECad	DE-Cad and Apc compete for binding with Arm (B-catenin)[7833051]. Apc inhibits Arm [15169756]. DE-cad inhibit Arm [22174153]	15169756, 7833051, 22174153
Apc-Arm TCF_LEF Snail NICD	!Snail && (!ApcArm || !NICD) && TCFLEF	ArmDECad	Arm *Drosophila* E-Cadherin	ArmDECad = !Snail && (!ApcArm || !NICD) && TCFLEF	Snail which is a known repressor of DE-Cad [15983400]. Arm either binds to cadherin or Apc therefore they inhibit one another. Arm/TCF_LEF complex forms a transcription factor for expression of Arm-DECad (Wingless genes) [18617885, 22174153]. NICD increase so JNK increase [27574001] and JNK increase so its downstream Ecad decrease [25226030, 22174153]	17587826, 16918415, 16720643, 22174153, 15983400, 27574001, 25226030, 22174153
Rolled Dlg	Rolled || !Dlg	Cdc	Cell division control protein 42 homolog	Cdc = Rolled || !Dlg	PKC activates downstream Src which activates Rolled[19602257], therefore Rolled activates Cdc42, as Src activates Cdc42 [16449321]. Par6 is	16449321, 19602257, 24648766

					destabilized by Dlg, Par6 activates Cdc42, therefore, Dlg inhibits Cdc42 [24648766]	
Mad	Mad	Dad	Daughter against dpp	Dad = Mad	Mad induces expression of Dad, a Dpp target gene.	10902180, 9335506
EGFs	EGFs	DER	Torpedo	DER = EGFs	Activated DER receptor activates Ral (fly hmolog of Ras) by phosphorylation. DER is a tyrosine kinase receptor and part of EGF receptor subfamily [1425358]	1425358
Rolled ArmDECad NICD	!Rolled || ArmDECad || NICD	Dlg	Discs Large	Dlg = !Rolled || ArmDECad || NICD	PKC inbitbits Dlg [19029932, 24648766], and PKC is activated by Ras[15037605], which activates Rolled[Kegg EGFR Signaling], therefore, rolled inhibits Dlg. Further, the loss of Dlg is activated by activated Ras expression [19029932]. NICD activates JNK [27574001] and JNK activates Dlg [16532034], so NICD activates Dlg. ArmDECad activates Dlg [19029932]	19029932, 24648766, 15037605, Kegg EGFR Signaling, 19029932, 27574001, 16532034, 19029932
Upds	Upds	Dome	Domeless	Dome = Upds	Dome is activated when its ligand Upd binds	24058767
Wg	Wg	Fz	Frizzled	Fz = Wg	Frizzled is receptor of WNT which activates wingless pathway	7833051
Rolled Foxo NICD	!Rolled || !Yki	Hid	Head involution defective Wrinkled	Hid = !Rolled || !Yki	Post-translational modifications of Hid prevent Hid-induced apoptosis [9814704]. For e.g. Rolled inhibts Hid by phosphorylating it [17183370]. Yorkie has anti-apoptotic function which inhibits apoptosis inhibiting gene hid [32485126] Yorkie inhibits	19554451, 17183370, 32485126, 9814704, 28102471, 31331981

					bantam and bantem activates hid therefore, yorkie inhibits hid [31331981].	
Dome Socs36E	!SocsE && Dome	JAK	Hopscotch	JAK = !SocsE && Dome	When Upds bind to Dome, JAK get activated via phosphorylation. On the other hand, Socs36E inactivates JAK.	26807580, 19563763
Punt Dad	!Dad && Punt	Mad	Mothers against dpp	Mad = !Dad && Punt	Activated Punt phosphorylate Mad to activate it [24813173]. On the other hand, Dad antagonizes Mad activity [10902180]	24813173, 10902180, 9335506
TCF_LEF Rolled	TCFLEF || Rolled	Mirror	Mirror	Mirror = TCFLEF || Rolled	GRK activates EGFR which activates Rolled which inhibits CIC which inhibted mirror, therefore, Rolled activates mirror[24127599, 27593379 . TCF activates Cyclin D1 [26721396], Cyclin D activates VEGF[16899588]. VEGF activates Rolled which activates Mirror. Hence TCF_LEF activates Mirror.	24127599, 27593379, 26721396, 16899588, Kegg VEGF Signaling
Notch	Notch	NICD	N^{intra} Cleaved Intracellular Domain of Notch	NICD = Notch	Activated Notch undergoes cleavages to form NICD. Wnt inhibits Notch signaling [18806781].	17362357, 20816404, 18806781
Delta	Delta	Notch	Notch	Notch = Delta	Notch is receptor of Delta	7813766
Dpp	Dpp	Punt	Punt	Punt = Dpp	Punt is a receptor of Dpp	21546910
DER Sty	DER && !Sty	Ral	Ras-related protein	Ral = DER && !Sty	Activated DER receptor activates Ral by phosphorylation. Sprouty inhibits the activity of Ras [24530508]	Kegg MAPK - Fly, 24530508

Raf JAK NICD	Raf ||JAK || NICD	Rolled	Erk MAP kinase	Rolled = Raf ||JAK || NICD	Ral (Ras-related protein in fly) activates Phl (Raf in humans) which goes on to activate Rolled by phosphorylating it [23484853]. Both JAK and NICD activate EGFR which activates Rolled. So Rolled gets activated by JAK[8793290, 28472194] and NICD as well[21622856].	Kegg MAPK - Fly, 8793290, 28472194, 21622856
TCF_LEF Rolled NICD	TCFLEF || Rolled || NICD	Snail	Snail	Snail = TCFLEF || Rolled || NICD	Rolled activates Snail[28415812]. TCF is needed for Snail activation [23029025]. NICD also activates Snail [29705809, 15197341]	28415812, 23029025, 29705809, 15197341
Stat92E	StatE	SocsE	Suppressor of cytokine signaling at 36E	SocsE = StatE	Stat92E induces expression of Jak/Stat repressor, Socs36E	19563763
JAK Mirror Rolled	JAK || !Mir || Rolled	StatE	Marelle D-STAT	StatE = JAK || !Mir || Rolled	JAK promotes activation and dimerization Stat92E. Mirror inhibits Stat92E [28045022]. Rolled (fly homolog of Erk) also activates Stat [27742579]	26807580, 19563763, 28045022, 18337767, 27742579
Rolled	Rolled	Sty	Sprouty	Sty = Rolled	Rolled activates Pointed which then induces expression of Sprouty	24530508
NICD Mad Stat92E	!Mad && NICD	SuH	Suppressor of Hairless	SuH = !Mad && NICD	NICD promotes activation of Su(H) mediated transcription of Notch genes by removing repression by Hairless [17362357].Mad inhibits Su(H) activity [23861806, (28945500, inferred)]. Stat92E promotes expression of Hairless [26758761, 24077308] which acts a repressor of Su(H) [20147375].	28945500, 17362357, 20147375, 26758761, 24077308
Ral	Ral	Raf	Rapidly Accelerate	Raf = Ral	Ras activates Raf [15035987]	15035987

			d Fibrosarcoma			
Arm NICD	Arm || NICD	TCFLEF	T-cell factor/ lymphocyte enhancer factor-1	TCFLEF = Arm || NICD	Arm binds to and activates TCF/LEF transcription factors. NICD activates TCFLEF [28245235, 26592459]	Kegg WNT Signaling Fly, 26592459, 28245235
DREF	DREF	Hep	hemipterous	Hep = DREF	DREF activates Hep which in turn activates bsk	24752236
DREF	DREF	HpoSal	Salvador	HpoSal = DREF	DREF activates Hippo pathway such that it inhibits yki	25424907
Hep Puc	Hep ||!Puc	Bsk	basket	Bsk = Hep ||!Puc	Bsk is activates by Hep and inhibited by Puc	10.3934/genet.2014.1.20, 11402332
AP1	AP	Puc	puckered	Puc = AP	AP1 activates puckered	9472024, 11402332
Bsk	Bsk	Ap	Jun-related antigen	Ap = Bsk	Bsk compete to phosphorylate jun (ap1)	8946915, 11402332
Wts	!Wts	Yki	Yorkie	Yki = !Wts	Wts phosphorylates Yki and inhibits its transcriptional activity	17889654, 21808241
Ral Bsk HpoSal	!Bsk || HpoSal	Wts	warts	Wts = !Bsk || HpoSal	Hippo directly interacts with sav to activate wts [12941273, 21808241]. Wts is inhibited by Bsk [28174264]	12941273, 21808241, 28174264
DILPs	DILPs	InR	Insulin-like receptor	InR = DILPs	ILP1 activates InR1	22252538
InR	InR || Foxo	Chico	chico	Chico = InR || Foxo	Foxo activates Chico and InR activates Chico	30055320
Chico	Chico || Ral	Dp	Pi3K92E Dp110	Dp = Chico || Ral	Chico and Ral activates PI3K92E (Dp110) [30055320, 26119340]	30055320
Dp110	Dp	AKT	AKT1	AKT = Dp	PI3K (Dp110) activates its downstream target AKT [26508828]	26508828
AKT	!AKT	Foxo	forkhead box	Foxo = !AKT	Akt inhibits foxo through direct phosphorylation	21440577
AKT Rolled	AKT || Rolled || tcflef	dMyc	Myc	dMyc = AKT || Rolled || tcflef	AKT and TCFLEF promotes activation of myc [15241468, 31623618].	15241468, 22461507, 31623618

					Rolled (erk) enables myc stabilization [22461507]	

Supplementary Table 3: Detailed node interaction rules and experimental evidences supporting different interactions and logical functions for Enterocyte (EC) model.

Enterocyte Cell						
**Source Nodes**	**Node Update Logic (TISON)**	**Target Nodes**	**Biological Names**	**Node Update Logic Equation (TISON)**	**Description**	**Reference**
Fz Arm-DECad	!ArmDECad && !Fz	ApcArm	Armadillo B-catenin	ApcArm = !ArmDECad && !Fz	Frizzled is receptor of WNT which activates wingless pathway by inhibting the formation of Apc-Arm. Thereby, Apc cannot degrade Arm. DE-Cad and Apc compete for binding with Arm (B-catenin)[7833051]	16443747, 7833051
Apc-Arm Arm-DECad	!ArmDECad && !ApcArm	Arm	Armadillo B-catenin	Arm = !ArmDECad && !ApcArm	DE-Cad and Apc compete for binding with Arm (B-catenin)[7833051]. Apc inhibits Arm [15169756]. DE-cad inhibit Arm [22174153]	15169756, 7833051, 22174153
Apc-Arm TCF_LEF Snail NICD	!Snail && (!ApcArm || !NICD) && TCFLEF	ArmDECad	Arm *Drosophila* E-Cadherin	ArmDECad = !Snail && (!ApcArm || !NICD) && TCFLEF	Snail which is a known repressor of DE-Cad [15983400]. Arm either binds to cadherin or Apc therefore they inhibit one another. Arm/TCF_LEF complex forms a transcription factor for expression of Arm-DECad (Wingless genes) [18617885, 22174153]. NICD increase so JNK increase [27574001] and JNK increase so its downstream Ecad decrease [25226030, 22174153]	17587826, 16918415, 16720643, 22174153, 15983400, 27574001, 25226030, 22174153
Rolled Dlg	Rolled || !Dlg	Cdc	Cell division control protein 42 homolog	Cdc = Rolled || !Dlg	PKC activates downstream Src which activates Rolled[19602257], therefore Rolled activates Cdc42, as Src activates Cdc42 [16449321]. Par6	16449321, 19602257, 24648766, 19287376

					is destabilized by Dlg, Par6 activates Cdc42, therefore, Dlg inhibits Cdc42 [24648766]. Rasv12 cells activation of Cdc42[19287376]	
Mad	Mad	Dad	Daughter against dpp	Dad = Mad	Mad induces expression of Dad, a Dpp target gene.	10902180, 9335506
EGFs	EGFs	DER	Torpedo	DER = EGFs	Activated DER receptor activates Ral (fly hmolog of Ras) by phosphorylation. DER is a tyrosine kinase receptor and part of EGF receptor subfamily [1425358]	1425358
Rolled ArmDE Cad NICD	!Rolled || ArmDECad || NICD	Dlg	Discs Large	Dlg = !Rolled || ArmDECad || NICD	PKC inbitbits Dlg [19029932, 24648766], and PKC is activated by Ras [15037605], which activates Rolled [Kegg EGFR Signaling], therefore, rolled inhibits Dlg. Further, the loss of Dlg is activated by activated Ras expression [19029932]. NICD activates JNK [27574001] and JNK activates Dlg [16532034], so NICD activates Dlg. ArmDECad activates Dlg [19029932]	19029932, 24648766, 15037605, Kegg EGFR Signaling, 19029932, 27574001, 16532034, 19029932
Wg	Wg	Fz	Frizzled	Fz = Wg	Frizzled is receptor of WNT which activates wingless pathway	7833051
Rolled Foxo NICD	!Rolled || !Yki	Hid	Head involution defective Wrinkled	Hid = !Rolled || !Yki	Post-translational modifications of Hid prevent Hid-induced apoptosis [9814704]. For e.g. Rolled inhibts Hid by phosphorylating it [17183370]. Yorkie has anti-apoptotic function which inhibits apoptosis inhibiting gene hid [32485126] Yorkie inhibits bantam and	19554451, 17183370, 32485126, 9814704, 28102471, 31331981

					bantem activates hid therefore, yorkie inhibits hid [31331981].	
Punt Dad	!Dad && Punt	Mad	Mothers against dpp	Mad = !Dad && Punt	Activated Punt phosphorylate Mad to activate it [24813173]. On the other hand, Dad antagonizes Mad activity [10902180]	24813173, 10902180, 9335506
Notch	Notch	NICD	N^{intra} Cleaved Intracellular Domain of Notch	NICD = Notch	Activated Notch undergoes cleavages to form NICD. Wnt inhibits Notch signaling [18806781].	17362357, 20816404, 18806781
Delta	Delta	Notch	Notch	Notch = Delta	Notch is receptor of Delta	7813766
Dpp	Dpp	Punt	Punt	Punt = Dpp	Punt is a receptor of Dpp	21546910
DER Sty	DER && !Sty	Ral	Ras-related protein	Ral = DER && !Sty	Activated DER receptor activates Ral by phosphorylation. Sprouty inhibits the activity of Ras [24530508]	Kegg MAPK - Fly, 24530508
Raf NICD	Raf || NICD	Rolled	Erk MAP kinase	Rolled = Raf || NICD	Ral (Ras-related protein in fly) activates Phl (Raf in humans) which goes on to activate Rolled by phosphorylating it [23484853]. Both JAK and NICD activate EGFR which activates Rolled. So Rolled (fly homolog of Erk) gets activated by NICD as well [21622856].	Kegg MAPK - Fly, 21622856
TCF_LEF Rolled NICD	TCFLEF || Rolled || NICD	Snail	Snail	Snail = TCFLEF || Rolled || NICD	Rolled activates Snail [28415812]. TCF is needed for Snail activation [23029025]. NICD also activates Snail [29705809, 15197341]	28415812, 23029025, 29705809, 15197341
Rolled	Rolled	Sty	Sprouty	Sty = Rolled	Rolled activates Pointed which then induces expression of Sprouty	24530508

NICD Mad Stat92E	NICD && !Mad	SuH	Suppressor of Hairless	SuH = NICD && !Mad	NICD promotes activation of Su(H) mediated transcription of Notch genes by removing repression by Hairless [17362357]. Mad inhibits Su(H) activity [23861806, (28945500, inferred)]. Stat92E promotes expression of Hairless [26758761, 24077308] which acts a repressor of Su(H) [20147375].	28945500, 17362357, 20147375, 26758761, 24077308
Ral	Ral	Raf	Rapidly Accelerated Fibrosarcoma	Raf = Ral	Ras activates Raf [15035987]	15035987
Arm NICD	Arm || NICD	TCFLEF	T-cell factor/ lymphocyte enhancer factor-1	TCFLEF = Arm || NICD	Arm binds to and activates TCF/LEF transcription factors. NICD activates TCFLEF [28245235, 26592459]	Kegg WNT Signaling Fly, 26592459, 28245235
DREF	DREF	Hep	hemipterous	Hep = DREF	DREF activates Hep which in turn activates bsk	24752236
DREF	DREF	HpoSal	Salvador	HpoSal = DREF	DREF activates Hippo pathway such that it inhibits yki	25424907
Hep Puc	Hep ||!Puc	Bsk	basket	Bsk = Hep ||!Puc	Bsk is activates by Hep and inhibited by Puc	10.3934/genet.2014.1.20, 11402332
AP1	AP	Puc	puckered	Puc = AP	AP1 activates puckered	9472024, 11402332
Bsk	Bsk	Ap	Jun-related antigen	Ap = Bsk	Bsk compete to phosphorylate jun (ap1)	8946915, 11402332
Wts	!Wts	Yki	Yorkie	Yki = !Wts	Wts phosphorylates Yki and inhibits its transcriptional activity	17889654, 21808241
Ral Bsk HpoSal	!Bsk || HpoSal	Wts	warts	Wts = !Bsk || HpoSal	Hippo directly interacts with sav to activate wts [12941273, 21808241]. Wts is inhibited by Bsk [28174264]	12941273, 21808241, 28174264

DILPs	DILPs	InR	Insulin-like receptor	InR = DILPs	ILP1 activates InR1	22252538
InR	InR || Foxo	Chico	chico	Chico = InR || Foxo	Foxo activates Chico and InR activates Chico	30055320
Chico	Chico || Ral	Dp	Pi3K92E Dp110	Dp = Chico || Ral	Chico and Ral activates PI3K92E (Dp110) [30055320, 26119340]	30055320
Dp110	Dp	AKT	AKT1	AKT = Dp	PI3K (Dp110) activates its downstream target AKT [26508828]	26508828
AKT	!AKT	Foxo	forkhead box	Foxo = !AKT	Akt inhibits foxo through direct phosphorylation	21440577
AKT Rolled	AKT || Rolled || tcflef	dMyc	Myc	dMyc = AKT || Rolled || tcflef	AKT and TCFLEF promotes activation of myc [15241468, 31623618]. Rolled (erk) enables myc stabilization [22461507]	15241468, 22461507, 31623618

Supplementary Table 4: Detailed node interaction rules and experimental evidences supporting different interactions and logical functions for Enteroendocrine (EE) model.

Enteroendocrine Cell						
**Source Nodes**	**Node Update Logic (TISON)**	**Target Nodes**	**Biological Names**	**Node Update Logic Equation (TISON)**	**Description**	**Reference**
Fz Arm-DECad	!ArmDECad && !Fz	ApcArm	Armadillo B-catenin	ApcArm = !ArmDECad && !Fz	Frizzled is receptor of WNT which activates wingless pathway by inhibting the formation of Apc-Arm. Thereby, Apc cannot degrade Arm. DE-Cad and Apc compete for binding with Arm (B-catenin)[7833051]	16443747, 7833051
Apc-Arm Arm-DECad	!ArmDECad && !ApcArm	Arm	Armadillo B-catenin	Arm = !ArmDECad && !ApcArm	DE-Cad and Apc compete for binding with Arm (B-catenin)[7833051]. Apc inhibits Arm [15169756]. DE-cad inhibit Arm [22174153]	15169756, 7833051, 22174153
Apc-Arm TCF_LEF Snail NICD	!Snail && (!ApcArm || !NICD) && TCFLEF	ArmDECad	Arm *Drosophila* E-Cadherin	ArmDECad = !Snail && (!ApcArm || !NICD) && TCFLEF	Snail which is a known repressor of DE-Cad [15983400]. Arm either binds to cadherin or Apc therefore they inhibit one another. Arm/TCF_LEF complex forms a transcription factor for expression of Arm-DECad (Wingless genes) [18617885, 22174153]. NICD increase so JNK increase [27574001] and JNK increase so its downstream Ecad decrease [25226030, 22174153]	17587826, 16918415, 16720643, 22174153, 15983400, 27574001, 25226030, 22174153

Rolled Dlg	Rolled || !Dlg	Cdc	Cell division control protein 42 homolog	Cdc = Rolled || !Dlg	PKC activates downstream Src which activates Rolled[19602257], therefore Rolled activates Cdc42, as Src activates Cdc42 [16449321]. Par6 is destabilized by Dlg, Par6 activates Cdc42, therefore, Dlg inhibits Cdc42 [24648766]. Rasv12 cells activation of Cdc42 [19287376]	16449321, 19602257, 24648766, 19287376
Mad	Mad	Dad	Daughter against dpp	Dad = Mad	Mad induces expression of Dad, a Dpp target gene.	10902180, 9335506
EGFs	EGFs	DER	Torpedo	DER = EGFs	Activated DER receptor activates Ral (fly hmolog of Ras) by phosphorylation. DER is a tyrosine kinase receptor and part of EGF receptor subfamily [1425358]	1425358
Rolled ArmDECad NICD	!Rolled || ArmDECad || NICD	Dlg	Discs Large	Dlg = !Rolled || ArmDECad || NICD	PKC inbitbits Dlg [19029932, 24648766], and PKC is activated by Ras[15037605], which activates Rolled[Kegg EGFR Signaling], therefore, rolled inhibits Dlg. Further, the loss of Dlg is activated by activated Ras expression [19029932]. NICD activates JNK [27574001] and JNK activates Dlg [16532034], so NICD activates Dlg. ArmDECad activates Dlg [19029932]	19029932, 24648766, 15037605, Kegg EGFR Signaling, 19029932, 27574001, 16532034, 19029932
Wg	Wg	Fz	Frizzled	Fz = Wg	Frizzled is receptor of WNT which activates wingless pathway	7833051

Rolled Foxo NICD	!Rolled || !Yki	Hid	Head involution defective Wrinkled	Hid = !Rolled || !Yki	Post-translational modifications of Hid prevent Hid-induced apoptosis [9814704]. For e.g. Rolled inhibts Hid by phosphorylating it [17183370]. Yorkie has anti-apoptotic function which inhibits apoptosis inhibiting gene hid [32485126] Yorkie inhibits bantam and bantem activates hid therefore, yorkie inhibits hid [31331981].	19554451, 17183370, 32485126, 9814704, 28102471, 31331981
Punt Dad	!Dad && Punt	Mad	Mothers against dpp	Mad = !Dad && Punt	Activated Punt phosphorylate Mad to activate it [24813173]. On the other hand, Dad antagonizes Mad activity [10902180]	24813173, 10902180, 9335506
Notch	Notch	NICD	N^{intra} Cleaved Intracellula r Domain of Notch	NICD = Notch	Activated Notch undergoes cleavages to form NICD. Wnt inhibits Notch signaling [18806781].	17362357, 20816404, 18806781
Delta	Delta	Notch	Notch	Notch = Delta	Notch is receptor of Delta	7813766
Dpp	Dpp	Punt	Punt	Punt = Dpp	Punt is a receptor of Dpp	21546910
DER Sty	DER && !Sty	Ral	Ras-related protein	Ral = DER && !Sty	Activated DER receptor activates Ral by phosphorylation. Sprouty inhibits the activity of Ras [24530508]	Kegg MAPK - Fly, 24530508
Raf NICD	Raf || NICD	Rolled	Erk MAP kinase	Rolled = Raf || NICD	Ral (Ras-related protein in fly) activates Phl (Raf in humans) which goes on to activate Rolled by phosphorylating it [23484853]. Both JAK and NICD activate EGFR which activates Rolled. So Rolled gets activated by NICD as well[21622856].	Kegg MAPK - Fly, 21622856

TCF_LEF Rolled NICD	TCFLEF || Rolled || NICD	Snail	Snail	Snail = TCFLEF || Rolled || NICD	Rolled activates Snail[28415812]. TCF is needed for Snail activation [23029025]. NICD also activates Snail [29705809, 15197341]	28415812, 23029025, 29705809, 15197341
Rolled	Rolled	Sty	Sprouty	Sty = Rolled	Rolled activates Pointed which then induces expression of Sprouty	24530508
NICD Mad Stat92E	NICD && !Mad	SuH	Suppressor of Hairless	SuH = NICD && !Mad	NICD promotes activation of Su(H) mediated transcription of Notch genes by removing repression by Hairless [17362357]. Mad inhibits Su(H) activity [23861806, (28945500, inferred)].Stat92E promotes expression of Hairless [26758761, 24077308] which acts a repressor of Su(H) [20147375].	28945500, 17362357, 20147375, 26758761, 24077308
Ral	Ral	Raf	Rapidly Accelerated Fibrosarcoma	Raf = Ral	Ras activates Raf [15035987]	15035987
Arm NICD	Arm || NICD	TCFLEF	T-cell factor/ lymphocyte enhancer factor-1	TCFLEF = Arm || NICD	Arm binds to and activates TCF/LEF transcription factors. NICD activates TCFLEF [28245235, 26592459]	Kegg WNT Signaling Fly, 26592459, 28245235
DREF	DREF	Hep	hemipterous	Hep = DREF	DREF activates Hep which in turn activates bsk	24752236
DREF	DREF	HpoSal	Salvador	HpoSal = DREF	DREF activates Hippo pathway such that it inhibits yki	25424907
Hep Puc	Hep ||!Puc	Bsk	basket	Bsk = Hep ||!Puc	Bsk is activates by Hep and inhibited by Puc	10.3934/genet.2014.1.20, 11402332

AP1	AP	Puc	puckered	Puc = AP	AP1 activates puckered	9472024, 11402332
Bsk	Bsk	Ap	Jun-related antigen	Ap = Bsk	Bsk compete to phosphorylate jun (ap1)	8946915, 11402332
Wts	!Wts	Yki	Yorkie	Yki = !Wts	Wts phosphorylates Yki and inhibits its transcriptional activity	17889654, 21808241
Ral Bsk HpoSal	!Bsk || HpoSal	Wts	warts	Wts = !Bsk || HpoSal	Hippo directly interacts with sav to activate wts [12941273, 21808241]. Wts is inhibited by Bsk [28174264]	12941273, 21808241, 28174264
DILPs	DILPs	InR	Insulin-like receptor	InR = DILPs	ILP1 activates InR1	22252538
InR	InR || Foxo	Chico	chico	Chico = InR || Foxo	Foxo activates Chico and InR activates Chico	30055320
Chico	Chico || Ral	Dp	Pi3K92E Dp110	Dp = Chico || Ral	Chico and Ral activates PI3K92E (Dp110) [30055320, 26119340]	30055320
Dp110	Dp	AKT	AKT1	AKT = Dp	PI3K (Dp110) activates its downstream target AKT [26508828]	26508828
AKT	!AKT	Foxo	forkhead box	Foxo = !AKT	Akt inhibits foxo through direct phosphorylation	21440577
AKT Rolled	AKT || Rolled || tcflef	dMyc	Myc	dMyc = AKT || Rolled || tcflef	AKT and TCFLEF promotes activation of myc [15241468, 31623618]. Rolled (erk) enables myc stabilization [22461507]	15241468, 22461507, 31623618

Supplementary Table 5: Detailed node interaction rules and experimental evidences supporting different interactions and logical functions for Visceral Muscle (VM) cells model.

Visceral Muscles						
**Source Nodes**	**Node Update Logic (TISON)**	**Target Nodes**	**Biological Names**	**Node Update Logic Equation (TISON)**	**Description**	**Reference**
Fz Arm-DECad	!ArmDECad && !Fz	ApcArm	Armadillo B-catenin	ApcArm = !ArmDECad && !Fz	Frizzled is receptor of WNT which activates wingless pathway by inhibting the formation of Apc-Arm. Thereby, Apc cannot degrade Arm. DE-Cad and Apc compete for binding with Arm (B-catenin)[7833051]	16443747, 7833051
Apc-Arm Arm-DECad	!ApcArm && !ArmDECad	Arm	Armadillo B-catenin	Arm = !ApcArm && !ArmDECad	DE-Cad and Apc compete for binding with Arm (B-catenin)[7833051]. Apc inhibits Arm [15169756]. DE-cad inhibit Arm [22174153]	15169756, 7833051, 22174153
Apc-Arm TCF_LEF Snail NICD	!Snail && (!ApcArm || !NICD) && TCFLEF	ArmDECad	Arm *Drosophila* E-Cadherin	ArmDECad = !Snail && (!ApcArm || !NICD) && TCFLEF	Snail which is a known repressor of DE-Cad [15983400]. Arm either binds to cadherin or Apc therefore they inhibit one another. Arm/TCF_LEF complex forms a transcription factor for expression of Arm-DECad (Wingless genes) [18617885, 22174153]. NICD increase so JNK increase [27574001] and JNK increase so its downstream Ecad decrease [25226030, 22174153]	17587826, 16918415, 16720643, 22174153, 15983400, 27574001, 25226030, 22174153
Mad	Mad	Dad	Daughter against dpp	Dad = Mad	Mad induces expression of Dad, a Dpp target gene.	10902180, 9335506

EGFs	EGFs	DER	Torpedo	DER = EGFs	Activated DER receptor activates Ral (fly hmolog of Ras) by phosphorylation. DER is a tyrosine kinase receptor and part of EGF receptor subfamily [1425358]	1425358
Upds	Upds	Dome	Domeless	Dome = Upds	Dome is activated when its ligand Upd binds	24058767
Wg	Wg	Fz	Frizzled	Fz = Wg	Frizzled is receptor of WNT which activates wingless pathway	7833051
Rolled Foxo NICD	!Rolled || !Yki	Hid	Head involution defective Wrinkled	Hid = !Rolled || !Yki	Post-translational modifications of Hid prevent Hid-induced apoptosis [9814704]. For e.g. Rolled inhibts Hid by phosphorylating it [17183370]. Yorkie has anti-apoptotic function which inhibits apoptosis inhibiting gene hid [32485126] Yorkie inhibits bantam and bantem activates hid therefore, yorkie inhibits hid [31331981].	19554451, 17183370, 32485126, 9814704, 28102471, 31331981
Dome Socs36 E	!SocsE && Dome	JAK	Hopscotc h	JAK = !SocsE && Dome	When Upds bind to Dome, JAK get activated via phosphorylation. On the other hand, Socs36E inactivates JAK.	26807580, 19563763
Punt Dad	!Dad && Punt	Mad	Mothers against dpp	Mad = !Dad && Punt	Activated Punt phosphorylate Mad to activate it [24813173]. On the other hand, Dad antagonizes Mad activity [10902180]	24813173, 10902180, 9335506
Notch	Notch	NICD	N^{intra} Cleaved Intracellul ar Domain of Notch	NICD = Notch	Activated Notch undergoes cleavages to form NICD. Wnt inhibits Notch signaling [18806781].	17362357, 20816404, 18806781
Delta	Delta	Notch	Notch	Notch = Delta	Notch is receptor of Delta	7813766

Dpp	Dpp	Punt	Punt	Punt = Dpp	Punt is a receptor of Dpp	21546910
DER Sty	DER && !Sty	Ral	Ras-related protein	Ral = DER && !Sty	Activated DER receptor activates Ral by phosphorylation. Sprouty inhibits the activity of Ras [24530508]	Kegg MAPK - Fly, 24530508
Raf JAK NICD	Raf || JAK || NICD	Rolled	Erk MAP kinase	Rolled = Raf || JAK || NICD	Ral (Ras-related protein in fly) activates Phl (Raf in humans) which goes on to activate Rolled by phosphorylating it [23484853. Both JAK and NICD activate EGFR which activates Rolled. So Rolled gets activated by JAK[8793290, 28472194] and NICD as well [21622856].	Kegg MAPK - Fly, 8793290, 28472194, 21622856
TCF_LEF Rolled NICD	TCFLEF || Rolled || NICD	Snail	Snail	Snail = TCFLEF || Rolled || NICD	Rolled activates Snail [28415812]. TCF is needed for Snail activation [23029025]. NICD also activates Snail [29705809, 15197341]	28415812, 23029025, 29705809, 15197341
Stat92E	StatE	SocsE	Suppressor of cytokine signaling at 36E	SocsE = StatE	Stat92E induces expression of Jak/Stat repressor, Socs36E	19563763
JAK Rolled	JAK || Rolled	StatE	Marelle D-STAT	StatE = JAK || Rolled	JAK promotes activation and dimerization Stat92E. Rolled also activates Stat [27742579]	26807580, 19563763, 18337767, 27742579
Rolled	Rolled	Sty	Sprouty	Sty = Rolled	Rolled activates Pointed which then induces expression of Sprouty	24530508
NICD Mad Stat92E	!Mad && NICD	SuH	Suppressor of Hairless	SuH = !Mad && NICD	NICD promotes activation of Su(H) mediated transcription of Notch genes by removing repression by Hairless [17362357]. Mad inhibits Su(H) activity [23861806, (28945500, inferred)].	28945500, 17362357, 20147375, 26758761, 24077308

					Stat92E promotes expression of Hairless [26758761, 24077308] which acts a repressor of Su(H) [20147375].	
Ral	Ral	Raf	Rapidly Accelerat ed Fibrosarc oma	Raf = Ral	Ras activates Raf [15035987]	15035987
Arm NICD	Arm || NICD	TCFLEF	T-cell factor/ lymphocy te enhancer factor-1	TCFLEF = Arm || NICD	Arm binds to and activates TCF/LEF transcription factors. NICD activates TCFLEF [28245235, 26592459]	Kegg WNT Signaling Fly, 26592459, 28245235
DREF	DREF	Hep	hemipter ous	Hep = DREF	DREF activates Hep which in turn activates bsk	24752236
DREF	DREF	HpoSal	Salvador	HpoSal = DREF	DREF activates Hippo pathway such that it inhibits yki	25424907
Hep Puc	Hep ||!Puc	Bsk	basket	Bsk = Hep ||!Puc	Bsk is activates by Hep and inhibited by Puc	10.3934/genet.2014.1.20, 11402332
AP1	AP	Puc	puckered	Puc = AP	AP1 activates puckered	9472024, 11402332
Bsk	Bsk	Ap	Jun-related antigen	Ap = Bsk	Bsk compete to phosphorylate jun (ap1)	8946915, 11402332
Wts	!Wts	Yki	Yorkie	Yki = !Wts	Wts phosphorylates Yki and inhibits its transcriptional activity	17889654, 21808241
Ral Bsk HpoSal	!Bsk || HpoSal	Wts	warts	Wts = !Bsk || HpoSal	Hippo directly interacts with sav to activate wts [12941273, 21808241]. Wts is inhibited by Bsk [28174264]	12941273, 21808241, 28174264
DILPs	DILPs	InR	Insulin-like receptor	InR = DILPs	ILP1 activates InR1	22252538
InR	InR || Foxo	Chico	chico	Chico = InR || Foxo	Foxo activates Chico and InR activates Chico	30055320

Chico	Chico || Ral	Dp	Pi3K92E Dp110	Dp = Chico || Ral	Chico and Ral activates PI3K92E (Dp110) [30055320, 26119340]	30055320
Dp110	Dp	AKT	AKT1	AKT = Dp	PI3K (Dp110) activates its downstream target AKT [26508828]	26508828
AKT	!AKT	Foxo	forkhead box	Foxo = !AKT	Akt inhibits foxo through direct phosphorylation	21440577
AKT Rolled	AKT || Rolled || tcflef	dMyc	Myc	dMyc = AKT || Rolled || tcflef	AKT and TCFLEF promotes activation of myc [15241468, 31623618]. Rolled (erk) enables myc stabilization [22461507]	15241468, 22461507, 31623618

Supplementary Table 6: Robustness analysis cell fates and corresponding SEMs for ISC, EB, EC and VM.

*EE and EC have the same network so robustness analysis only performed once.

ISC				
Cell fates	MEAN	SD	SEM	SEM (10^-2)
Apoptosis	0.3317	0.0269	0.0006	0.06
Multilayering	0.0858	0.0244	0.0005	0.05
Delta Production	0.0746	0.0200	0.0004	0.04
EB Fate	0.1305	0.0183	0.0004	0.04
Proliferation	0.1305	0.0183	0.0004	0.04
Extrusion	0.1882	0.0126	0.0003	0.03
EE Fate	0.0133	0.0119	0.0003	0.03
Upd Production	0.0458	0.0117	0.0002	0.02
Uncharacterized	0.0114	0.0059	0.0002	0.02

EB				
Cell fates	MEAN	SD	SEM	SEM (10^-2)
Delta Production	0.1032	0.0317	0.0012	0.12
Multilayering	0.0649	0.0266	0.0010	0.10
Extrusion	0.2304	0.0245	0.0009	0.09
EC Fate	0.1331	0.0241	0.0009	0.09
Upd Production	0.0891	0.0214	0.0008	0.08
Apoptosis	0.3794	0.0202	0.0007	0.07
Uncharacterized	0.0036	0.0035	0.0002	0.02

EC				
Cell fates	MEAN	SD	SEM	SEM (10^-2)
Multilayering	0.1075	0.0220	0.0014	0.14
Extrusion	0.1885	0.0200	0.0013	0.13
Dpp Production	0.3311	0.0146	0.0009	0.09
Apoptosis	0.3311	0.0146	0.0009	0.09
Upd Production	0.0420	0.0133	0.0009	0.09

VM				
Cell fates	MEAN	SD	SEM	SEM (10^-2)
WNT target genes	0.1735	0.0238	0.0009	0.09
Apoptosis	0.3983	0.0143	0.0005	0.05
Dpp Production	0.3983	0.0143	0.0005	0.05
Upd Production	0.0302	0.0108	0.0004	0.04

Supplementary Table 7: Input node states in normal, stress and cancer for ISC's Apical region, ISC's Basal region, EB and EC network models and their literature validation.

Literature on Modelled Pathways			
Sr #	Pathways	State in Cancer	PubMed ID
1	EGFR "EGFs"	↑	16034367
2	WNT "Wg"	↑	16034367
3	JAK-STAT "Upds"	↑↓	16034367/24516653
4	Notch "Delta"	↑↓	16034367/(22863622, 18758480)
5	BMP "Dpp"	↑	16034367
6	Robo "Slit"	↑	24931602
7	Insulin "dILPs"	↑	29793481
8	JNK "DREF"	↑	32513656
9	Hippo "DREF"	↑	20727758

↑	Upregulated
↓	Downregulated
↑↓	Mixed reports (Up & Downregulation)

Inputs for Each Pathway									
Cells	Inputs	EGFs	Wg	Upds	Dpp	Delta	Slit	Dref	Dilps
ISC_Apical	Normal	0.80	0.30	0.40	0.40	0.60	0.40	0.70	0.00
	Stress	0.80	0.30	0.40	0.00	0.60	0.90	0.70	0.00
	Cancer	0.90	0.90	0.50	0.80	0.50	0.90	0.90	0.90
ISC_Basal	Normal	0.50	0.20	0.70	0.30	0.20	0.01	0.70	0.00
	Stress	0.00	0.30	0.20	0.10	0.30	0.20	0.30	1.00
	Cancer	1.00	1.00	1.00	1.00	0.10	0.50	0.90	0.90
Enteroblast	Normal	0.90	0.20	0.60	0.40	0.70	N/A	0.70	0.00
	Stress	0.60	0.30	0.60	0.50	0.50	N/A	0.80	0.80
	Cancer	1.00	0.90	0.40	1.00	0.40	N/A	0.90	0.90
Enterocyte	Normal	0.80	0.40	N/A	0.30	0.60	N/A	0.70	0.00
	Stress	0.40	0.30	N/A	0.40	0.20	N/A	0.80	0.80
	Cancer	0.90	0.90	N/A	0.80	0.01	N/A	0.90	0.90

Supplementary Table 8: Cell fate propensities of Intestinal Stem Cells (ISC) in apical and basal compartments; Enteroblast (EB) and Enterocytes (EC) in normal, stress and cancer conditions along with literature validations.

Intestinal Stem Cell (ISC)'s Apical Region				
**Cell Fate**	**Normal condition**	**Stress condition**	**Cancer condition**	**Ref.**
EB Fate	0.130	0.141	0.089	[2]
Proliferation	0.130	0.141	0.089	[4, 12]
Upd Production	0.046	0.000	0.068	[8]
Delta Production	0.062	0.074	0.014	[5]
Apoptosis	0.295	0.344	0.307	[7, 13]
Multilayering	0.077	0.087	0.270	[6]
Uncharacterized	0.000	0.000	0.000	[11]
EE Fate	0.081	0.013	0.016	NA
Extrusion	0.178	0.200	0.148	[9]
**Intestinal Stem Cell (ISC)'s Basal Region**				
**Cell Fate**	**Normal condition**	**Stress condition**	**Cancer condition**	**Ref.**
EE Fate	0.303	0.214	0.138	[2]
Upd Production	0.012	0.006	0.017	[8]
Delta Production	0.089	0.102	0.000	[5]
Apoptosis	0.353	0.375	0.353	[7, 13]
Multilayering	0.058	0.095	0.353	[6]
Proliferation	0.045	0.069	0.017	[4, 12]
EB Fate	0.045	0.069	0.017	NA
Uncharacterized	0.000	0.000	0.000	[11]
Extrusion	0.094	0.069	0.106	[9]
**Enteroblast**				
**Cell Fate**	**Normal condition**	**Stress condition**	**Cancer condition**	**Ref.**
Upd Production	0.088	0.073	0.103	[8]
Delta Production	0.105	0.120	0.027	[5]
Apoptosis	0.381	0.450	0.394	[7, 13]
Multilayering	0.064	0.109	0.351	[6]
Uncharacterized	0.000	0.000	0.000	[11]
EC Fate	0.133	0.080	0.000	[2]
Extrusion	0.229	0.166	0.124	[9]
**Enterocyte**				
**Cell Fate**	**Normal condition**	**Stress condition**	**Cancer condition**	**Ref.**
Dpp Production	0.331	0.406	0.331	[10]
Apoptosis	0.331	0.406	0.331	[7, 13]
Extrusion	0.189	0.078	0.052	[9]
Multilayering	0.107	0.092	0.284	[6]
Upd Production	0.041	0.019	0.002	[8]

Supplementary Table 9: A comparison of model and experimental output node propensities.

*Experimental values taken from FlyGut-seq database

Cell Types	Normal Input Nodes	Normal Input Nodes Propensities From Database	Normal Output Nodes	Experimental	Model	Error
Intestinal Stem Cell: Apical	EGFs	0.80	apcarm	0.70	0.70	0.00
	Wg	0.30	cdc	0.60	0.80	0.20
	Upds	0.40	hid	0.70	0.90	0.20
	Dpp	0.40	suh	0.70	0.50	-0.20
	Delta	0.60	pros	0.30	0.30	0.00
	Slit	0.40	dlg	0.70	0.80	0.10
	DREF	0.70	state	0.80	0.90	0.10
	DILPS	0.00	rolled	0.80	0.80	0.00
			tcflef	0.80	0.70	-0.10
			dmyc	0.70	0.90	0.20
Intestinal Stem Cell: Basal	N/A					
Enteroblast	EGFs	0.90	apcarm	0.80	0.80	0.00
	Wg	0.20	cdc	0.80	0.90	-0.10
	Upds	0.60	dlg	0.90	0.90	0.00
	Dpp	0.40	hid	0.70	0.80	-0.10
	Delta	0.70	rolled	0.90	0.80	0.10
	DREF	0.60	state	0.80	0.90	-0.10
	DILPS	0.00	suh	0.70	0.60	0.10
			tcflef	0.90	0.80	0.10
			dmyc	0.60	0.90	-0.30
Enterocyte	EGFs	0.80	apcarm	0.80	0.60	0.20
	Wg	0.40	cdc	0.60	0.80	-0.20
	Dpp	0.30	dlg	0.70	0.80	-0.10
	Delta	0.60	hid	0.70	0.90	-0.20
	DREF	0.70	rolled	0.70	0.80	-0.10
	DILPS	0.00	suh	0.60	0.50	0.10
			tcflef	0.80	0.80	0.00
			dmyc	0.70	0.90	-0.20

Supplementary Table 10: Tabulation of network nodes, gene IDs, annotation symbols, gene symbols, and FlyBase genes.

Network Names	GeneID	Annotation Symbol	Fly Gene Symbol	Gene Name in Flybase DataBase	Human Gene Homologs from Genecards
AKT	FBgn0010379	CG4006	Dmel\Akt	Akt1	AKT
Ap	FBgn0001291	CG2275	Dmel\Jra	Jun-related antigen	JUN
apcarm	FBgn0026598	CG6193	Dmel\Apc2	Adenomatous polyposis coli	APC
arm	FBgn0000117	CG11579	Dmel\arm	armadillo	CTNNB1
armdecad	FBgn0015609	CG7100	Dmel\CadN	Cadherin-N	CDH
Bsk	FBgn0000229	CG5680	Dmel\bsk	basket	TPR
cdc	FBgn0010341	CG12530	Dmel\Cdc42	Cdc42	CDC
Chico	FBgn0024248	CG5686	Dmel\chico	Chico	TLE
dad	FBgn0020493	CG5201	Dmel\Dad	Daughters against dpp	NA
delta	FBgn0000463	CG3619	Dmel\Dl	Delta	DLL
der	FBgn0003731	CG10079	Dmel\Egfr	Epidermal growth factor receptor	EGFR
DILPS	FBgn0044051	CG14173	Dmel\Ilp1	Insulin-like peptide 1	BIRC
dlg	FBgn0001624	CG1725	Dmel\dlg1	discs large 1	DLG
dMyc	FBgn0262656	CG10798	Dmel\Myc	diminutive	MYC
dome	FBgn0043903	CG14226	Dmel\dome	domeless	NA
Dp	FBgn0015279	CG4141	Pi3K92E	Dmel\Pi3K92E	PIK3CA
dpp	FBgn0000490	CG9885	Dmel\dpp	decapentaplegic	SMAD
DREF	FBgn0015664	CG5838	Dmel\Dref	DNA replication-related element factor	ZBED
egfs	FBgn0005672	CG10334	Dmel\spi	spitz	EGF
Foxo	FBgn0038197	CG3143	forkhead box, sub-group O	Dmel\foxo	FOXO

**fz**	**FBgn0001085**	**CG17697**	**Dmel\fz**	**frizzled**	**FZD**
**Hep**	**FBgn0010303**	**CG4353**	**Dmel\hep**	**hemipterous**	**MAP2K7**
**hid**	**FBgn0003997**	**CG5123**	**Dmel\hid**	**Wrinkled**	**NA**
**HpoSal**	**FBgn0053193**	**CG33193**	**Dmel\sav**	**salvador**	**STK4**
**InR**	**FBgn0013984**	**CG18402**	**Dmel\InR**	**Insulin-like receptor**	**IRS**
**jak**	**FBgn0004864**	**CG1594**	**Dmel\hop**	**hopscotch**	**JAK**
**mad**	**FBgn0011648**	**CG12399**	**Dmel\Mad**	**Mothers against dpp**	**SMAD**
**mir**	**FBgn0014343**	**CG10601**	**Dmel\mirr**	**mirror**	**NA**
**nicd**	**NA**				**NOTCH**
**notch**	**FBgn0004647**	**CG3936**	**Dmel\N**	**Notch**	**NA**
**pros**	**FBgn0004595**	**CG17228**	**Dmel\pros**	**prospero**	**PROS**
**Puc**	**FBgn0243512**	**CG7850**	**Dmel\puc**	**puckered**	**NA**
**punt**	**FBgn0003169**	**CG7904**	**Dmel\put**	**punt**	**ACVR**
**Raf**	**FBgn0003079**	**CG2845**	**Dmel\Raf**	**pole hole**	**RAF**
**ral**	**FBgn0026056**	**CG11622**	**Dmel\Rlip**	**Ral interacting protein**	**RASA/KRAS**
**robo**	**FBgn0041097**	**CG5423**	**Dmel\robo3**	**roundabout 3**	**ROBO**
**rolled**	**FBgn0003256**	**CG12559**	**Dmel\rl**	**rolled**	**MAPK**
**slit**	**FBgn0264089**	**CG43758**	**Dmel\sli**	**slit**	**SLIT**
**snail**	**FBgn0003448**	**CG3956**	**Dmel\sna**	**snail**	**SNAI1**
**socse**	**FBgn0041184**	**CG15154**	**Dmel\Socs36E**	**Suppressor of cytokine signaling at 36E**	**SOCS**
**state**	**FBgn0016917**	**CG4257**	**Dmel\Stat92E**	**Signal-transducer and activator of transcription protein at 92E**	**STAT**
**sty**	**FBgn0014388**	**CG1921**	**Dmel\sty**	**sprouty**	**SPRY**
**suh**	**FBgn0004837**	**CG3497**	**Dmel\Su(H)**	**Suppressor of Hairless**	**RBP**

**tcflef**	**FBgn0085432**	**CG34403**	**Dmel\pan**	**pangolin**	**TCF**
**upds**	**FBgn0030904**	**CG5988**	**Dmel\upd2**	**unpaired 2**	**NA**
**wg**	**FBgn0284084**	**CG4889**	**Dmel\wg**	**wingless**	**WNT**
**Wts**	**FBgn0011739**	**CG12072**	**Dmel\wts**	**warts**	**LATS1**
**Yki**	**FBgn0034970**	**CG4005**	**Dmel\yki**	**yorkie**	**YAP1**

Supplementary Table 11: Martorell et al.'s predictions: experiment versus model.

*Data collected from http://hdl.handle.net/2445/55144 and 24516653

Intestinal Stem Cell			
Martorell *et al.*'s Predictions - Experiment			
Cell Fates	APC	RAS	APC+RAS
Apoptosis			
Proliferation (EB/EE)	Apc clones are Hyperproliferative,	Hyperproliferation	Increase proliferation rate
Differentiation	Differentiation is almost same as wild type	Differentiation is almost same as wild type	Decrease differentiation rate
Loss of Polarity	No mention	Loss of cell polarity observed in the midgut	Loss of polarity observed
Multi-layering	No mention	No mention	Multi-layering observed
Extrusion	No mention	Cell extrusion in the adult midgut.	Extrusion observed

Intestinal Stem Cell				
Model Results				
Cell Fates	Control	APC	RAS	APC + RAS
Apoptosis	0.296	0.256	0.210	0.173
Proliferation	0.130	0.112	0.148	0.173
Differentiation (EB Fate)	0.130	0.112	0.130	0.112
Loss of Polarity	0.000	0.000	0.080	0.061
Multi-layering	0.077	0.256	0.052	0.173
Extrusion	0.179	0.151	0.210	0.173

Supplementary Table 12: Differential gene expression comparison between prediction and the model.

*DGE compared with doctorial thesis results, available here: http://hdl.handle.net/2445/55144

Intestinal Stem Cell	
Martorell et al.'s Predictions - Experiment	
Nodes	APC+RAS
arm	Upregulated
armdecad	downregulated
cdc	Upregulated
dad	downregulated
der	downregulated
dlg	downregulated
fz	Upregulated
jak	downregulated
mad	downregulated
mir	Upregulated
pros	marker for ee - show heterogeneity
punt	downregulated
snail	Upregulated
sty	Upregulated
suh	marker for eb - show heterogeneity
tcflef	Upregulated
hposal	Upregulated
Ap	Upregulated
yki	downregulated
inr	Upregulated
dp110	Upregulated
dmyc	Upregulated

Intestinal Stem Cell		
Model Results		
Nodes	Control	APC + RAS
apcarm	0.698	0.000
arm	0.301	1.000
armdecad	0.004	0.000
cdc	0.848	1.000
dad	0.200	0.200

der	0.800	0.800
dlg	0.831	0.600
dome	0.400	0.400
fz	0.300	0.300
hid	0.885	0.850
jak	0.035	0.000
mad	0.200	0.200
mir	0.839	1.000
nicd	0.600	0.600
notch	0.600	0.600
pros	0.314	0.313
punt	0.400	0.400
ral	0.161	1.000
robo	0.400	0.400
rolled	0.769	1.000
snail	0.839	1.000
socse	0.886	1.000
state	0.886	1.000
sty	0.769	1.000
suh	0.481	0.482
raf	0.161	1.000
tcflef	0.722	1.000
hep	0.700	0.700
hposal	0.700	0.700
bsk	0.850	0.850
puc	0.850	0.850
ap	0.850	0.850
yki	0.150	0.150
wts	0.850	0.850
inr	0.000	0.000
chico	0.500	0.000
dp	0.500	1.000
akt	0.500	1.000
foxo	0.500	0.000
dmyc	0.863	1.000
egfs	0.800	0.800
upds	0.400	0.400
wg	0.300	0.300
delta	0.600	0.600

dpp	0.400	0.400
slit	0.400	0.400
dref	0.700	0.700
dilps	0.000	0.000

Nodes	Status
egfs	Input Nodes
upds	Input Nodes
wg	Input Nodes
delta	Input Nodes
dpp	Input Nodes
slit	Input Nodes
dref	Input Nodes
dilps	Input Nodes
apcarm	Mutated
ral	Mutated
hid	Not mentioned
nicd	Not mentioned
dome	Not mentioned
robo	Not mentioned
notch	Not mentioned
rolled	Not mentioned
Raf	Not mentioned
socse	Not mentioned
hep	Not mentioned
bsk	Not mentioned
puc	Not mentioned
wts	Not mentioned
chico	Not mentioned
akt	Not mentioned
foxo	Not mentioned
state	Not Validated
armdecad	Validated
cdc	Validated
dad	Not affected
dlg	Validated
mad	Not affected
mir	Validated
pros	Not affected
suh	Not affected
tcflef	Validated
arm	Validated
punt	Not affected
snail	Validated
der	Not affected
fz	Not affected
jak	Validated
sty	Validated
hposal	Not affected
ap	Not affected
yki	Not affected
inr	Not affected
dp	Validated
dmyc	Validated

Supplementary Table 13: Results of class I and class II drugs from Markstein *et al.*'s therapeutics screens.

Markstein Drosophila Drug Screening									
Class	Drug	Mechanism of Action with Direct Target	Direct Targets Literature/Databases	Representative Targets from Database	Database Link	in Direct Targets	Description of indirect interaction	Therapy in TISON	Reference
Class I	Thiotepa	inhibition	DNA [DrugBank]	p53 [DGIdb]	http://www.dgidb.org/	Arm	The drug directly increases p53 levels [DGIdb, 12058967, 9548807]. And p53 inhibts Arm (Armadillo, homolog for beta catenin in Drosophila) [11564862, 27908728]	Arm -> 0	11564862, 9548807, 12058967, 27908728
	Methotrexate	inhibition	Dihydrofolate reductase [DrugBank]	DHFR [DGIdb]	http://www.dgidb.org/	p53	The drug is an inhibitor of DHFR [DGIdb]. Downregulation of DHFR increases p53 levels [27013776] and p53 inhinits arm [11564862, 27908728]	Arm -> 0	27013776, 11564862, 27908728
	Floxuridine	inhibition	Thymidylate synthase [DrugBank]	PIK3CA [PanDrugs]	https://www.pandrugs.org/#!/query/	p53	The drug is an inhibitor of PI3K [PanDrugs, 25714871], PI3K activates c-myc which inhibits p53 [30944308]. So when PI3K levels are decreased via drug, p53 increases which inhibits arm [11564862, 27908728]	Arm -> 0	PanDrugs, 30944308, 11564862, 27908728
	Topotecan	inhibition	DNA topoisomerase 1 [DrugBank]	PIK3CA [PanDrugs]	https://www.pandrugs.org/#!/query/	p53	PI3K activates c-myc which inhibts p53 [30944308]. So when PI3K levels are decreased via drug, p53 increases which inhibits arm [11564862, 27908728]	Arm -> 0	30944308, 11564862, 27908728, 11857382
	Rapamycin/Sirolimus	inhibition	Serine/threonine-protein kinase mTOR [DrugBank]	mtor [DrugBank]	https://www.drugbank.ca/drugs/DB00877	p53	mtor activates mdm2 which inhibts p53 so when mtor is inhibited via drug so p53 is stabilized [17329361] which means arm is inhibted [11564862, 27908728]	Arm -> 0	17329361, 11564862, 27908728

Markstein Drosophila Drug Screening									
Class	Drug	Mechanism of Action with Direct Target	Direct Targets Literature/Databases	Representative Targets from Database	Database Link	in Direct Targets	Description of indirect interaction	Therapy in TISON	Reference
Class II	Bortezomib	inhibition	Proteasome subunit beta type-5 [DrugBank]	Beta-catenin [22726547] Ras [DGIdb]	http://www.dgidb.org/	N/A	Bortezomib activates beta catenin pathway [22726547]. Ras cell more susceptiable to bortezomib.	arm -> 1 ral -> 0	22726547, 16778179
	Paclitaxel	inhibition	Tubulin beta-1 chain [DrugBank]	Ras [DGIdb]	http://www.dgidb.org/	catenin Snail ArmDECad	Paclitaxel inhibits ras [24212635]. Wnt signaling increased after paclitaxel treatment, beta-catenin expression was increased [31687397]. Snail expression is increased in paclitaxel [26462028]. Reduced expression of ecadherin [25330011]	arm -> 1 ral -> 0 Snail -> 1 ArmDECad -> 0	24212635, 31687397, 26462028, 25330011
	Vinblastine	inhibition	Tubulin alpha-1A chain [DrugBank]	Ras [23564313]	N/A	catenin	vinblastine (a vinca alkaloids) reduced ERK expression, [7270426] (inferred). Cell resisting apoptosis when injected with vinblastine have increased wnt signaling and wnt signaling is dependent on bet catenin activation [11149923]	arm -> 1 ral -> 0	7270426, 11149923
	Vincristine	inhibition	Tubulin beta chain [DrugBank]	Ras [19247395]	N/A	catenin	VCR can downregulate Ras [19247395]. Cell resisting apoptosis when injected with vinblastine have increased wnt signaling and wnt signaling is dependent on bet catenin activation [11149923] (inferred)	arm -> 1 ral -> 0	19247395, 11149923

Supplementary Table 14: Cell fate propensities for proliferation and apoptosis in class I and class II drugs.

*For same drug target same propensities remain the same as well

Class I												
Cell Fates	Control		Thiotepa		Methotrexate		Floxuridine		Topotecan		Rapamycin	
	WT	Raf mutation	WT	Raf mutation	WT	Raf mutation	WT	Raf mutation	WT	Raf mutation	WT	Raf mutation
Apoptosis	0.286	0.175	0.283	0.263	0.283	0.263	0.283	0.263	0.283	0.263	0.283	0.263
Proliferation	0.157	0.162	0.130	0.089	0.130	0.089	0.130	0.089	0.130	0.089	0.130	0.089

Class II										
Cell Fates	Control		Bortezomib		Paclitaxel		Vinblastine		Vincristine	
	WT	Raf mutation	WT	Raf mutation	WT	Raf mutation	WT	Raf mutation	WT	Raf mutation
Apoptosis	0.286	0.175	0.336	0.306	0.329	0.308	0.336	0.306	0.336	0.306
Proliferation	0.157	0.162	0.188	0.175	0.191	0.172	0.188	0.175	0.188	0.175

Supplementary Table 15: Details of the Bangi et al.'s case study: mutations, therapy, and induction of therapy in the in silico DPM.

Bangi *et al's* Study Details														
Mutation	Type	Fly ortholog	Type of mutations	In literature	In model	Presence	Indirect mechanism of action	Mutation induction	Therapy	In literature	In Model	Presence	Indirect mechanism of action	Therapy induction
KRAS	oncogenic	ras85D	Oncogene	ras	Ral	Direct	N/A	Ral -> 1	Trametinib	MEK	Rolled	Indirect	MEK activates ERK (Rolled in the model)	Rolled -> 0
APC	biallelic loss	apc	Tumor Suppressor	apc	apcarm	Direct	N/A	Apcarm -> 0	Zoledronate	Ras	Ral	Indirect	Ras is inhibited using Zoledronate [30882399]	Ral -> 0
TP53	biallelic loss	p53	Tumor Suppressor	p53	arm	Indirect	p53 inhibts arm [11564862, 27908728]	arm -> 1						
FBXW7	biallelic loss	ago	Tumor Suppressor	ago	Notch	Indirect	FBXW7 inhibits Notch [s12943-018-0857-2]	Notch -> 1						
TGFBR2	germline heterozygous missense mutation	put	Tumor Suppressor	punt	punt	Direct	N/A	punt -> 0						
SMARCA4	heterozygous somatic mutations	brm	Tumor Suppressor	brm	arm	Indirect	brm downregulation handicaps p53 [31722744] and p53 inhibits arm [11564862, 27908728]	arm -> 1						
FAT4	heterozygous somatic mutations	ft	Tumor Suppressor	fat	fz	Indirect	fat activates fz [15923647]. Exact regulation is unknown	fz -> 0						
MAPK14	heterozygous somatic mutations	p38a	Tumor Suppressor	Ras	Raf	Direct	p38 stress suppresses ras dependent growth. So it inhibits ras [18508457]	Raf -> 1						
CDH1	heterozygous germline mutation	shg	Tumor Suppressor	chd1	armdecad	Direct	N/A	armdecad -> 0						

Supplementary Table 16: Patient mutations from cBioPortal for genes in our network

Patients ID's from cBioPortal	Generic		Patient ID: 01CO001		Patient ID: 32904		Patient ID: 11CO059		Patient ID: 12915		Patient ID: 32908	
	cBioPortal	Network Nodes	cBioPortal	Network Nodes	cBioPortal	Network Nodes	cBioPortal	Network Nodes	cBioPortal	Network Nodes	cBioPortal	Network Nodes
Mutations	APC	apcarm	CDH23	armdecad	SMAD4	dad/mad	DLGAP3	dlg	DLGAP2	dlg	DLG1	dlg
	RAS	ral	SMAD4	dad/mad	CDC14A	cdc	DLGAP2		DLG5		DLG2	
			APC	apcarm	KRAS	ral	DLG5		DLGAP4		DLG4	
			STK31	HpoSal			DLG5		DLGAP3		DLGAP2	
							DLGAP2		CDH1	armdecad	DLG5	
							DLGAP2		CDH15		DLGAP5	
							CDH8	armdecad	SMAD7	dad/mad	DLGAP4	
							CDH23		APC	apcarm	CDH11	armdecad
							JAK2	jak	SLIT2	slit	CDH2	
							SMAD1	dad/mad	WNT3	wg	CDH6	
							ACVR2A	punt	NOTCH1	notch	CDH8	
							ACVR1C		NOTCH3		CDH9	
							RBPJL	suh	FZD10	fz	JAK3	jak
							RBP1		TCF3	tcflef	SMAD2	dad/mad
							NRBP2		TCF15		SMAD4	
							APC	apcarm	ROBO2	robo	SMAD1	
							APC2		RASAL2	ral	SMAD9	
							MEGF8	egfs	DLL1	delta	ACVR2B	punt
							MAPK4	rolled	ARAF	raf	RBP3	suh
							MAPK11		BIRC6	dilps	APC	apcarm
							MAPK15		TLE2	chico	EGFR	der
							SLIT2	slit	TLE4		EGF	egfs
							WNT1	wg	PIK3CA	dp	MAPK6	rolled
							NOTCH3	notch	MYCBP2	dmyc	SLIT1	slit
							CTNNBL1	arm			WNT2	wg
							FZD6	fz			WNT5A	
							FZD7				WNT7B	
							FZD9				WNT8A	
							SPRY4	sty			WNT8B	
							TCF15	tcflef			WNT10B	
							TCF20				WNT2B	
							ROBO1	robo			NOTCH3	notch
							ROBO3				FZD1	fz
							STAT2	stat			FZD6	
							CDC27	cdc			FZD9	
							CDC42BPA				TCF7L2	tcflef
							CDC42BPG				ROBO1	robo
							CDC42BPG				ROBO2	
							RASA3	ral			ROBO3	
							RASA2				STAT1	state
							SOCS4	socs			STAT2	
							SOCS5				STAT3	
							STK11	HpoSal			STAT4	
							STK32C				CDC73	cdc
							STK11IP				CDC25A	
							STK26				CDC25C	
							JUNB	ap			CDC45	
							JUND				CDC14B	
							IRS1	inr			CDC23	
							IRS2				FRAS1	ral
							TLE2	chico			RASA1	
							TLE6				RASA2	
							AKT3	akt			SOCS4	socse
							FOXO1	foxo			SOCS5	
							MYC	dmyc			BIRC2	dilps
							PIK3R3	dp			BIRC6	
							PIK3R1				MAP2K1	Hep
							PIK3R1				MAP2K5	
							PIK3CG				STK10	HpoSal
							PIK3R1				STK31	
							PIK3R6				IRS1	inr
							PIK3R6				TLE2	chico
							PIK3R4				TLE4	
											TLE6	
											PIK3CA	dp
											AKT3	akt
											MYCT1	myc
											MYCBP2	

Patient ID: 05CO044		Patient ID: 32824		Patient ID: 32360		Patient ID: 05CO015		Patient ID: 32832	
cBioPortal	Network Nodes	cBioPortal	Network Nodes	cBioPortal	Network Nodes	cBioPortal	Network Nodes	cBioPortal	Network Nodes
DLGAP1	dlg	CDH8	armdecad	DLGAP1	dlg	DLGAP5	dlg	CDH11	armdecad
DLGAP2		SMAD4	dad/mad	DLGAP2		CDH1	armdecad	CDH24	
DLGAP3		STAT5A	stat	DLGAP3		CDH23		CDHR1	
DLGAP4		KRAS	ral	CDH4	armdecad	ACVR2A	punt	APC	apcarm
CDH13	armdecad	PIK3CA	dp	CDH5		ACVR2B		MAPK15	rolled
CDH23				CDH7		APC	apcarm	WNT16	wg
JAK1	jak			CDH20		APC2		CTNNBL1	arm
ACVR2A	punt			JAK3	jak	MAPKAP1	rolled	FZD10	fz
ACVR2B				SMAD2	dad/mad	SLIT1	slit	TCF25	tcflef
ACVR1C				SMAD9		SLIT2		ROBO1	robo
APC2	apcarm			ACVR2A	punt	SLIT3		STAT6	stat
EGF	egfs			RBP3	suh	WNT16	wg	CDC23	cdc
MAPK12	rolled			APC	apcarm	WNT2B		RASIP1	ral
MAPK15				APC2		TCF25	tcflef	MAP2K7	Hep
SLIT2	slit			EGF	egfs	TCF20		JUNB	ap
WNT2	wg			MAPK12	rolled	ROBO2	robo	IRS2	inr
WNT2B				SLIT1	slit	CDC7	cdc		
WNT7B				SLIT2		CDC23			
NOTCH2	notch			SLIT3		RRAS2	ral		
NOTCH3				WNT8A	wg	RASGRF2			
FZD2	fz			WNT16		RASA1			
FZD5				WNT10A		FRAS1			
SPRYD4	sty			FZD9	fz	BRAF	raf		
SPRYD7				ROBO3	robo	ZBED6	dref		
TCF7L2	tcflef			STAT4	stat	STK38L	HpoSal		
ROBO3	robo			STAT5A		STK11IP			
ROBO1				STAT6		IRS1	inr		
CDC42BPA	cdc			CDCA2	cdc	IRS4			
RASAL2	ral			KRAS	ral	TLE4	chico		
NRAS				RASA1		PIK3CB	dp		
FRAS1				RASA3		PIK3R3			
ARAF	raf			FRAS1		FOXO1	foxo		
SOCS2	socse			BRAF	raf	MYCN	dmyc		
ZBED4	dref			SOCS6	socse				
STK33	HpoSal			ZBED6CL	dref				
STK11				MAP2	Hep				
STK10				STK38	HpoSal				
JUND	ap			LATS2	Wtz				
TLE2	chico			IRS1	inr				
TLE3				IRS2					
TLE4				IRS4					
PIK3CG	dp			PIK3C2B	dp				
PIK3C2G				PIK3CB					
PIK3C3				PIK3C2A					
PIK3CA				PIK3AP1					
PIK3CD									
FOXO4	foxo								
MYCBP2	dmyc								

Supplementary Table 17: Oncogenic cell fate propensities of potential target nodes, highlighted in blue (pro-apoptotic) and green (anti-proliferation).

Patients ID's from cBioPortal	Cell Fates	Control	Mutated	Human Gene IDs	DLG	CDH		JAK	SMAD	ACVR	RBP		APC	EGFR	EGF	MAPK	SMAD	SLIT	WNT	PROS	NOTCH	CTNNB1	FZD
				Network Node Names	dlg	armdecad	dad	jak	mad	punt	suh	hid	apcarm	der	egfs	rolled	dpp	slit	wg	pros	nicd	arm	fz
Generic	Proliferation	0.129	0.173		0.130	0.112	0.142	0.147	0.125	0.114	0.141	0.130	0.138	0.131	0.130	0.000	0.142	0.130	0.140	0.131	0.000	0.130	0.138
	Apoptosis	0.294	0.173		0.298	0.232	0.309	0.213	0.292	0.283	0.321	0.295	0.300	0.288	0.324	0.409	0.295	0.261	0.308	0.329	0.385	0.288	0.305
Patient ID: 01CO001	Proliferation	0.130	0.120		0.132	0.112	0.141	0.149	0.126	0.114	0.143	0.129	0.139	0.130	0.130	0.000	0.141	0.130	0.138	0.130	0.000	0.131	0.138
	Apoptosis	0.291	0.265		0.298	0.226	0.309	0.210	0.296	0.279	0.307	0.297	0.303	0.277	0.336	0.419	0.302	0.260	0.307	0.328	0.412	0.284	0.317
Patient ID: 32904	Proliferation	0.132	0.164		0.129	0.112	0.141	0.150	0.124	0.114	0.141	0.129	0.137	0.130	0.129	0.000	0.141	0.130	0.138	0.130	0.000	0.132	0.139
	Apoptosis	0.294	0.221		0.292	0.231	0.307	0.209	0.292	0.283	0.321	0.295	0.302	0.288	0.311	0.415	0.294	0.259	0.313	0.325	0.388	0.287	0.306
Patient ID: 11CO059	Proliferation	0.130	0.167		0.130	0.113	0.141	0.145	0.125	0.114	0.141	0.129	0.138	0.129	0.132	0.000	0.142	0.130	0.139	0.130	0.000	0.129	0.137
	Apoptosis	0.295	0.167		0.293	0.229	0.306	0.212	0.292	0.283	0.321	0.293	0.309	0.286	0.325	0.381	0.298	0.261	0.325	0.328	0.388	0.291	0.305
Patient ID: 12915	Proliferation	0.131	0.167		0.133	0.113	0.141	0.150	0.127	0.115	0.141	0.130	0.138	0.131	0.132	0.000	0.141	0.130	0.139	0.130	0.000	0.129	0.138
	Apoptosis	0.294	0.167		0.303	0.227	0.307	0.209	0.285	0.286	0.319	0.299	0.309	0.291	0.323	0.390	0.296	0.260	0.307	0.327	0.392	0.283	0.299
Patient ID: 32908	Proliferation	0.130	0.166		0.130	0.112	0.140	0.149	0.125	0.114	0.141	0.132	0.138	0.130	0.131	0.000	0.140	0.130	0.138	0.132	0.000	0.131	0.138
	Apoptosis	0.301	0.166		0.290	0.226	0.304	0.210	0.298	0.283	0.320	0.300	0.311	0.288	0.319	0.385	0.294	0.260	0.315	0.320	0.391	0.291	0.308
Patient ID: 05CO044	Proliferation	0.131	0.167		0.132	0.111	0.142	0.146	0.125	0.115	0.142	0.131	0.140	0.130	0.132	0.000	0.142	0.130	0.137	0.131	0.000	0.129	0.138
	Apoptosis	0.291	0.167		0.296	0.226	0.308	0.213	0.295	0.292	0.319	0.301	0.312	0.289	0.314	0.383	0.304	0.258	0.312	0.329	0.381	0.281	0.308
Patient ID: 32824	Proliferation	0.131	0.163		0.130	0.113	0.141	0.149	0.125	0.114	0.141	0.132	0.139	0.131	0.129	0.000	0.142	0.130	0.139	0.131	0.000	0.132	0.138
	Apoptosis	0.293	0.220		0.305	0.229	0.308	0.210	0.290	0.285	0.321	0.298	0.304	0.290	0.328	0.418	0.296	0.263	0.305	0.321	0.389	0.285	0.310
Patient ID: 32360	Proliferation	0.130	0.167		0.130	0.112	0.141	0.148	0.125	0.115	0.142	0.131	0.141	0.130	0.131	0.000	0.141	0.131	0.137	0.131	0.000	0.129	0.138
	Apoptosis	0.302	0.168		0.304	0.230	0.311	0.209	0.288	0.282	0.319	0.294	0.305	0.294	0.317	0.415	0.299	0.258	0.300	0.332	0.387	0.289	0.308
Patient ID: 05CO015	Proliferation	0.131	0.167		0.132	0.113	0.142	0.149	0.126	0.115	0.141	0.130	0.138	0.131	0.132	0.000	0.141	0.131	0.138	0.131	0.000	0.129	0.137
	Apoptosis	0.297	0.167		0.295	0.228	0.310	0.211	0.293	0.283	0.320	0.295	0.309	0.292	0.318	0.413	0.296	0.256	0.309	0.323	0.388	0.288	0.306
Patient ID: 32832	Proliferation	0.130	0.178		0.129	0.112	0.141	0.148	0.125	0.114	0.142	0.130	0.138	0.130	0.129	0.000	0.141	0.131	0.138	0.131	0.000	0.129	0.138
	Apoptosis	0.299	0.178		0.307	0.229	0.311	0.210	0.290	0.284	0.320	0.296	0.306	0.291	0.317	0.427	0.299	0.257	0.313	0.329	0.394	0.284	0.305

Category A	Pro-Apoptotic
Category B	Anti-Proliferation

Patients ID's from cBioPortal	Cell Fates	Control	Mutated	Human Gene IDs		SNAI	SPRY	TCF	ROBO		STAT	CDC	RASA/KRAS	DLL			RAF
				Network Node Names	mir	snail	sty	tcflef	robo	notch	state	cdc	ral	delta	upds	dome	Raf
Generic	Proliferation	0.129	0.173		0.131	0.131	0.144	0.000	0.130	0.000	0.000	0.166	0.130	0.000	0.131	0.130	0.131
	Apoptosis	0.294	0.173		0.243	0.348	0.235	0.317	0.265	0.415	0.341	0.334	0.365	0.412	0.299	0.301	0.374
Patient ID: 01CO001	Proliferation	0.130	0.120		0.129	0.130	0.147	0.000	0.130	0.000	0.000	0.167	0.131	0.000	0.130	0.130	0.129
	Apoptosis	0.291	0.265		0.244	0.380	0.234	0.323	0.266	0.412	0.308	0.402	0.383	0.413	0.292	0.291	0.383
Patient ID: 32904	Proliferation	0.132	0.164		0.130	0.131	0.145	0.000	0.130	0.000	0.000	0.166	0.133	0.000	0.131	0.130	0.131
	Apoptosis	0.294	0.221		0.241	0.360	0.232	0.320	0.266	0.410	0.341	0.344	0.373	0.409	0.299	0.300	0.369
Patient ID: 11CO059	Proliferation	0.130	0.167		0.130	0.130	0.146	0.000	0.131	0.000	0.000	0.168	0.131	0.000	0.130	0.131	0.131
	Apoptosis	0.295	0.167		0.247	0.361	0.224	0.330	0.268	0.414	0.353	0.332	0.366	0.412	0.295	0.297	0.362
Patient ID: 12915	Proliferation	0.131	0.167		0.130	0.131	0.144	0.000	0.131	0.000	0.000	0.167	0.130	0.000	0.130	0.131	0.131
	Apoptosis	0.294	0.167		0.243	0.373	0.234	0.317	0.267	0.417	0.336	0.339	0.367	0.417	0.294	0.287	0.382
Patient ID: 32908	Proliferation	0.130	0.166		0.130	0.131	0.142	0.000	0.132	0.000	0.000	0.167	0.132	0.000	0.131	0.132	0.130
	Apoptosis	0.301	0.166		0.247	0.379	0.240	0.321	0.267	0.423	0.350	0.332	0.355	0.417	0.295	0.297	0.379
Patient ID: 05CO044	Proliferation	0.131	0.167		0.129	0.130	0.148	0.000	0.130	0.000	0.000	0.170	0.131	0.000	0.129	0.131	0.131
	Apoptosis	0.291	0.167		0.245	0.381	0.232	0.323	0.266	0.421	0.337	0.337	0.375	0.413	0.300	0.299	0.374
Patient ID: 32824	Proliferation	0.131	0.163		0.131	0.130	0.149	0.000	0.131	0.000	0.000	0.168	0.130	0.000	0.132	0.129	0.130
	Apoptosis	0.293	0.220		0.242	0.366	0.232	0.326	0.266	0.426	0.338	0.329	0.368	0.417	0.304	0.287	0.370
Patient ID: 32360	Proliferation	0.130	0.167		0.130	0.130	0.151	0.000	0.130	0.000	0.000	0.167	0.132	0.000	0.130	0.131	0.132
	Apoptosis	0.302	0.168		0.245	0.348	0.230	0.323	0.268	0.412	0.358	0.339	0.380	0.418	0.300	0.292	0.356
Patient ID: 05CO015	Proliferation	0.131	0.167		0.132	0.131	0.143	0.000	0.129	0.000	0.000	0.165	0.129	0.000	0.131	0.130	0.131
	Apoptosis	0.297	0.167		0.248	0.360	0.238	0.319	0.266	0.418	0.343	0.331	0.368	0.419	0.293	0.292	0.372
Patient ID: 32832	Proliferation	0.130	0.178		0.131	0.130	0.141	0.000	0.131	0.000	0.000	0.168	0.131	0.000	0.131	0.131	0.131
	Apoptosis	0.299	0.178		0.246	0.359	0.220	0.319	0.267	0.416	0.351	0.333	0.379	0.407	0.288	0.300	0.365

Patients ID's from cBioPortal	Cell Fates	Control	Mutated	Human Gene IDs	SOCS	ZBED	BIRC	MAP2K7	STK4	TPR		JUN	YAP1	LATS1	IRS	TLE	PIK3CA	AKT	FOXO	MYC
				Network Node Names	socse	DREF	DILPS	Hep	HpoSal	Bsk	Puc	Ap	Yki	Wts	InR	Chico	Dp	AKT	Foxo	dMyc
Generic	Proliferation	0.129	0.173		0.135	0.130	0.130	0.130	0.160	0.130	0.142	0.142	0.130	0.168	0.130	0.130	0.132	0.131	0.133	0.000
	Apoptosis	0.294	0.173		0.273	0.297	0.286	0.292	0.194	0.294	0.261	0.257	0.298	0.170	0.290	0.289	0.291	0.300	0.289	0.328
Patient ID: 01CO001	Proliferation	0.130	0.120		0.137	0.129	0.130	0.131	0.158	0.130	0.143	0.144	0.129	0.168	0.131	0.129	0.131	0.131	0.131	0.000
	Apoptosis	0.291	0.265		0.260	0.293	0.293	0.292	0.212	0.291	0.254	0.251	0.292	0.169	0.292	0.295	0.288	0.297	0.310	0.339
Patient ID: 32904	Proliferation	0.132	0.164		0.139	0.130	0.129	0.130	0.152	0.131	0.142	0.142	0.130	0.166	0.129	0.130	0.131	0.130	0.129	0.000
	Apoptosis	0.294	0.221		0.267	0.292	0.295	0.297	0.213	0.304	0.244	0.250	0.295	0.176	0.288	0.298	0.295	0.299	0.306	0.337
Patient ID: 11CO059	Proliferation	0.130	0.167		0.137	0.132	0.132	0.129	0.158	0.131	0.143	0.139	0.132	0.166	0.131	0.129	0.130	0.131	0.129	0.000
	Apoptosis	0.295	0.167		0.258	0.296	0.304	0.292	0.199	0.296	0.256	0.255	0.300	0.164	0.294	0.299	0.309	0.298	0.299	0.331
Patient ID: 12915	Proliferation	0.131	0.167		0.140	0.131	0.130	0.131	0.157	0.130	0.141	0.143	0.130	0.168	0.132	0.129	0.132	0.133	0.131	0.000
	Apoptosis	0.294	0.167		0.262	0.293	0.288	0.289	0.203	0.289	0.270	0.245	0.296	0.174	0.300	0.297	0.299	0.295	0.300	0.327
Patient ID: 32908	Proliferation	0.130	0.166		0.134	0.129	0.130	0.131	0.158	0.130	0.141	0.143	0.130	0.167	0.130	0.130	0.132	0.131	0.130	0.000
	Apoptosis	0.301	0.166		0.258	0.304	0.294	0.294	0.194	0.292	0.255	0.252	0.290	0.172	0.299	0.303	0.296	0.292	0.296	0.334
Patient ID: 05CO044	Proliferation	0.131	0.167		0.138	0.131	0.131	0.131	0.157	0.131	0.144	0.142	0.131	0.168	0.131	0.131	0.129	0.130	0.131	0.000
	Apoptosis	0.291	0.167		0.261	0.290	0.292	0.290	0.211	0.293	0.258	0.263	0.291	0.173	0.304	0.297	0.298	0.296	0.297	0.330
Patient ID: 32824	Proliferation	0.131	0.163		0.140	0.131	0.129	0.130	0.158	0.131	0.141	0.138	0.130	0.169	0.129	0.130	0.133	0.132	0.131	0.000
	Apoptosis	0.293	0.220		0.262	0.291	0.290	0.311	0.201	0.298	0.263	0.269	0.287	0.159	0.304	0.292	0.305	0.297	0.297	0.336
Patient ID: 32360	Proliferation	0.130	0.167		0.134	0.129	0.131	0.130	0.159	0.130	0.140	0.141	0.130	0.169	0.130	0.129	0.132	0.129	0.129	0.000
	Apoptosis	0.302	0.168		0.271	0.300	0.299	0.297	0.195	0.304	0.268	0.258	0.299	0.162	0.296	0.291	0.299	0.298	0.291	0.333
Patient ID: 05CO015	Proliferation	0.131	0.167		0.145	0.132	0.130	0.130	0.156	0.129	0.142	0.139	0.130	0.167	0.131	0.131	0.130	0.131	0.130	0.000
	Apoptosis	0.297	0.167		0.259	0.292	0.286	0.299	0.202	0.299	0.255	0.263	0.296	0.178	0.288	0.303	0.293	0.288	0.298	0.326
Patient ID: 32832	Proliferation	0.130	0.178		0.139	0.130	0.132	0.132	0.156	0.131	0.143	0.143	0.131	0.168	0.131	0.130	0.130	0.131	0.131	0.000
	Apoptosis	0.299	0.178		0.253	0.292	0.301	0.297	0.204	0.288	0.270	0.256	0.297	0.171	0.295	0.301	0.302	0.288	0.299	0.334

Supplementary Table 18: Personalized therapeutic combinations for individual patients against genes in PanDrugs database.

Patient	Generic																					
Efficacious Nodes	armdecad	dad	mad	punt	suh	apc arm	egfs	rolled	wg	pros	nicd	fz	snail	tcflef	notch	state	ral	delta	dome	Raf	AKT	dMyc
Targets from PanDrugs	CDH1		SMAD2	ACVR2A	RBPJ	APC	EGF	MAPK1	WNT1	PROS1	NOTCH1	FZD1	SNAI1	TCF7L2		STAT1	RASA1	DLL1		RAF1	AKT1	MYC
	CDH2		SMAD3	ACVR2B							NOTCH2	FZD2				STAT2	RASA3	DLL3		ARAF	AKT2	
	CDH3		SMAD4								NOTCH3	FZD3				STAT3		DLL4			AKT3	
	CDH5		SMAD7								NOTCH4	FZD4				STAT4						
	CDH7											FZD5				STAT5A						
	CDH11											FZD6				STAT5B						
	CDH17											FZD7				STAT6						
	CDH19											FZD8										
												FZD9										
												FZD10										

Patient	Patient ID: 01CO001																										
Efficacious Nodes	armdecad	dad	mad	punt	suh	hid	apcarm	egfs	rolled	dpp	wg	pros	nicd	fz	mir	snail	tcflef	notch	state	ral	delta	Raf	DREF	Yki	Chico	Foxo	dMyc
Targets from PanDrugs	CDH1		SMAD4	ACVR2A	RBPJ		APC	EGF	MAPK1		WNT1	PROS1	NOTCH1	FZD1		SNAI1	TCF7L2		STAT1	RASA1	DLL1	RAF1		YAP1	TLE1	FOXO1	MYC
	CDH2			ACVR2B									NOTCH2	FZD2					STAT2	RASA3	DLL3				TLE3	FOXO3	
	CDH3												NOTCH3	FZD3					STAT3		DLL4					FOXO4	
	CDH5												NOTCH4	FZD4					STAT4								
	CDH7													FZD5					STAT5A								
	CDH11													FZD6					STAT5B								
	CDH17													FZD7					STAT6								
	CDH19													FZD8													
														FZD9													
														FZD10													

Patient	Patient ID: 32904																									
Efficacious Nodes	arm decad	dad	mad	punt	suh	hid	apcarm	egfs	rolled	wg	pros	nicd	fz	snail	tcflef	notch	state	ral	delta	dome	Raf	DILPS	Bsk	InR	Foxo	dMyc
Targets from PanDrugs	CDH1		SMAD4	ACVR2A	RBPJ		APC	EGF	MAPK1	WNT1	PROS1	NOTCH1	FZD1	SNAI1	TCF7L2		STAT1	RASA1	DLL1		RAF1	BIRC	TPR	IRS1	FOXO1	MYC
	CDH2			ACVR2B								NOTCH2	FZD2				STAT2	RASA3	DLL3					IRS2	FOXO3	
	CDH3											NOTCH3	FZD3				STAT3		DLL4						FOXO4	
	CDH5											NOTCH4	FZD4				STAT4									
	CDH7												FZD5				STAT5A									
	CDH11												FZD6				STAT5B									
	CDH17												FZD7				STAT6									
	CDH19												FZD8													
													FZD9													
													FZD10													

Patient	Patient ID: 11CO059																												
Efficacious Nodes	armd ecad	dad	mad	punt	suh	hid	apcarm	der	egfs	rolled	wg	pros	nicd	arm	fz	snail	tcflef	notch	state	ral	delta	Raf	DILPS	Hep	Yki	Chico	Dp	Foxo	dMyc
Targets from PanDrugs	CDH1		SMA	ACV	RBPJ		APC	EGFR	EGF	MAP	WNT	PROS	NOT	CTN	FZD6	SNAI	TCF7		STAT	RASA	DLL1	RAF1	BIRC	MAP	YAP1	TLE1	PIK3	FOX	MYC
	CDH2		SMA												FZD7						DLL3					TLE3			
	CDH3		SMA												FZD9						DLL4								
	CDH5		SMA																										
	CDH7																												
	CDH1																												
	CDH1																												
	CDH1																												

Patient	Patient ID: 12915																							
Efficacious Nodes	dlg	armd ecad	dad	mad	punt	suh	apcar m	egfs	rolle d	wg	pros	nicd	arm	snail	tcflef	notc h	state	ral	delta	Raf	InR	Chic o	Foxo	dMy c
Targets from PanDrugs	DLG1	CDH1		SMA D7	ACV R2A	RBPJ	APC	EGF	MAP K1	WNT 1	PROS 1	NOT CH1	CTN NB1	SNAI 1	TCF7 L2		STAT 1	RASA 1	DLL1	ARAF	IRS1	TLE1	FOX O1	MYC
					ACV R2B							NOT CH3					STAT 2	RASA 3	DLL3		IRS2	TLE3	FOX O3	
																	STAT 3		DLL4				FOX O4	
																	STAT 4							
																	STAT 5A							
																	STAT 5B							
																	STAT 6							

Patient	Patient ID: 32908																						
Efficacious Nodes	armd ecad	dad	mad	punt	suh	hid	apcar m	egfs	rolle d	wg	pros	nicd	fz	snail	tcflef	notc h	state	ral	delta	Raf	DREF	Chic o	dMy c
Targets from PanDrugs	CDH2		SMA D4	ACV R2B	RBPJ		APC	EGF	MAP K1	WNT 1	PROS 1	NOT CH3	FZD1	SNAI 1	TCF7 L2		STAT 1	RASA 1	DLL1	ARAF	IRS1	TLE1	MYC
	CDH1 1												FZD6				STAT 2	RASA 3	DLL3			TLE3	
													FZD9				STAT 3		DLL4				
																	STAT 4						

Patient	Patient ID: 05CO044																										
Efficacious Nodes	armd ecad	dad	mad	punt	suh	hid	apcarm	egfs	rolled	dpp	wg	pros	nicd	arm	fz	mir	snail	tcflef	notch	state	ral	delta	upds	Raf	InR	Dp	dMyc
Targets from PanDrugs	CDH1		SMAD2	ACVR2A	RBPJ		APC	EGF	MAPK1		WNT1	PROS1	NOTCH2	CTNNB1	FZD2		SNAI1	TCF7L2		STAT1	RASA1	DLL1		RAF1	IRS1	PIK3C2A	MYC
	CDH2		SMAD3	ACVR2B									NOTCH3		FZD5					STAT2	RASA3	DLL3		ARAF	IRS2		
	CDH3		SMAD4																	STAT3		DLL4					
	CDH5		SMAD7																	STAT4							
	CDH7																			STAT5A							
	CDH11																			STAT5B							
	CDH17																			STAT6							
	CDH19																										

Patient	Patient ID: 32824																								
Efficacious Nodes	dlg	armd ecad	dad	mad	punt	suh	apcar m	egfs	rolle d	wg	pros	nicd	fz	snail	tcflef	notc h	state	ral	delta	upds	dom e	Raf	InR	Dp	dMy c
Targets from PanDrugs	DLG1	CDH1		SMA D4	ACV R2A	RBPJ	APC	EGF	MAP K1	WNT 1	PROS 1	NOT CH1	FZD1	SNAI 1	TCF7 L2		STAT 5A	RASA 1	DLL1			RAF1	IRS1	PIK3 C2A	MYC
		CDH2			ACV R2B							NOT CH2	FZD2				STAT 5B	RASA 3	DLL3			ARAF	IRS2		
		CDH3										NOT CH3	FZD3						DLL4						
		CDH5										NOT CH4	FZD4												
		CDH7											FZD5												
		CDH1 1											FZD6												
		CDH1 7											FZD7												
		CDH1 9											FZD8												
													FZD9												
													FZD1 0												

Patient	Patient ID: 32360																											
Efficacious Nodes	dlg	armd ecad	dad	mad	punt	suh	apcarm	egfs	rolled	wg	pros	nicd	arm	fz	snail	tcflef	notch	state	ral	delta	upds	Raf	DREF	Bsk	Chico	AKT	Foxo	dMyc
Targets from PanDrugs	DLG1	CDH5		SMAD2	ACVR2A	RBPJ	APC	EGF	MAPK1	WNT1	PROS1	NOTCH1	CTNNB1	FZD9	SNAI1	TCF7L2		STAT4	RASA1	DLL1		RAF1	IRS1	TPR	TLE1	AKT1	FOXO1	MYC
		CDH7										NOTCH2						STAT5A	RASA3	DLL3		ARAF			TLE3	AKT2	FOXO3	
												NOTCH3						STAT5B		DLL4						AKT3	FOXO4	
												NOTCH4						STAT6										

Patient	Patient ID: 05CO015																							
Efficacious Nodes	armdecad	dad	mad	punt	suh	apcarm	egfs	rolled	wg	pros	nicd	arm	fz	snail	tcflef	robo	notch	state	ral	delta	Raf	Bsk	Chico	dMyc
Targets from PanDrugs	CDH1		SMAD2	ACVR2A	RBPJ	APC	EGF	MAPK1	WNT1	PROS1	NOTCH1	CTNNB1	FZD1	SNAI1	TCF7L2	ROBO2		STAT1	RASA1	DLL1	BRAF	TPR	TLE1	MYC
			SMAD3	ACVR2B							NOTCH2		FZD2					STAT2	RASA3	DLL3			TLE3	
			SMAD4								NOTCH3		FZD3					STAT3		DLL4				
			SMAD7								NOTCH4		FZD4					STAT4						
													FZD5					STAT5A						
													FZD6					STAT5B						
													FZD7					STAT6						
													FZD8											
													FZD9											
													FZD10											

Patient	Patient ID: 32832																									
Efficacious Nodes	dlg	armd ecad	dad	mad	punt	suh	apcar m	egfs	rolle d	wg	pros	nicd	arm	fz	snail	tcflef	notc h	state	ral	delta	dom e	Raf	DILP S	Chic o	Dp	dMy c
Targets from PanDrugs	DLG1	CDH1 1		SMA D2	ACV R2A	RBPJ	APC	EGF	MAP K1	WNT 1	PROS 1	NOT CH1	CTN NB1	FZD1 0	SNAI 1	TCF7 L2		STAT 6	RASA 1	DLL1		BRAF	BIRC	TLE1	PIK3 C2A	MYC
				SMA D3	ACV R2B							NOT CH2							RASA 3	DLL3				TLE3		
				SMA D4								NOT CH3								DLL4						
				SMA D7								NOT CH4														

Supplementary Table 19: Potential efficacious nodes queried in PanDrugs database.

Nodes Queried in PanDrugs										
Generic	01CO001	32904	11CO059	12915	32908	05CO044	32824	32360	05CO015	32832
CDH1	CDH1	CDH1	CDH1	DLG1	CDH2	CDH1	DLG1	DLG1	CDH1	DLG1
CDH2	CDH2	CDH2	CDH2	CDH1	CDH11	CDH2	CDH1	CDH5	SMAD2	CDH11
CDH3	CDH3	CDH3	CDH3	SMAD7	SMAD4	CDH3	CDH2	CDH7	SMAD3	SMAD2
CDH5	CDH5	CDH5	CDH5	ACVR2A	ACVR2B	CDH5	CDH3	SMAD2	SMAD4	SMAD3
CDH7	CDH7	CDH7	CDH7	ACVR2B	RBPJ	CDH7	CDH5	ACVR2A	SMAD7	SMAD4
CDH11	CDH11	CDH11	CDH11	RBPJ	APC	CDH11	CDH7	RBPJ	ACVR2A	SMAD7
CDH17	CDH17	CDH17	CDH17	APC	EGF	CDH17	CDH11	APC	ACVR2B	ACVR2A
CDH19	CDH19	CDH19	CDH19	EGF	MAPK1	CDH19	CDH17	EGF	RBPJ	ACVR2B
SMAD2	SMAD4	SMAD4	SMAD2	MAPK1	WNT1	SMAD2	CDH19	MAPK1	APC	RBPJ
SMAD3	ACVR2A	ACVR2A	SMAD3	WNT1	PROS1	SMAD3	SMAD4	WNT1	EGF	APC
SMAD4	ACVR2B	ACVR2B	SMAD4	PROS1	NOTCH3	SMAD4	ACVR2A	PROS1	MAPK1	EGF
SMAD7	RBPJ	RBPJ	SMAD7	NOTCH1	FZD1	SMAD7	ACVR2B	NOTCH1	WNT1	MAPK1
ACVR2A	APC	APC	ACVR2A	NOTCH3	FZD6	ACVR2A	RBPJ	NOTCH2	PROS1	WNT1
ACVR2B	EGF	EGF	RBPJ	CTNNB1	FZD9	ACVR2B	APC	NOTCH3	NOTCH1	PROS1
RBPJ	MAPK1	MAPK1	APC	SNAI1	SNAI1	RBPJ	EGF	NOTCH4	NOTCH2	NOTCH1
APC	WNT1	WNT1	EGFR	TCF7L2	TCF7L2	APC	MAPK1	CTNNB1	NOTCH3	NOTCH2
EGF	PROS1	PROS1	EGF	STAT1	STAT1	EGF	WNT1	FZD9	NOTCH4	NOTCH3
MAPK1	NOTCH1	NOTCH1	MAPK1	STAT2	STAT2	MAPK1	PROS1	SNAI1	CTNNB1	NOTCH4
WNT1	NOTCH2	NOTCH2	WNT1	STAT3	STAT3	WNT1	NOTCH1	TCF7L2	FZD1	CTNNB1
PROS1	NOTCH3	NOTCH3	PROS1	STAT4	STAT4	PROS1	NOTCH2	STAT4	FZD2	FZD10
NOTCH1	NOTCH4	NOTCH4	NOTCH3	STAT5A	RASA1	NOTCH2	NOTCH3	STAT5A	FZD3	SNAI1
NOTCH2	FZD1	FZD1	CTNNB1	STAT5B	RASA3	NOTCH3	NOTCH4	STAT5B	FZD4	TCF7L2
NOTCH3	FZD2	FZD2	FZD6	STAT6	DLL1	CTNNB1	FZD1	STAT6	FZD5	STAT6
NOTCH4	FZD3	FZD3	FZD7	RASA1	DLL3	FZD2	FZD2	RASA1	FZD6	RASA1
FZD1	FZD4	FZD4	FZD9	RASA3	DLL4	FZD5	FZD3	RASA3	FZD7	RASA3
FZD2	FZD5	FZD5	SNAI1	DLL1	ARAF	SNAI1	FZD4	DLL1	FZD8	DLL1
FZD3	FZD6	FZD6	TCF7L2	DLL3	IRS1	TCF7L2	FZD5	DLL3	FZD9	DLL3
FZD4	FZD7	FZD7	STAT2	DLL4	TLE1	STAT1	FZD6	DLL4	FZD10	DLL4
FZD5	FZD8	FZD8	RASA3	ARAF	TLE3	STAT2	FZD7	RAF1	SNAI1	BRAF

FZD6	FZD9	FZD9	DLL1	IRS1	MYC	STAT3	FZD8	ARAF	TCF7L2	BIRC
FZD7	FZD10	FZD10	DLL3	IRS2		STAT4	FZD9	IRS1	ROBO2	TLE1
FZD8	SNAI1	SNAI1	DLL4	TLE1		STAT5A	FZD10	TPR	STAT1	TLE3
FZD9	TCF7L2	TCF7L2	RAF1	TLE3		STAT5B	SNAI1	TLE1	STAT2	PIK3C2A
FZD10	STAT1	STAT1	BIRC	FOXO1		STAT6	TCF7L2	TLE3	STAT3	MYC
SNAI1	STAT2	STAT2	MAP2K7	FOXO3		RASA1	STAT5A	AKT1	STAT4	
TCF7L2	STAT3	STAT3	YAP1	FOXO4		RASA3	STAT5B	AKT2	STAT5A	
STAT1	STAT4	STAT4	TLE1	MYC		DLL1	RASA1	AKT3	STAT5B	
STAT2	STAT5A	STAT5A	TLE3			DLL3	RASA3	FOXO1	STAT6	
STAT3	STAT5B	STAT5B	PIK3C2A			DLL4	DLL1	FOXO3	RASA1	
STAT4	STAT6	STAT6	FOXO1			RAF1	DLL3	FOXO4	RASA3	
STAT5A	RASA1	RASA1	MYC			ARAF	DLL4	MYC	DLL1	
STAT5B	RASA3	RASA3				IRS1	RAF1		DLL3	
STAT6	DLL1	DLL1				IRS2	ARAF		DLL4	
RASA1	DLL3	DLL3				PIK3C2A	IRS1		BRAF	
RASA3	DLL4	DLL4				MYC	IRS2		TPR	
DLL1	RAF1	RAF1					PIK3C2A		TLE1	
DLL3	YAP1	BIRC					MYC		TLE3	
DLL4	TLE1	TPR							MYC	
RAF1	TLE3	IRS1								
ARAF	FOXO1	IRS2								
AKT1	FOXO3	FOXO1								
AKT2	FOXO4	FOXO3								
AKT3	MYC	FOXO4								
MYC		MYC								

Supplementary Table 20: Personalized therapeutic combinations for individual patients.

Patients ID's from cBioPort	Drug Combinations	Studies/In Clinical Trial
Generic	Paclitaxel-Regorafenib	Advanced Esophagogastric Cancer [NCT02406170, DOI:https://doi.org/10.1093/annonc/mdz247.124]
01CO001	Paclitaxel-Bortezomib	Metastatic Solid Tumors [NCT00030368, NCT00667641]
32904	Paclitaxel-Bortezomib	Metastatic Solid Tumors [NCT00030368, NCT00667641]
11CO059	Imatinib	Colon Cancer [27799652, NCT00041340, 30772447, 17721919]<br>Chronic Myeloid Leukemia [26859076]
12915	Docetaxel-Bortezomib	Metastatic Breast Cancer [18454159]<br>Metastatic Head and Neck Cancer [NCT00425750]<br>Non-Small Cell Lung Cancer [NCT00362882, 17409841, 17075122, DOI: 10.1200/jco.2005.23.16_suppl.7034]<br>Prostate Cancer [17317831]
32908	Paclitaxel-Regorafenib	Advanced Esophagogastric Cancer [NCT02406170, DOI:https://doi.org/10.1093/annonc/mdz247.124]
05CO044	Regorafenib	Metastatic Colorectal Cancer [32753954, 30728734, 23177514]
32824	Regorafenib	Metastatic Colorectal Cancer [32753954, 30728734, 23177514]
32360	Everolimus	Metastatic Colorectal Adenocarcinoma [23743569, NCT00419159]
05CO015	Paclitaxel-Bortezomib	Metastatic Solid Tumors [NCT00030368, NCT00667641]
32832	Paclitaxel-Imatinib	Metastatic Solid Tumors (breast cancer and soft tissue sarcomas [23014737]<br>Non-Small Cell Lung Cancer [NCT00408460, 23033932]<br>Ovarian Cancer [20944093, 24963404]

Supplementary Table 21: Detailed node interaction rules and experimental evidences supporting different interactions and logical functions Microtubule model regulations.

Microtubule							
**Source Nodes**	**Node Update Logic (TISON)**	**Regulation Type**	**Target Nodes**	**Biological Names**	**Node Update Logic Equation (TISON)**	**Description**	**Reference**
Dlg Fz	Dlg && !Fz	Signaling	Apc-Arm	Armadillo B-catenin	Apc-Arm = Dlg && !Fz	Dlg activates and Fz deactivates Apc [18387324, 7833051 21041448]	18387324, 21041448, 7833051
Integrin Rolled	Integrin || Rolled	Receptor-Ligand Signaling	Cdc42	Cell division control protein 42 homolog	Cdc42 = Integrin || Rolled	Cdc42 is activated by integrin [9658176]. PKC activates downstream Src which activates Rolled [19602257], therefore Rolled activates Cdc42, as Src activates Cdc42 [16449321]	9658176, 19602257, 16449321
GFs	GFs	Receptor-Ligand	Integrin	Integrin	Integrin = GFs	Integrins are activates by growth factors [26124879]	26124879
Rolled	!Rolled	Signaling	Dlg	Discs Large	Dlg = !Rolled	PKC inbitbits Dlg [19029932, 24648766], and PKC is activated by	19029932, 24648766, 15037605,

						Ras [15037605], which activates Rolled [Kegg EGFR Signaling], therefore, rolled inhibits Dlg. Further, the loss of Dlg is activated by activated Ras expression [19029932].	Kegg EGFR Signalin g, 190299 32, 275740 01, 165320 34, 190299 32
Upds	Upds	Receptor-Ligand	Dome	Domeless	Dome = Upds	Dome is activated when its ligand Upd binds	240587 67
Wg	Wg	Receptor-Ligand	Fz	Frizzled	Fz = Wg	Frizzled is receptor of WNT which activates wingless pathway	783305 1
Dome Socs36 E	!SocsE && Dome	Signaling	JAK	Hopscotch	JAK = !SocsE && Dome	When Upds bind to Dome, JAK get activated via phosphorylat ion. On the other hand, Socs36E inactivates JAK.	268075 80, 195637 63
Integri n Sty	Integri n && !Sty	Signaling	Ral	Ras-related protein, inferred as Ras in the modal	Ral = Integrin && !Sty	Sprouty inhibits the activity of Ras [24530508]. Ras activated	Kegg MAPK - Fly, 245305 08,

						by integrin [11956235]	11956235
Slit	Slit	Receptor-Ligand	Robo2	Robo2	Robo2 = Slit	Robo2 is a receptor of Slit	11239147
Ral JAK	Ral ||JAK	Signaling	Rolled	Erk MAP kinase	Rolled = Ral ||JAK	Ral (Ras-related protein in fly) activates Phl (Raf in humans) which goes on to activate Rolled by phosphorylating it [23484853]. Rolled gets activated by JAK [8793290, 28472194]	Kegg MAPK - Fly, 8793290, 28472194, 21622856
Stat92E	Stat92E	Signaling	Socs36E	Suppressor of cytokine signaling at 36E	Socs36E = Stat92E	Stat92E induces expression of Jak/Stat repressor, Socs36E	19563763
Rolled Stat92E	!Rolled || !Stat92E	Signaling	Stathmin	Stathmin	Stathmin = !Rolled || !Stat92E	Erk (rolled) inhibits stathmin [16720434]. Stat inhibit stathmin activity [19251695] by phosphorylating it	16720434, 19251695
Robo2	Robo2	Signaling	Abl	Tyrosine-protein	Abl = Robo2	Robo activates Abl	20139699,

				kinase ABL1		[20139699, 19812305]	19812305
Abl	Abl	Signaling	CLASP	CLIP-associated protein	CLASP = Abl	CLASP is activated by Abl [15207236]	15207236
Stathmin CLASP ApcArm	Stathmin || (!CLASP || !ApcArm)	Binding	Microtubule	Microtubule	Microtubule = Stathmin || (!CLASP || !ApcArm)	Apc [18387324] and CLASP [20708587] stabilize microtubules while stathmin destabilize microtubule [25100123]	18387324, 20708587, 25100123
Rolled Stat92E	Rolled || Stat92E	Signaling	CDK	CDK	CDK = Rolled || Stat92E	Wide variety of mitogenic signaling pathways such as MAPKs, and STATs upregulate CDKs [17188374, 21613412]	17188374, 21613412
JAK Rolled	JAK || Rolled	Signaling + Transcriptional	Stat92E	Marelle D-STAT	Stat92E = JAK || Rolled	JAK promotes activation and dimerization Stat92E. Mirror inhibits Stat92E [28045022]. Rolled (fly homolog of Erk) also activates	26807580, 19563763, 28045022, 18337767, 27742579

						Stat [27742579]	
Ral	Ral	Signaling	Raf	Rapidly Accelerate d Fibrosarco ma	Raf = Ral	Ras activates Raf [15035987]	150359 87
Rolled	Rolled	Transcripti onal	Sty	Sprouty	Sty = Rolled	Rolled activates Pointed which then induces expression of Sprouty	245305 08

Supplementary Table 22: Detailed node interaction rules and experimental evidences supporting different interactions and logical functions for Integrated (ISC+MT) model.

ISC + Microtubule Network						
**Source Nodes**	**Node Update Logic (TISON)**	**Target Nodes**	**Biological Names**	**Node Update Logic Equation (TISON)**	**Description**	**Reference**
Fz Arm-DECad Dlg	Dlg || (!ArmDECad && !Fz)	ApcArm	Armadillo B-catenin	ApcArm = Dlg || (!ArmDECad && !Fz)	Frizzled is receptor of WNT which activates wingless pathway by inhibting the formation of Apc-Arm. Thereby, Apc cannot degrade Arm. DE-Cad and Apc compete for binding with Arm (B-catenin) [7833051]. Dlg activates Apc [18387324, 21041448]	16443747, 7833051, 18387324, 21041448
Apc-Arm Arm-DECad	!ApcArm && !ArmDECad	Arm	Armadillo B-catenin	Arm = !ApcArm && !ArmDECad	DE-Cad and Apc compete for binding with Arm (B-catenin) [7833051]. Apc inhibits Arm [15169756]. DE-cad inhibit Arm [22174153]	15169756, 7833051, 22174153
Apc-Arm TCF_LEF Snail NICD	!Snail && (!ApcArm || !NICD) && TCFLEF	ArmDECad	Arm *Drosophila* E-Cadherin	ArmDECad = !Snail && (!ApcArm || !NICD) && TCFLEF	Snail which is a known repressor of DE-Cad [15983400]. Arm either binds to cadherin or Apc therefore they inhibit one another. Arm/TCF_LEF complex forms a transcription factor for expression of Arm-DECad (Wingless genes) [18617885, 22174153]. NICD increase so JNK	17587826, 16918415, 16720643, 22174153, 15983400, 27574001, 25226030, 22174153

					increase [27574001] and JNK increase so its downstream Ecad decrease [25226030, 22174153]	
Rolled Dlg Integrin	Integrin || Rolled || !Dlg	Cdc	Cell division control protein 42 homolog	Cdc = Integrin || Rolled || !Dlg	PKC activates downstream Src which activates Rolled [19602257], therefore Rolled activates Cdc42, as Src activates Cdc42 [16449321]. Par6 is destabilized by Dlg, Par6 activates Cdc42, therefore, Dlg inhibits Cdc42 [24648766]. Rasv12 cells activation of Cdc42 [19287376]. Cdc42 is activated by integrin [9658176].	16449321, 19602257, 24648766, 19287376
Mad	Mad	Dad	Daughters against dpp	Dad = Mad	Mad induces expression of Dad, a Dpp target gene.	10902180, 9335506
EGFs	EGFs	DER	Torpedo also known as EGFR receptor	DER = EGFs	Activated DER receptor activates Ral (fly hmolog of Ras) by phosphorylation. DER is a tyrosine kinase receptor and part of EGF receptor subfamily [1425358]	1425358
Rolled ArmDECad NICD	!Rolled || ArmDECad || NICD	Dlg	Discs Large	Dlg = !Rolled || ArmDECad || NICD	PKC inbitbits Dlg [19029932, 24648766], and PKC is activated by Ras[15037605], which activates Rolled[Kegg EGFR Signaling], therefore, rolled inhibits Dlg. Further,	19029932, 24648766, 15037605, Kegg EGFR Signaling, 19029932, 27574001, 16532034, 19029932

					the loss of Dlg is activated by activated Ras expression [19029932]. NICD activates JNK [27574001] and JNK activates Dlg [16532034], so NICD activates Dlg. ArmDECad activates Dlg [19029932]	
Upds	Upds	Dome	Domeless	Dome = Upds	Dome is activated when its ligand Upd binds	24058767
Wg	Wg	Fz	Frizzled	Fz = Wg	Frizzled is receptor of WNT which activates wingless pathway	7833051
Rolled Foxo NICD	!Rolled || !Yki	Hid	Head involution defective Wrinkled	Hid = !Rolled || !Yki	Post-translational modifications of Hid prevent Hid-induced apoptosis [9814704]. For e.g. Rolled inhibts Hid by phosphorylating it [17183370]. Yorkie has anti-apoptotic function which inhibits apoptosis inhibiting gene hid [32485126] Yorkie inhibits bantam and bantem activates hid therefore, yorkie inhibits hid [31331981].	19554451, 17183370, 32485126, 9814704, 28102471, 31331981
Dome Socs36E	!SocsE && Dome	JAK	Hopscotch	JAK = !SocsE && Dome	When Upds bind to Dome, JAK get activated via phosphorylation. On the other hand, Socs36E inactivates JAK.	26807580, 19563763

Punt Dad	!Dad && Punt	Mad	Mothers against dpp	Mad = !Dad && Punt	Activated Punt phosphorylate Mad to activate it [24813173]. On the other hand, Dad antagonizes Mad activity [10902180]	24813173, 10902180, 9335506
TCF_LEF Rolled	TCFLEF || Rolled	Mirror	Mirror	Mirror = TCFLEF || Rolled	GRK activates EGFR which activates Rolled which inhibits CIC which then inhibts mirror, therefore, Rolled indirectly activates mirror[24127599, 27593379] also it is activated in EGFR signaling [10742112] . TCF activates Cyclin D1 [26721396], Cyclin D activates VEGF[16899588]. VEGF activates Rolled [20406854] which activates Mirror. Hence TCF_LEF activates Mirror.	24127599, 27593379, 10742112, 26721396, 16899588, Kegg VEGF Signaling, 20406854
Notch	Notch	NICD	N^{intra} Cleaved Intracellular Domain of Notch	NICD = Notch	Activated Notch undergoes cleavages to form NICD. Wnt inhibits Notch signaling [18806781].	17362357, 20816404, 18806781
Delta	Delta	Notch	Notch	Notch = Delta	Notch is receptor of Delta	7813766
Su(H) Stat92E Robo2	(!StatE || !SuH) && !Robo	Pros	Prospero	Pros = (!StatE || !SuH) && !Robo	Robo2 inhibits prospero activity [24931602]. Su(H) induces expression of E(spl) bHLH genes that produce bHLH transcriptional inhibitors that repress activity of achaete-scute factors [20147375].	24931602, 7600969, 7958894, 24931602, s41598-017-01138-z, 20147375, 25298397

					Scute_Asense factors induce expression of Prospero [24931602]. Escargot represses Scute mediated transcription of Prospero [s41598-017-01138-z, 7600969]. However, Stat92E has been implicated in delayed repression of Escargot potentially at the level of transcription [25298397].	
Dpp	Dpp	Punt	Punt	Punt = Dpp	Punt is a receptor of Dpp	21546910
DER Sty Integrin	(Integrin || DER) && !Sty	Ral	Ras-related protein, inferred as Ras in the modal	Ral = (Integrin || DER) && !Sty	Activated DER receptor activates Ral (fly hmolog of Ras) by phosphorylation. Sprouty inhibits the activity of Ras [24530508]. Sprouty inhibits the activity of Ras [24530508]. Ras activated by integrin [11956235]	Kegg MAPK - Fly, 24530508, 11956235
Slit	Slit	Robo	Robo2	Robo = Slit	Robo2 is a receptor of Slit	11239147
Ral JAK NICD	Ral ||JAK || NICD	Rolled	Erk MAP kinase	Rolled = Ral ||JAK || NICD	Ral (Ras-related protein in fly) activates Phl (Raf in humans) which goes on to activate Rolled by phosphorylating it [23484853]. Both JAK and NICD activate EGFR which activates Rolled. So Rolled gets activated by JAK [8793290, 28472194]	Kegg MAPK - Fly, 8793290, 28472194, 21622856

					and NICD as well [21622856].	
TCF_LEF Rolled NICD	TCFLEF || Rolled || NICD	Snail	Snail	Snail = TCFLEF || Rolled || NICD	Rolled activates Snail [28415812]. TCF is needed for Snail activation [23029025]. NICD also activates Snail [29705809, 15197341]	28415812, 23029025, 29705809, 15197341
StatE	StatE	SocsE	Suppresso r of cytokine signaling at 36E	SocsE = StatE	Stat92E induces expression of Jak/Stat repressor, Socs36E	19563763
Rolled Stat92E	!Rolled || !StatE	Stath min	Stathmin	Stathmin = !Rolled || !StatE	Erk (rolled) inhibits stathmin [16720434]. Stat inhibit stathmin activity [19251695] by phosphorylating it	16720434, 19251695
Robo2	Robo	Abl	Tyrosine-protein kinase ABL1	Abl = Robo	Robo activates Abl [20139699, 19812305]	20139699, 19812305
Abl	Abl	CLASP	CLIP-associated protein	CLASP = Abl	CLASP is activated by Abl [15207236]	15207236
Stathmin CLASP ApcArm CDK	Stathmi n || CDK || (!CLASP || !ApcAr m)	Microt ubule	Microtubu le	Microtubul e = Stathmin || CDK || (!CLASP || !ApcArm)	Apc [18387324] and CLASP [20708587] stabilize microtubules while stathmin destabilize microtubule [25100123]	18387324, 20708587, 25100123
Rolled StatE	Rolled || StatE	CDK	CDK	CDK = Rolled || StatE	Wide variety of mitogenic signaling pathways such as MAPKs, and STATs upregulate CDKs [17188374, 21613412]	17188374, 21613412

JAK Mirror Rolled	JAK || !Mirror || Rolled	StatE	Marelle D-STAT	StatE = JAK || !Mirror || Rolled	JAK promotes activation and dimerization Stat92E. Mirror inhibits Stat92E [28045022]. Rolled (fly homolog of Erk) also activates Stat [27742579]	26807580, 19563763, 28045022, 18337767, 27742579
Rolled	Rolled	Sty	Sprouty	Sty = Rolled	Rolled activates Pointed which then induces expression of Sprouty	24530508
NICD Mad Stat92E	(!StatE || !Mad) && NICD	SuH	Suppresso r of Hairless	SuH = (!StatE || !Mad) && NICD	NICD promotes activation of Su(H) mediated transcription of Notch genes by removing repression by Hairless [17362357]. Mad inhibits Su(H) activity [23861806, (28945500, inferred)]. Stat92E promotes expression of Hairless [26758761, 24077308] which acts a repressor of Su(H) [20147375].	28945500, 17362357, 20147375, 26758761, 24077308, 23861806
Ral	Ral	Raf	Rapidly Accelerate d Fibrosarco ma	Raf = Ral	Ras activates Raf [15035987]	15035987
Arm NICD	Arm || NICD	TCFLEF	T-cell factor/ lymphocyt e enhancer factor-1/ pangolin	TCFLEF = Arm || NICD	Arm binds to and activates TCF/LEF transcription factors. NICD activates TCFLEF [28245235, 26592459]	Kegg WNT Signaling Fly, 26592459, 28245235
DREF	DREF	Hep	hemiptero us	Hep = DREF	DREF activates Hep which in turn activates bsk	24752236

DREF	DREF	HpoSal	Salvador	HpoSal = DREF	DREF activates Hippo pathway such that it inhibits yki	25424907
Hep Puc	Hep ||!Puc	Bsk	basket	Bsk = Hep ||!Puc	Bsk is activates by Hep and inhibited by Puc	10.3934/genet.2014.1.20, 11402332
AP1	AP	Puc	puckered	Puc = AP	AP1 activates puckered	9472024, 11402332
Bsk	Bsk	Ap	Jun-related antigen	Ap = Bsk	Bsk compete to phosphorylate jun (ap1)	8946915, 11402332
Wts	!Wts	Yki	Yorkie	Yki = !Wts	Wts phosphorylates Yki and inhibits its transcriptional activity	17889654, 21808241
Ral Bsk HpoSal	!Bsk || HpoSal	Wts	warts	Wts = !Bsk || HpoSal	Hippo directly interacts with sav to activate wts [12941273, 21808241]. Wts is inhibited by Bsk [28174264]	12941273, 21808241, 28174264
DILPs	DILPs	InR	Insulin-like receptor	InR = DILPs	ILP1 activates InR1	22252538
InR	InR || Foxo	Chico	chico	Chico = InR || Foxo	Foxo activates Chico and InR activates Chico	30055320
Chico	Chico || Ral	Dp	Pi3K92E Dp110	Dp = Chico || Ral	Chico and Ral activates PI3K92E (Dp110) [30055320, 26119340]	30055320
Dp110	Dp	AKT	AKT1	AKT = Dp	PI3K (Dp110) activates its downstream target AKT [26508828]	26508828
AKT	!AKT	Foxo	forkhead box	Foxo = !AKT	Akt inhibits foxo through direct phosphorylation	21440577
AKT Rolled	AKT || Rolled || tcflef	dMyc	Myc	dMyc = AKT || Rolled || tcflef	AKT and TCFLEF promotes activation of myc [15241468, 31623618]. Rolled (erk) enables myc	15241468, 22461507, 31623618

					stabilization [22461507]	

Supplementary Table 23: Results from targeted therapy of colorectal cancer patients.

Chemotherapy of Colorectal Cancer Patients								
Sr #	Type of Therapy	Patients ID's from cBioPortal	Type of Screening	Drug Combinations	Apoptosis	% increase	Proliferation	% decrease
0	Generic Therapy	Generic	Mutations		0.000	100	0.210	100
			Therapy	Paclitaxel-Regorafenib<br>Microtubule + [AKT3, APC, ARAF, EGF, RAF1, RASA1, SMAD2, SMAD4 and STAT3]	0.327		0.000	
1	Personalized Therapy	01CO001	Mutations		0.000	100	0.150	100
			Therapy	Paclitaxel-Bortezomib<br>Microtubule + [CDH1, CDH11, MAPK1, NOTCH1, RAF1, RASA1, STAT1 and STAT3]	0.461		0.000	
2		32904	Mutations		0.000	100	0.458	100
			Therapy	Paclitaxel-Bortezomib<br>Microtubule + [CDH1, CDH11, MAPK1, NOTCH1, RAF1, RASA1, STAT1 and STAT3]	0.220		0.000	
3		11CO059	Mutations		0.000	100	0.200	100
			Therapy	Imatinib<br>[CTNNB1, EGF, EGFR, MYC, SMAD2 and SMAD4]	0.000		0.000	
4		12915	Mutations		0.000	100	0.200	100
			Therapy	Docetaxel-Bortezomib<br>Microtubule + [CDH1, MAPK1, NOTCH1, RASA1, STAT1 and STAT3]	0.462		0.000	
5		32908	Mutations		0.000	100	0.200	100
			Therapy	Paclitaxel-Regorafenib<br>Microtubule + [APC, ARAF, EGF, RASA1, SMAD4 and STAT3]	0.332		0.000	
6		05CO044	Mutations		0.000	100	0.200	100
			Therapy	Regorafenib<br>[APC, ARAF, EGF, RAF1, RASA1, SMAD2, SMAD4 and STAT3]	0.000		0.000	
7		32824	Mutations		0.000	100	0.219	100
			Therapy	Regorafenib<br>[APC, ARAF, EGF, RAF1, RASA1 and SMAD4]	0.000		0.000	
8		32360	Mutations		0.000	100	0.200	100
			Therapy	Everolimus<br>[AKT1, AKT2, AKT3, CTNNB1, IRS1, MAPK1 and NOTCH1]	0.000		0.000	
9		05CO015	Mutations		0.000	100	0.200	100
			Therapy	Paclitaxel-Bortezomib<br>Microtubule + [BRAF, CDH1, MAPK1, NOTCH1, RASA1, ROBO2, STAT1 and STAT3]	0.366		0.000	
10		32832	Mutations		0.000	100	0.219	100
			Therapy	Paclitaxel-Imatinib<br>Microtubule + [BRAF, CTNNB1, EGF, MYC, SMAD2 and SMAD4]	0.334		0.000	

Supplementary Table 24: Tutorial for performing analysis for the study.

Description	Link
**Supplementary data link**	https://github.com/BIRL/DrosophilaPatientModel
**01-How to download supplementary data for onwards analysis**	https://youtu.be/CjD0vm1IRt0
**02-How to upload network file, and perform robustness analysis**	https://youtu.be/sGmwjeIgA0k
**03-How to perform network analysis and view results**	https://youtu.be/NSBvSQYhGJs
**04-How to upload therapeutic steps and perform analysis**	https://youtu.be/TreQFhZtFJc

Supplementary Table 25: Mapping of cell fate classification logic

Cell Fate	Rolled		Cdc		Hid		Dlg		TCFLEF		ApcArm		StatE		SuH		Pros		Mad		dMyc	
EE Fate																	1	[2]				
EB Fate															1;2	[3]						
Upd Production															2	[4]						
Dpp Production																			2	[14]		
Delta Production													1	[5]	0	[4]						
Multilayering											0	[6]										
Normal Proliferation	1	[7]							1	[8]			1	[9]							1	[15]
Loss of Polarity							0	[1]														
Apoptosis					1;2	[11]																
Extrusion			1	[10]																		
EC Fate													1	[13]	1	[12]						

Ref No.	PubMed ID	Explanation
[1]	22392736, http://hdl.handle.net/2445/55144	Dlg is a tumor suppressor gene, in cells lacking Dlg there is loss of polarity, If Dlg becomes delocalizaed from plasma membrane and diffuses in cytoplasm, cell loses its polarity
[2]	25670791	Pros is a marker of EE fate
[3]	22049341	Su(H) is marker for EB fate
[4]	28475577, 28928428, 16287856	Su(H) represses Notch by reducing Upd Production (inferred), Su(H) is required for both Notch activation and repression
[4]	12897132, 12154126	Su(H) represses Notch by reducing Delta Production (inferred)
[5]	23172913, 19563763	Stat92E will induce Delta production (delta is marker for ISC), Dome or Stat92E depletion in progenitors
[6]	23570874	formation of Multilayering accelerated after Apc loss.
[7]	25288756, 16684521	Rolled induces proliferation
[8]	27867007	TCF promotes proliferation
[9]	23172913, 19003442	Stat92E will promote prolifeation
[10]	28781348	Cdc42 mediate cell extrusion
[11]	9144202	Hid together with rpr can have a greater apoptotic rate then either alone
[12]	10673509, 22190634	Notch is needed for EC promotion and NICD activates Su(H)
[13]	19563763	Stat signaling promotes cell differentiation
[14]	9693372	Mad requires a partner to mediate Dpp signaling
[15]	14724122	dmyc is required for cell growth and proliferation

Supplementary Table 26: Details of tumor suppressors and oncogenes in ISC network.

TISON Network Names	Target protein for cancer therapy	Activity			Therapy in TISON
		Cancer	Therapy	Reference	
dlg	discs large 1	downregulated	Activation	http://hdl.handle.net/2445/55144	dlg -> 1
armdecad	Cadherin-N	downregulated	Activation	http://hdl.handle.net/2445/55144	armdecad -> 1
dad	Daughters against dpp	downregulated	Activation	http://hdl.handle.net/2445/55144	dad -> 1
jak	hopscotch	downregulated	Activation	http://hdl.handle.net/2445/55144	jak -> 1
mad	Mothers against dpp	downregulated	Activation	http://hdl.handle.net/2445/55144	mad -> 1
punt	punt	downregulated	Activation	http://hdl.handle.net/2445/55144	punt -> 1
suh	Suppressor of Hairless	downregulated	Activation	27404588/26302407/http://hdl.handle.net/2445/55144	suh -> 1
hid	Wrinkled	downregulated	Activation	26442596	hid -> 1
apcarm	Adenomatous polyposis coli	downregulated	Activation	http://hdl.handle.net/2445/55144	apcarm -> 1
der	Epidermal growth factor receptor	downregulated	Activation	http://hdl.handle.net/2445/55144	der -> 1
egfs	spitz	downregulated	Activation	18701096	egfs -> 0
rolled	rolled	downregulated	Activation	inferred	rolled -> 0
dpp	decapentaplegic	downregulated	Activation	OncoKB	dpp -> 0
slit	slit	downregulated	Activation	OncoKB	slit -> 0
wg	wingless	downregulated	Activation	9016631	wg -> 0

pros	prospero	downregulated\ Upregulated	Inhibition	16034367/http://hdl.handle.net/2445/55144	pros -> 0
nicd	Notchless	downregulated\ Upregulated	Activation	16273080	nicd -> 0
arm	armadillo	Upregulated	Inhibition	16034367	arm -> 0
fz	frizzled	Upregulated	Inhibition	30675292	fz -> 0
mir	mirror	Upregulated	Inhibition	16034367	mir -> 0
snail	snail	Upregulated	Inhibition	24931602	snail -> 0
sty	sprouty	Upregulated	Inhibition	16034367	sty -> 0
tcflef	pangolin	Upregulated	Inhibition	26759228	tcflef -> 0
robo	robo3	Upregulated	Inhibition	31894255/19020755	robo -> 0
notch	Notch	Upregulated	Inhibition	http://hdl.handle.net/2445/55144	notch -> 0
state	Signal-transducer at 92E	Upregulated	Inhibition	http://hdl.handle.net/2445/55144	stat -> 0
cdc	Cdc42	Upregulated	Inhibition	http://hdl.handle.net/2445/55144	cdc -> 0
ral	Ral interacting protein	Upregulated	Inhibition	http://hdl.handle.net/2445/55144	ral -> 0
delta	Delta	Upregulated	Inhibition	http://hdl.handle.net/2445/55144	delta -> 0
upds	unpaired 2	Upregulated	Inhibition	http://hdl.handle.net/2445/55144	upds -> 0
dome	domeless	Upregulated	Inhibition	31217826	dome -> 0
Raf	Raf	Upregulated	Inhibition	31894255/19020755	Raf -> 0
socse	Suppressor of cytokine at 36E	Upregulated	Inhibition	31677966	socs -> 0

DREF	DNA replication-related element factor	Upregulated	Inhibition	http://hdl.handle.net/2445/55144	dref -> 0
DILPS	Insulin-like peptide 1	Upregulated	Inhibition	http://hdl.handle.net/2445/55144	dilps-> 0
Hep	hemipterous	Upregulated	Inhibition	21577205	Hep -> 1
HpoSal	Salvador	Upregulated	Inhibition	20727758	hposal -> 0
Bsk	basket	Upregulated	Inhibition	inferred	bsk-> 0
Puc	puckered	Upregulated	Inhibition	OncoKB	puc-> 0
Ap	jun	Upregulated	Inhibition	29535423	ap-> 0
Yki	Yorkie	Upregulated	Inhibition	29793481	yki-> 0
Wts	warts	Upregulated	Inhibition	OncoKB	wts-> 0
InR	Insulin-like receptor	Upregulated	Inhibition	OncoKB	inr-> 0
Chico	Chico	Upregulated	Inhibition	OncoKB	chico-> 0
Dp	Pi3K92E	Upregulated\ downregulated	Inhibition	16034367/22863622	dp-> 0
AKT	AKT	Upregulated\ downregulated	Inhibition	16034367/http://hdl.handle.net/2445/55144	AKT -> 0
Foxo	Foxo	Upregulated\ downregulated	Inhibition	30453988/27148488	foxo-> 0
dMyc	Myc	Upregulated\ downregulated	Inhibition	27134635	dmyc-> 0

1.3. References

1. Lemaitre B, Miguel-Aliaga I. The digestive tract of Drosophila melanogaster. *Annu Rev Genet*. (2013) 47:377–404. doi: 10.1146/annurev-genet-111212-133343

2. Casali A, Batlle E. Intestinal stem cells in mammals and Drosophila. *Cell Stem Cell*. (2009) 4(2):124–7. doi: 10.1016/j.stem.2009.01.009
